# Modelling and rescuing neurodevelopmental defect of Down syndrome using induced pluripotent stem cells from monozygotic twins discordant for trisomy 21

**DOI:** 10.1002/emmm.201302848

**Published:** 2013-12-27

**Authors:** Youssef Hibaoui, Iwona Grad, Audrey Letourneau, M Reza Sailani, Sophie Dahoun, Federico A Santoni, Stefania Gimelli, Michel Guipponi, Marie Françoise Pelte, Frédérique Béna, Stylianos E Antonarakis, Anis Feki

**Affiliations:** 1 Stem Cell Research Laboratory, Department of Obstetrics and Gynecology, Geneva University HospitalsGeneva, Switzerland; 2 Department of Genetic Medicine and Development, University of Geneva Medical School and Geneva University HospitalsGeneva, Switzerland; 3 Department of Pathology and Immunology, Faculty of Medicine, University of GenevaGeneva, Switzerland; 4 iGE3 Institute of Genetics and Genomics of Geneva, University of GenevaGeneva, Switzerland; 5 Service de gynécologie obstétrique, HFR Fribourg—Hôpital CantonalFribourg, Switzerland

**Keywords:** disease modelling, Down syndrome, DYRK1A, induced pluripotent stem cells, neurodevelopment

## Abstract

Down syndrome (trisomy 21) is the most common viable chromosomal disorder with intellectual impairment and several other developmental abnormalities. Here, we report the generation and characterization of induced pluripotent stem cells (iPSCs) derived from monozygotic twins discordant for trisomy 21 in order to eliminate the effects of the variability of genomic background. The alterations observed by genetic analysis at the iPSC level and at first approximation in early development illustrate the developmental disease transcriptional signature of Down syndrome. Moreover, we observed an abnormal neural differentiation of Down syndrome iPSCs *in vivo* when formed teratoma in NOD-SCID mice, and *in vitro* when differentiated into neuroprogenitors and neurons. These defects were associated with changes in the architecture and density of neurons, astroglial and oligodendroglial cells together with misexpression of genes involved in neurogenesis, lineage specification and differentiation. Furthermore, we provide novel evidence that *dual-specificity tyrosine-(Y)-phosphorylation regulated kinase 1A* ( *DYRK1A*) on chromosome 21 likely contributes to these defects. Importantly, we found that targeting DYRK1A pharmacologically or by shRNA results in a considerable correction of these defects.

## Introduction

Down syndrome (DS) caused by a trisomy of chromosome 21 (HSA21), is the most common genetic developmental disorder, with an incidence of one in 800 live births. DS individuals show cognitive impairment, learning and memory deficits, arrest of neurogenesis and synaptogenesis, and early onset of Alzheimer's disease (AD; Antonarakis *et al*, 2004; Lott & Dierssen, 2010). The detailed pathogenetic mechanisms by which the extra copy of HSA21 leads to the neurodevelopmental DS phenotype remain unknown. High-resolution mapping of rare, partial trisomies of HSA21 has provided evidence that several regions exist on HSA21 with various ‘dosage sensitive’ genes contributing to a given phenotype, which could also be modified by other genes on HSA21 and in the rest of the genome (Lyle *et al*, 2008; Korbel *et al*, 2009).

Several approaches have been used to study the pathogenesis of DS. Mouse aneuploidies have been engineered to model the various DS phenotypes; these models display some behavioral, anatomical and cellular abnormalities similar to human phenotypes (reviewed in Das & Reeves, 2011). Additionally, studies with post-mortem human DS brains have allowed the investigation of neuroanatomical abnormalities and the alterations that occur during brain development (Becker *et al*, 1991; Mito & Becker, 1993; Guidi *et al*, 2008). Moreover, *in vitro* culture of neural progenitor cells (NPCs) isolated from brain of DS patients were informative in dissecting the mechanisms underlying brain defects (Bahn *et al*, 2002; Esposito *et al*, 2008; Bhattacharyya *et al*, 2009; Lu *et al*, 2011). The discovery that pluripotent stem cells (PSCs) can be induced from human adult somatic cells by the introduction of few factors and their extensive similarities to embryonic stem cells (ESCs; Takahashi *et al*, 2007; Yu *et al*, 2009) has provided new opportunities for investigation in human developmental biology and diseases. Recent studies have been successful in generating disease-specific induced pluripotent stem cells (iPSCs) from a variety of neurodevelopmental and neurodegenerative disorders (Park *et al*, 2008; Soldner *et al*, 2009; Brennand *et al*, 2011; Israel *et al*, 2012), providing excellent *in vitro* models for the pathophysiology and potential treatment of these disorders (Hibaoui & Feki, 2012).

In the present study, we have generated iPSCs from fetal fibroblasts of monozygotic twins discordant for trisomy 21: Twin-N-iPSCs for the normal iPSCs and Twin-DS-iPSCs for the iPSCs carrying the trisomy 21. The use of monozygotic twins has allowed us to study the effect of the supernumerary chromosome 21 without the biological ‘noise’ of the variation of the genome. We evaluated their multi-lineage potentials *in vivo* by teratoma formation when iPSCs were injected intramuscularly into immunodeficient SCID mice. The DS pathogenesis was further investigated when iPSCs were induced to differentiate into neural progenitor cells (NPCs) and neurons. Furthermore, we used Twin-DS-iPSCs to validate candidate genes involved in the impairment of neurogenesis described in DS patients. Among the numerous protein coding genes of HSA21, *dual-specificity tyrosine-(Y)-phosphorylation regulated kinase 1A* ( *DYRK1A*) encodes a proline-directed serine/threonine and tyrosine kinase which plays pleiotropic roles in neurodevelopment and disease (Tejedor *et al*, 1995; Guimera *et al*, 1996; Song *et al*, 1996; Becker *et al*, 1998; Tejedor & Hämmerle, 2011). Transgenic mice carrying an extra copy of *Dyrk1A* display neurodevelopmental delays, motor abnormalities, learning and memory deficits, and altered synaptic plasticity (Smith *et al*, 1997; Altafaj *et al*, 2001; Ahn *et al*, 2006). Based on these studies, we investigated the role of DYRK1A in Twin-DS-iPSC-derived NPCs and neurons, through its inhibition by pharmacological means or by short hairpin RNA silencing.

## Results

### Generation of iPSCs from monozygotic twins discordant for trisomy 21

**Figure 1:**
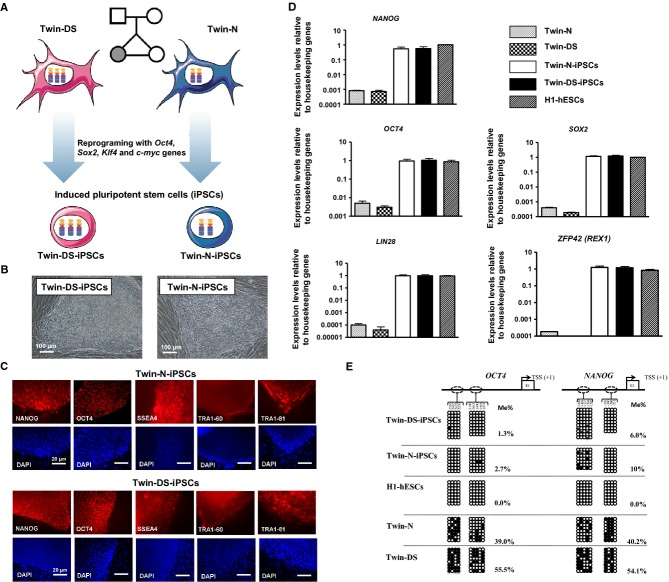
Schematic representation of Twin-N and Twin-DS parental fibroblasts reprogramming into Twin-N-iPSCs and Twin-DS-iPSCs using *OCT4*, *SOX2*, *KLF4* and *c-MYC* genesPhase contrast images of Twin-N-iPSCs and Twin-DS-iPSCs growing on feeder cells.Immunofluorescence staining of Twin-N-iPSC and Twin-DS-iPSC lines for pluripotency markers NANOG, OCT4, SSEA4, TRA1-60 and TRA1-80.qRT-PCR of pluripotency-related genes; *NANOG*, *OCT4*, *SOX2*, *LIN28* and *ZFP42* ( *REX1*). Data are represented as mean ± s.e.m. from *n* = 3.DNA methylation profile of *OCT4* and *NANOG* promoters. The global percentage of methylated cytosines (% Me) is indicated (open and closed circles indicate unmethylated and methylated CpGs, respectively).

Normal (Twin-N) and Down syndrome (Twin-DS) fetal fibroblasts were isolated from monozygotic twins discordant for trisomy 21 (Dahoun *et al*, 2008) and used to establish Twin-N-iPSCs and Twin-DS-iPSCs using *OCT4*, *SOX2*, *KLF4* and *c-MYC* genes as previously described (Takahashi *et al*, 2007; Grad *et al*, 2011; Fig 1A and B). All iPSC lines expressed markers of pluripotent cells including NANOG, OCT4, TRA-1-60, TRA-1-81 and SSEA4 as demonstrated by immunofluorescence staining (Fig 1C) and showed alkaline phosphatase activity (supplementary Fig S1A). Quantitative (qRT-PCR) and non quantitative RT-PCR analysis demonstrated expression of selected endogenous pluripotent transcription factors including *OCT4, SOX2, NANOG, LIN28 and ZFP42* ( *REX1*) in the generated iPSCs in contrast with the parental fibroblasts (Fig 1D and supplementary Fig S1B). As expected for cells that have acquired a pluripotent state, transgene silencing was verified after initial expansion of around 10 passages of Twin-N-iPSCs and Twin-DS-iPSCs (supplementary Fig S1B). Moreover, as in hESC-H1, *OCT4* and *NANOG* promoter regions of Twin-N-iPSCs and Twin-DS-iPSCs were found hypomethylated in comparison with their parental fibroblasts (Fig 1E).

### Transcriptome dysregulation of DS iPSCs

**Figure 2:**
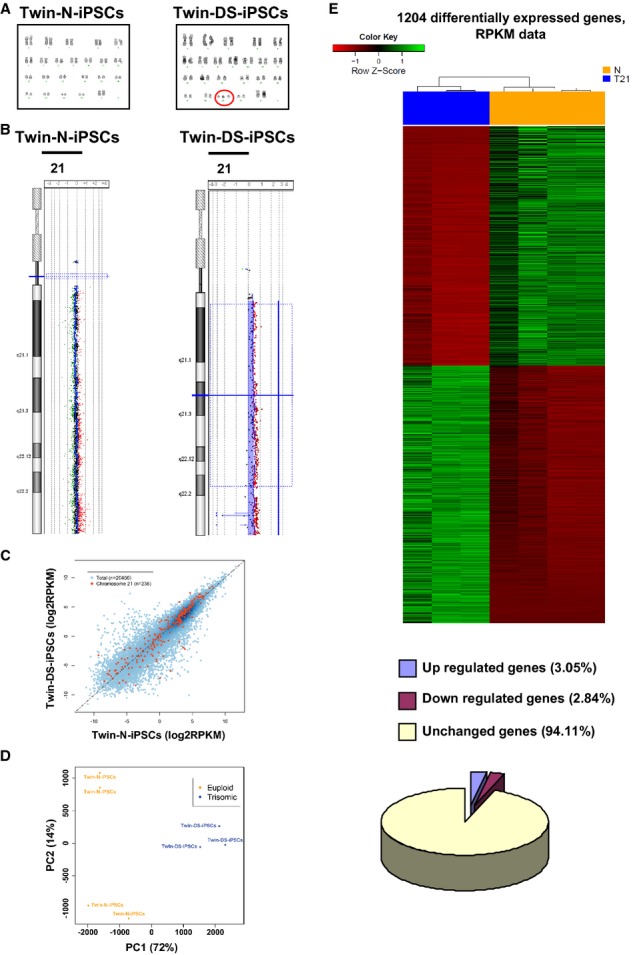
Karyotypes of Twin-N-iPSCs and Twin-DS-iPSCs are 46, XX and 47, XX+21, respectively.Ideogram and array-CGH profile showing the gain of one DNA copy of chromosome 21 in Twin-DS-iPSCs in comparison with Twin-N-iPSCs.Comparison of the normalized gene expression levels mean of log2 RPKM between Twin-N-iPSCs and Twin-DS-iPSCs (HSA21 genes are shown in red and the rest of the genome in blue).Principal component analysis (PCA) plot based on the normalized expression values in Twin-N-iPSCs and Twin-DS-iPSCs. The percentages represent the proportion of variance explained by each component.Heat map of the normalized gene expression values in Twin-DS-iPSCs and Twin N-iPSCs for the 1204 differentially expressed genes. A negative *z*-score (in red) indicates low expression (below the mean) whereas a positive *z*-score (in green) shows high expression (above the mean). Diagram showing the proportion of genes differentially expressed between Twin-N-iPSCs and Twin-DS-iPSCs. See also supplementary Table S2.

These iPSCs were evaluated to confirm the disease-specific genotype of their parental somatic cells. As revealed by karyotype and array-based comparative genomic hybridization analysis, Twin-DS-iPSCs showed the characteristic trisomy 21 while Twin-N-iPSCs had a normal karyotype (Fig 2A and B). Then, whole transcriptome analysis using mRNA-Sequencing confirmed that the majority of HSA21 genes are indeed more expressed in the trisomic lines than the euploid lines (Fig 2C and supplementary Fig S2A) which is consistent with the overall up-regulation of HSA21 genes in individuals with DS (Antonarakis *et al*, 2004; Lott & Dierssen, 2010). Principal component analysis (PCA) showed that the first principal component (PC1) clearly discriminated the trisomic from the euploid samples, explaining most of the variance in the whole genome data (72%) and in the analysis of HSA21 genes (91%; Fig 2D and supplementary Fig S2B).

Differential expression analysis was performed using the statistical R packages EdgeR and DESeq. We considered the genes reported as being differentially expressed by both packages thereby obtaining a conservative list of 580 downregulated and 624 upregulated genes expressed in Twin-DS-iPSCs (Bonferroni-corrected *P*-value < 0.01; Fig 2E and supplementary Table S2). To further characterize the biological processes that might be affected in Twin-DS-iPSCs, we investigated the functional annotations associated with the 1204 differentially expressed genes using DAVID (Database for Annotation, Visualization and Integrated Discovery). The gene ontology (GO) analysis of the 580 genes downregulated in Twin-DS-iPSCs revealed significant enrichment for genes involved in multiple developmental processes including embryonic development and morphogenesis, organ development and morphogenesis and system development. Among these genes, 96 genes were associated with nervous system-related terms and specifically with nervous system development, central and peripheral nervous system development, brain development, neurogenesis, generation of neurons, neuron differentiation and axonogenesis (Table 1 and supplementary Fig S4). The GO analysis also revealed enrichment for terms linked to cellular adhesion (i.e. biological adhesion, cell adhesion, cell-cell adhesion) and to the cadherin signalling pathway. Additionally, WNT signalling and cancer pathways were found associated with the list of downregulated genes. The GO analysis of the 624 genes upregulated in Twin-DS-iPSCs showed enrichment for functions related to the regulation of RNA metabolic processes, regulation of transcription and DNA-dependent transcription. Interestingly, an important number of zinc finger protein genes were upregulated in Twin-DS-iPSCs (Table 1 and supplementary Table S3).

**Table 1:** Gene ontology (GO) categories of biological processes in which significant differentially expressed genes were grouped

Category	Term	Count	%	*P*-value	Benjamini
**GO categories of biological processes asssociated with the 580 downregulated genes:**					
GO:0007275	Multicellular organismal development	189	33.6	1.10E-29	2.40E-26
GO:0048731	System development	165	29.3	1.60E-28	1.70E-25
GO:0032502	Developmental process	195	34.6	3.20E-27	2.30E-24
GO:0048856	Anatomical structure development	170	30.2	6.20E-27	3.40E-24
GO:0009653	Anatomical structure morphogenesis	103	18.3	8.20E-23	3.60E-20
GO:0007399	Nervous system development	95	16.9	2.30E-21	8.20E-19
GO:0048513	Organ development	125	22.2	3.40E-21	1.00E-18
GO:0032501	Multicellular organismal process	222	39.4	3.40E-21	9.30E-19
GO:0030154	Cell differentiation	117	20.8	1.70E-19	4.20E-17
GO:0048869	Cellular developmental process	118	21	1.50E-18	3.30E-16
GO:0007156	Homophilic cell adhesion	30	5.3	1.80E-17	3.60E-15
GO:0022610	Biological adhesion	68	12.1	1.90E-17	3.40E-15
GO:0007155	Cell adhesion	67	11.9	6.60E-17	1.90E-14
GO:0016337	Cell-cell adhesion	41	7.3	9.70E-17	1.70E-14
GO:0009887	Organ morphogenesis	54	9.6	1.50E-13	2.20E-11
GO:0007389	Pattern specification process	36	6.4	2.20E-13	3.00E-11
GO:0009888	Tissue development	59	10.5	2.40E-13	3.10E-11
GO:0001501	Skeletal system development	39	6.9	4.20E-13	5.10E-11
GO:0009790	Embryonic development	53	9.4	7.50E-13	8.50E-11
GO:0048598	Embryonic morphogenesis	37	6.6	2.90E-12	3.10E-10
GO:0022008	Neurogenesis	52	9.2	2.20E-11	2.30E-09
GO:0043009	Chordate embryonic development	37	6.6	2.60E-11	2.60E-09
GO:0009792	Embryonic development ending in birth or egg hatching	37	6.6	3.40E-11	3.20E-09
GO:0048699	Generation of neurons	48	8.5	1.90E-10	1.80E-08
GO:0006357	Regulation of transcription from RNA polymerase II promoter	56	9.9	2.70E-10	2.40E-08
GO:0050793	Regulation of developmental process	52	9.2	1.30E-09	1.10E-07
GO:0051239	Regulation of multicellular organismal process	63	11.2	3.80E-09	3.10E-07
GO:0030182	Neuron differentiation	39	6.9	5.10E-09	3.90E-07
**GO categories of biological processes asssociated with the 624 upregulated genes:**					
GO:0051252	Regulation of RNA metabolic process	106	2.1	2.40E-09	5.60E-06
GO:0006355	Regulation of transcription, DNA-dependent	104	2.1	3.10E-09	3.60E-06
GO:0006350	Transcription	117	2.4	4.60E-09	3.60E-06
GO:0009058	Biosynthetic process	169	3.4	4.20E-08	2.40E-05
GO:0044249	Cellular biosynthetic process	165	3.3	5.00E-08	2.30E-05
GO:0009059	Macromolecule biosynthetic process	140	2.8	1.60E-07	6.30E-05
GO:0045449	Regulation of transcription	131	2.6	1.80E-07	5.90E-05
GO:0034645	Cellular macromolecule biosynthetic process	139	2.8	1.80E-07	5.30E-05
GO:0051171	Regulation of nitrogen compound metabolic process	139	2.8	3.30E-07	8.60E-05
GO:0019219	Regulation of nucleobase, nucleoside, nucleotide and nucleic acid metabolic process	138	2.8	3.50E-07	8.00E-05

The table lists the biological processes that might be affected due to trisomy 21, GO categories are ranked by *P*-values and Benjamini-corrected *P*-values given by DAVID (see also supplementary Table S3). The number of genes differentially expressed in each category is also shown.

### *In vivo* and *in vitro* differentiation of normal and DS iPSCs

**Figure 3:**
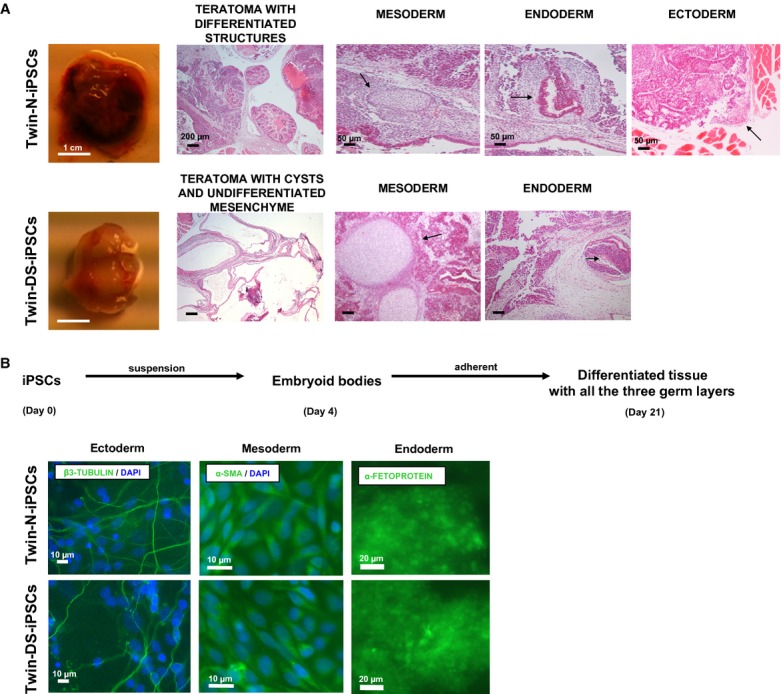
Hematoxylin and eosin staining analysis of teratoma generated after intramuscular injection of Twin-N-iPSCs (upper panel) and Twin-DS-iPSCs (lower panel) into SCID mice. See also supplementary Fig S4.Spontaneous *in vitro* differentiation of Twin-N-iPSCs (upper panel) and Twin-DS-iPSCs (lower panel) as embryoid bodies (EBs) in suspension culture for 4 days and as adherent cells for an additional 17 days. These EBs expressed α-SMA (mesoderm), AFP (endoderm) and β3-tubulin (ectoderm).

To document their developmental potential *in vivo*, iPSCs were injected intramuscularly into immunodeficient SCID mice. Histological analysis revealed that Twin-N-iPSCs formed teratoma with all embryonic germ layers: mesoderm, ectoderm and endoderm (Fig 3A upper panel and supplementary Fig S4). In contrast, injection of Twin-DS-iPSCs formed teratoma with multiple cysts which were surrounded by an abundant undifferentiated mesenchyme (Fig 3A lower panel). Mucinous intestinal-type differentiation or foci of bone were also found (Fig 3A lower panel). Surprisingly, differentiated ectoderm germ structures were not found in Twin-DS-iPSC-derived teratomas (Fig 3A lower panel). These results were confirmed by the near absence of staining for the neuroepithelial marker NESTIN in Twin-DS-iPSC-derived teratoma (supplementary Fig S4). We next determined that these iPSCs could differentiate *in vitro* into all three embryonic germ layers as detected by expression of the ectodermal marker β3-TUBULIN, the mesodermal marker α-SMOOTH MUSCLE ACTIN (α-SMA) and the endodermal marker α-FETOPROTEIN (AFP; Fig 3B).

### Proliferation deficit and increased apoptosis in NPCs derived from DS iPSCs

**Figure 4:**
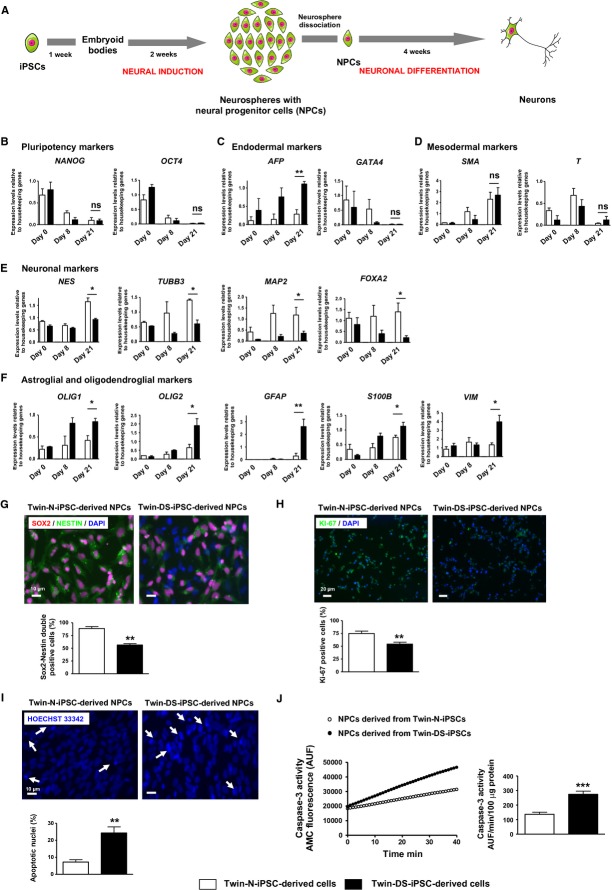
A Schematic representation of neural induction of the iPSCs into NPCs and neuronal differentiation into neurons. B–F qRT-PCR analysis of pluripotency, endodermal and mesodermal markers upon neural induction of Twin-N-iPSCs and Twin-DS-iPSCs into NPCs. The expression of neuronal, astroglial and oligodendroglial markers is also shown. Data are represented as mean ± s.e.m. For clarity, statistics are only shown at the NPC level (day 21). Ns non significant, * *P* < 0.05, ** *P* < 0.01 by Student's *t*-test from *n* = 3–5. G Proportion of SOX2/NESTIN double positive cells in NPCs derived from Twin-N-iPSCs and Twin-DS-iPSCs. See also supplementary Fig S5. H Cell proliferation analysis by Ki-67 staining of NPCs derived from Twin-N-iPSCs and Twin-DS-iPSCs. See also supplementary Fig S6. I Nuclear damage analysis of NPCs derived from Twin-N-iPSCs and Twin-DS-iPSCs (arrows, Hoechst 33342 staining). J Representative AMC fluorescence traces and quantification of caspase-3 activity in NPCs derived from Twin-N-iPSCs and Twin-DS-iPSCs. Data are represented as mean ± s.e.m. *** *P* < 0.001 by Student's *t*-test from *n* > 4. See also supplementary Fig S7.

IPSC lines were also characterized to confirm their differentiation potentials into neural progenitor cells (NPCs, day 21) *in vitro* through the protocol outlined in Fig 4A. As expected, the expression of the pluripotency markers *OCT4* and *NANOG* decreased in both iPSCs when induced to differentiate into NPCs (Fig 4B). Under these conditions, we next investigated the kinetics of emergence of neuronal and non-neuronal markers in these cells. Interestingly, our results showed that NPCs derived from Twin-DS-iPSCs expressed more *AFP* than those derived from Twin-N-iPSCs (Fig 4C). In contrast, we did not find any difference in the expression of the endodermal marker *GATA4* and the mesodermal markers *SMA* and *T* (Fig 4C and D). Moreover, the expression of the neuroepithelial precursor marker *NES* and the neuronal markers *TUBB3*, *MAP2* and *FOXA2* were lower in NPCs derived from Twin-DS-iPSCs than those derived from Twin-N-iPSCs (Fig 4E). In addition, Twin-DS-iPSC-derived cells exhibited a more astroglial phenotype as revealed by the greater expression of *GFAP*, *S100B* and *VIM* (Fig 4F). Also, we detected an increased expression of the basic helix-loop-helix transcription factors and oligodendroglial markers *OLIG1* and *OLIG2* in Twin-DS-iPSC-derived NPCs (Fig 4F).

To test whether the neurogenesis defect is associated with a decrease of NPC population in DS neurospheres, we analyzed the number of NESTIN and SOX2 double positive cells in dissociated neurospheres derived from Twin-N-iPSCs and Twin-DS-iPSCs. Importantly, we found a significant decrease of NESTIN/SOX2 double positive cells in Twin-DS-iPSC-derived NPCs (88.9 ± 3.8 versus 56.4 ± 2.9%, *P* < 0.0001 for neurospheres derived from Twin-N-iPSCs and Twin-DS-iPSCs, respectively; Fig 4G), indicating a reduced number of NPCs in Twin-DS-iPSC-derived neurospheres. Further analysis revealed an increased proportion of committed astroglial cells (2.4 ± 0.6 versus 13.0 ± 1.6%, *P* = 0.0009 for GFAP^+^ cells in neurospheres derived from Twin-N-iPSCs and Twin-DS-iPSCs, respectively) and oligodendroglial cells (3.7 ± 2.0 versus 11.7 ± 0.9%, *P* = 0.0055 for OLIG2^+^ cells in neurospheres derived from Twin-N-iPSCs and Twin-DS-iPSCs, respectively) in neurospheres derived from Twin-DS-iPSCs (supplementary Fig S5B). The proportion of committed neuronal cells had a tendency to be higher in neurospheres derived from Twin-DS-iPSCs but failed to reach significance (5.6 ± 0.9 versus 14.7 ± 3.6%, *P* = 0.089 for β3-TUBULIN^+^ cells in neurospheres derived from Twin-N-iPSCs and Twin-DS-iPSCs, respectively; supplementary Fig S5B). Next, we assessed whether the defective neurogenesis in DS is due to proliferation deficits and/or increased cell death of NPCs. As shown in Fig 3H, the proportion of Ki-67 positive cells is significantly lower in Twin-DS-iPSC-derived NPCs consistent with a reduced proliferation of Twin-DS-iPSC-derived NPCs. Moreover, NPCs derived from Twin-DS-iPSCs showed an approximately threefold increase of the number of apoptotic nuclei together with an approximately twofold increase of caspase-3 activity compared with those derived from Twin-N-iPSCs (Fig 4I and J). This proliferation deficit and increased apoptosis affected principally NESTIN positive cells as revealed by an approximately twofold decrease of the number of cells double positive for NESTIN and Ki-67 and an approximately four-and six-fold increase of the number of cells double positive for NESTIN and cleaved caspase-3 in neurospheres derived from Twin-DS-iPSCs (supplementary Figs S6 and S7).

### Altered maturation of NPCs derived from DS iPSCs into neurons

**Figure 5:**
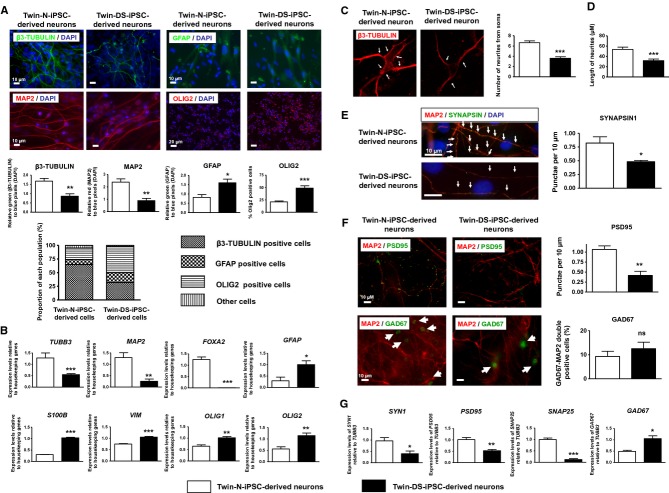
A Quantitative expression of neuronal (β3-TUBULIN and MAP2), astroglial (GFAP) and oligodendroglial (OLIG2) markers after maturation of NPCs derived from Twin-N-iPSCs and Twin-DS-iPSCs into neurons, by immunofluorescence analysis. The proportion of β3-TUBULIN, GFAP and OLIG2 positive cells is also shown. Data are represented as mean ± s.e.m. * *P* < 0.05, ** *P* < 0.01, *** *P* < 0.001 by Student's *t*-test from *n* > 4. B qRT-PCR analysis of neuronal ( *TUBB3*, *MAP2* and *FOXA2*), astroglial and oligodendroglial ( *GFAP*, *S100B*, *VIM*, *OLIG1* and *OLIG2*) markers upon neuronal differentiation of NPCs into neurons. Data are represented as mean ± s.e.m. * *P* < 0.05, ** *P* < 0.01, *** *P* < 0.001 by Student's *t*-test from *n* = 4. C Representative images and quantitative analysis of neurites (either axons or dendrites) from the soma of β3-TUBULIN positive neurons derived from Twin-N-iPSCs and Twin-DS-iPSCs. Data are represented as mean ± s.e.m. *** *P* < 0.001 by Student's *t*-test from *n* = 4. D Quantitative analysis of the length of neurites of neurons derived from Twin-N-iPSCs and Twin-DS-iPSCs. Data are represented as mean ± s.e.m. *** *P* < 0.001 by Student's *t*-test from *n* = 4. E, F Quantitative analysis of MAP2 positive neurons derived from Twin-N-iPSCs and Twin-DS-iPSCs stained for SYNAPSIN1 (in E), PSD95 and GAD67 (in F). Data are represented as mean ± s.e.m. Ns non significant, * *P* < 0.05, ** *P* < 0.01 by Student's *t*-test from *n* = 3–4. G qRT-PCR of the synaptic markers *SYN1*, *PSD95*, *SNPA25* and *GAD67* in neurons derived from Twin-N-iPSCs and Twin-DS-iPSCs. Data are represented as mean ± s.e.m. * *P* < 0.05, ** *P* < 0.01, *** *P* < 0.001 by Student's *t*-test from *n* = 3–5.

When NPCs were further induced to mature into neurons, we found a decreased expression of β3-TUBULIN and MAP2, and an increase of GFAP, VIMENTIN, S100B, OLIG1 and OLIG2 markers in Twin-DS-iPSC-derived cells when compared with Twin-N-iPSC-derived cells, as revealed by immunofluorescence staining and qRT-PCR (Fig 5A and B). These results strongly support an impaired neurogenesis together with a shift from neuronal to astroglial and oligodendroglial phenotype in Twin-DS-iPSC-derived cells. Notably, this shift was still observed even when a greater number of NPCs was induced to mature into neurons which rules out the possibility that the reduced number of NPCs derived from Twin-DS-iPSCs could lead to the observed shift from neuronal to astroglial and oligodendroglial phenotype (unpublished observations). Quantification of neurite (either axons or dendrites) branching from soma of β3-TUBULIN positive neurons showed that the mean number of branches was approximately twofold lower in neurons derived from Twin-DS-iPSCs than those derived from Twin-N-iPSCs (Fig 5C). Thus, neurons derived from Twin-DS-iPSCs demonstrated reduced length of neurites (Fig 5D).

An impaired synaptic maturation together with an imbalance between excitatory and inhibitory synapses have been proposed to explain the cognitive impairment in DS (Kleschevnikov *et al*, 2004; Belichenko *et al*, 2007; Chakrabarti *et al*, 2007; Martínez-Cué *et al*, 2013). Therefore, we investigated the expression of both pre-and post-synaptic markers in neurons by immunofluorescence staining and qRT-PCR analysis. MAP2-positive dendrites in neurons derived from Twin-DS-iPSCs exhibited a reduced density of SYNAPSIN punctae (Fig 5E) and a reduced expression of *SYN1* and *SNAP25* transcripts (Fig 5G). We next investigated the proportion of GABA-ergic and glutamatergic neurons derived from Twin-N-iPSCs and Twin-DS-iPSCs. Interestingly, we found a reduced density of PSD95, a post-synaptic protein expressed in glutamatergic neurons and a lower expression of *PSD95* transcripts in neurons derived from Twin-DS-iPSCs (Fig 5F and G). In contrast, we found a greater expression of *GAD67* (which encodes an enzyme responsible for the synthesis of GABA in neurons) in Twin-DS-iPSC-derived neurons (Fig 5G). The expression of GAD67 protein had a tendency to be higher in neurons derived from Twin-DS-iPSCs but failed to reach significance (Fig 5F).

### Rescue of neurogenesis impairment of NPCs and neurons derived from DS iPSCs through DYRK1A inhibition

**Figure 6:**
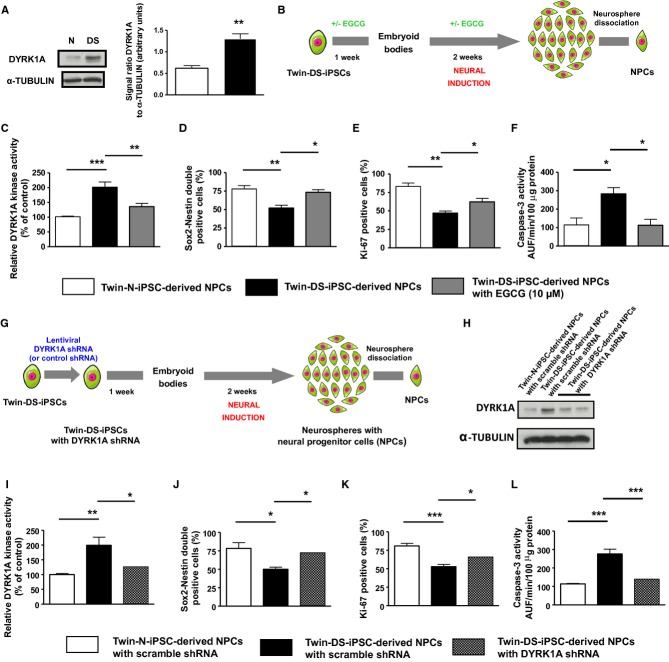
A Expression and activity of DYRK1A in NPCs derived from Twin-N-iPSCs and Twin-DS-iPSCs. Data are represented as mean ± s.e.m. ** *P* < 0.01 by Student's *t*-test from *n* = 4. B Schematic representation for generation of NPCs from Twin-DS-iPSCs after incubation with EGCG 10 μM. C Activity of DYRK1A in NPCs derived from Twin-N-iPSCs and Twin-DS-iPSCs. The effect of 10 μM EGCG is also shown. Data are represented as mean ± s.e.m. ** *P* < 0.01, *** *P* < 0.001 by one-way ANOVA followed with Tukey's test from *n* = 5. D–F Effect of DYRK1A inhibition by EGCG on the number (in D), the proliferation (in E, Ki-67 staining) and cell death (in F, caspase-3 activity) of NPCs derived from Twin-DS-iPSCs. Data are represented as mean ± s.e.m. * *P* < 0.05, ** *P* < 0.01 by one-way ANOVA followed with Tukey's test from *n* = 4. G Schematic representation for generation of NPCs from Twin-DS-iPSCs after transduction with lentiviruses encoding shRNAs targeting *DYRK1A*. H–L Expression (in H) and activity (in I) of DYRK1A in NPCs derived from Twin-N-iPSCs, Twin-DS-iPSCs and Twin-DS-iPSCs with DYRK1A shRNA. Effect of DYRK1A inhibition by shRNA on the number (in J), the proliferation (in K, Ki-67 staining) and cell death (in L, caspase-3 activity) of NPCs derived from Twin-DS-iPSCs. Data are represented as mean ± s.e.m. * *P* < 0.05, ** *P* < 0.01, *** *P* < 0.001 by one-way ANOVA followed with Tukey's test from *n* = 4.

**Figure 7:**
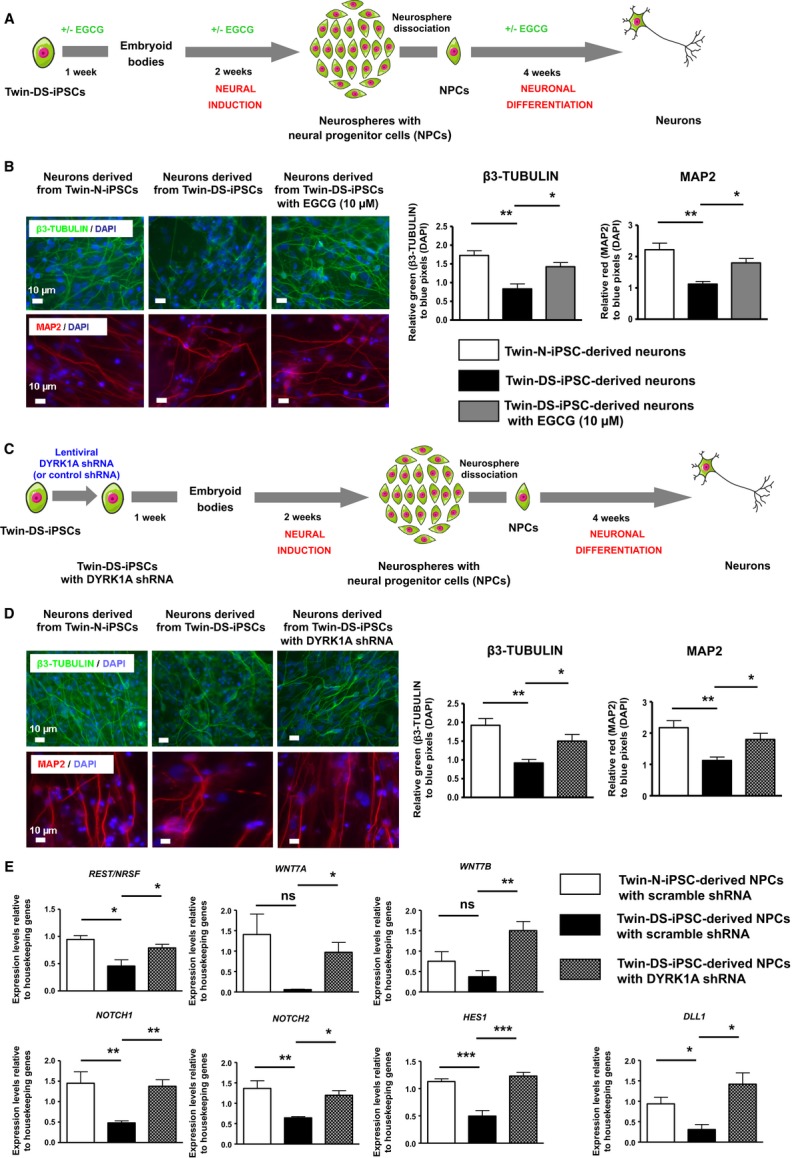
Schematic representation for generation of neurons from Twin-DS-iPSCs after incubation with EGCG 10 μM.Effect of DYRK1A inhibition by EGCG on the expression of neuronal markers upon neuronal differentiation of Twin-DS-iPSCs. Data are represented as mean ± s.e.m. * *P* < 0.05, ** *P* < 0.01 by one-way ANOVA followed with Tukey's test from *n* = 4.Schematic representation for generation of neurons from Twin-DS-iPSCs after transduction with lentiviruses encoding shRNAs targeting *DYRK1A*.Effect of DYRK1A inhibition through shRNA silencing on the expression of neuronal markers upon neuronal differentiation of Twin-DS-iPSCs. Data are represented as mean ± s.e.m. * *P* < 0.05, ** *P* < 0.01 by one-way ANOVA followed with Tukey's test from *n* = 4.qRT-PCR of *REST/NRSF*, *WNT7A*, *WNT7B*, *NOTCH1*, *NOTCH2*, *HES1* and *DLL1* in NPCs derived from Twin-N-iPSCs and Twin-DS-iPSCs. The effect of DYRK1A inhibition by shRNA is also shown. Data are represented as mean ± s.e.m. * *P* < 0.05, ** *P* < 0.01, *** *P* < 0.001 by one-way followed with Tukey's test from *n* = 4–7.

Considering that *DYRK1A* is involved in neurodevelopment and altered in DS (Tejedor *et al*, 1995; Guimera *et al*, 1996; Song *et al*, 1996; Tejedor & Hämmerle, 2011), we investigated the role of *DYRK1A* in NPCs and neurons derived from Twin-DS-iPSCs. As shown in Fig 6A, an approximately twofold increase of DYRK1A protein was found in Twin-DS-iPSC-derived NPCs (see also supplementary Fig S8). Moreover, treatment of Twin-DS-iPSCs during neural induction into NPCs with the selective DYRK1A inhibitor epigallocatechine gallate (EGCG, 10 μM) resulted in a significant inhibition of DYRK1A activity which reached almost the value of control (Fig 6B and C). Therefore, we tested whether DYRK1A inhibition by EGCG treatment during neural induction and neuronal differentiation of Twin-DS-iPSCs, can affect the defective neurogenesis of these cells. Notably, EGCG treatment improved the number of NPCs derived from Twin-DS-iPSCs by promoting proliferation and preventing apoptosis (Fig 6D–F). To provide additional support for the role of DYRK1A in these defects and to exclude a possible antioxidant effect of EGCG, knockdown of DYRK1A was achieved using short hairpin RNA (shRNA) in Twin-DS-iPSCs (Fig 6G–I and supplementary Fig S6). Importantly, this knockdown improved the number of NPCs derived from Twin-DS-iPSCs by promoting proliferation and preventing apoptosis (Fig 6J–L). When these NPCs were further induced to mature into neurons, DYRK1A inhibition through EGCG treatment or shRNA improved the expression of the neuronal markers β3-TUBULIN and MAP2, indicative of an improvement of neurogenesis (Fig 7A–D).

To explore the possible mechanisms underlying these improvements, we further investigated the expression of crucial genes and pathways involved in neural fate and differentiation namely *REST/NRSF* (Kuwabara *et al*, 2004; Ballas *et al*, 2005), *WNT* and *NOTCH* signalling pathways (Ciani & Salinas, 2005; Ables *et al*, 2011). Consistent with a previous report (Canzonetta *et al*, 2008), qRT-PCR clearly showed that *REST/NRSF* expression was reduced by approximately 30% in Twin-DS-iPSC-derived NPCs in comparison with Twin-N-iPSC-derived NPCs (Fig 7E). Moreover, we investigated the expression of *NOTCH1* and *NOTCH2* receptors, the NOTCH ligand *DLL1* and the NOTCH target *HES1*. As shown in Fig 7E, NPCs derived from Twin-DS-iPSCs exhibited a 2.9-and 2.1-fold reduction of *NOTCH1* and *NOTCH2* respectively, as well as an approximately twofold reduction of *DLL1* and *HES1*, in comparison with those derived from Twin-N-iPSCs. Similarly, the near absence of expression of *WNT7A* in NPCs derived from Twin-DS-iPSCs resulted in a 28-fold reduction of it expression compared to those derived from Twin-N-iPSCs (Fig 7E). Thus, *WNT7B* was also found downregulated in these cells (Fig 7E). Importantly, restoring *DYRK1A* expression in Twin-DS-iPSC-derived NPCs to near normal levels by shRNA, promoted the expression of these genes (Fig 7E).

## Discussion

A major challenge in the field of DS research has been to recapitulate the disease features and to understand the molecular mechanisms by which the extra copy of HSA21 genes leads to the abnormalities observed in DS patients. Many attempts have been made to identify HSA21 genes involved in the cognitive impairment in DS patients. For instance, several studies reported genotype-phenotype correlations in DS using genetic analysis (Lyle *et al*, 2008; Korbel *et al*, 2009); however, the molecular mechanisms underlying most of the DS phenotypes remain unknown. In order to recapitulate the disease phenotype *in vitro*, both ESCs and iPSCs have been derived from embryos and patients with DS (Park *et al*, 2008; Biancotti *et al*, 2010; Shi *et al*, 2012). Shi *et al* reported a PSC model of AD pathology using DS iPSCs. Neurons derived from DS iPSCs exhibited greater secretion of amyloid peptides, tau protein phosphorylation and cell death, supporting the notion that DS iPSCs are an excellent model for AD study (Shi *et al*, 2012). To our best knowledge, our study is the first demonstrating a neurodevelopmental phenotype in human iPSC-derived neural population. Twin-N-iPSCs and Twin-DS-iPSCs were derived from monozygotic twins discordant for trisomy 21 and thus confounding effects from genomic variability were theoretically eliminated. Twin monozygosity and discordance for trisomy 21 anomaly were tested by cytogenetic, genotyping and BAC-array analysis in the case-report published by Dahoun and colleagues (Dahoun *et al*, 2008). Since the rest of the genome is identical between the two samples, we hypothesized that these twins were ideal to study the effect of the supernumerary HSA21.

Several observations and findings arise from the current study investigating the cellular and molecular characteristics of neural cells derived from Twin-DS-iPSCs. In this regard, transcriptional profiling has proven extremely informative for the study of many diseases (Brennand *et al*, 2011; Israel *et al*, 2012). First, the genetic profiling of Twin-DS-iPSCs confirms that trisomy 21 not only affected the expression of trisomic genes but also the expression of disomic genes in Twin-DS-iPSCs, which is consistent with the argument that trisomy 21 leads to the alteration of whole transcriptome (Lyle *et al*, 2008). Among these genes, we identified 96 downregulated genes related to brain-related functions (supplementary Fig S4). Then, network analysis further implicates several expected and new pathways involving developmental processes (including those related to neurogenesis), cancer pathways, regulation of transcription, zinc finger (Znf)-C2H2 type genes and cadherins (Fig 2F, Table S3 and supplementary Fig S9). Collectively, the observation of these alterations in Twin-DS-iPSCs illustrates the transcriptional signature of the early developmental abnormalities of DS.

Another major finding of this study is that Twin-DS-iPSCs demonstrated an abnormal *in vivo* and *in vitro* neural differentiation. In particular, the teratoma formation assay revealed an abnormal *in vivo* differentiation of Twin-DS-iPSCs into neurectodermal tissue. In line with this, Mensah *et al* (2007) showed that the introduction of HSA21 into a transchromosomic mouse ESC line caused the suppression of *in vivo* differentiation into neurectoderm in a teratoma formation assay. Moreover, by tracking the neural induction and differentiation of iPSCs, it was possible to identify a phenotypic disturbance during early neural development of Twin-DS-iPSCs. In comparison with neurospheres derived from Twin-N-iPSCs, those derived from Twin-DS-iPSCs showed a reduced number of NPCs that was associated with a proliferation deficit and an increased apoptosis. These results are consistent with the increased rate of apoptosis and decreased proliferation capacity observed in brains from DS patients and mouse models of DS (Contestabile *et al*, 2007; Guidi *et al*, 2008; Lu *et al*, 2011).

Furthermore NPCs derived from Twin-DS-iPSCs revealed a decreased expression of neuronal markers and an increased expression of glial markers. In this respect, numerous morphometric studies have shown a lower neuronal density in several parts of human brains including the hippocampal dendate gyrus, the hippocampus, the parahippocampal gyrus and the cerebellum (Guidi *et al*, 2008, 2011). Our results are further supported by studies showing an increased ratio of astrocytes/neurons in human DS brain (Mito & Becker, 1993; Guidi *et al*, 2008). In NPCs isolated from brain of DS fetuses, there is evidence both in favor and against the idea that trisomy 21 favors glial at the expense of neuronal differentiation. These discrepancies may be attributable to the brain region origin, the different gestational ages of the fetal tissue and the methodologies used for isolation, maintenance and differentiation of these NPCs. For instance, Bahn and colleagues have shown a reduced number of neurons differentiated from fetal DS NPCs but revealed no difference in the number of astroglial cells (Bahn *et al*, 2002). In another study, fetal DS NPCs showed no alterations at early stages of neural development but only at later stages. In particular, these NPCs generated fewer neurons and showed increased expression of oligodendroglial markers when they were expanded for more than 10 weeks (but unchanged when expanded for <6 weeks; Bhattacharyya *et al*, 2009). In contrast, other studies demonstrated that even at early stages of neural development, fetal DS NPCs are less neurogenic and adopt a more gliocentric progenitor phenotype as revealed by the reduced expression of neuroepithelial and neuronal markers ( *NESTIN*, *PAX6*, *MAP2* and *DCX*) together with the increased expression of several glial markers ( *GFAP*, *S100B*, *OLIG1*, *OLIG2*, *CNPase*, *O4* and *PDGFRA*; Esposito *et al*, 2008; Lu *et al*, 2011, 2012). Considering the key roles of *S100B*, *GFAP* and *VIM* in glial differentiation (Lu *et al*, 2011), the overexpression of these astroglial genes in NPCs derived from Twin-DS-iPSCs leads ultimately to more gliogenic progenitors and the generation of astroglial cells upon differentiation. Moreover, the oligodendroglial shift induced by the overexpression of *OLIG1* and *OLIG2* in Twin-DS-iPSC-derived cells is in accordance with the pivotal role of these HSA21 genes in specifying oligodendrocyte lineage in the central nervous system of vertebrate (Zhou & Anderson, 2002). Collectively, our results suggest that NPCs derived from Twin-DS-iPSCs fail to generate the same number of neurons as those derived from Twin-N-iPSCs due to defects in NPCs rather than in survival of the generated neurons after 4 weeks of differentiation. It is important to note, however, that we cannot exclude that neurons derived from Twin-DS-iPSCs could be more susceptible to apoptosis after long-term culture, reflecting in some extent late occurring neurodegeneration observed in DS patients (Shi *et al*, 2012).

Neurons derived from Twin-DS-iPSCs exhibited not only a reduction of their population but also structural alterations compared to those derived from Twin-N-iPSCs. We found in particular a reduced number and length of neurites from soma of neurons derived from Twin-DS-iPSCs which is consistent with previous morphometric studies on brain of DS patient (Becker *et al*, 1991) and in neurons differentiated from fetal DS NPCs (Bahn *et al*, 2002). In line with this, we present evidence that neurons derived from Twin-DS-iPSCs exhibit a synaptic phenotype similar to that observed in DS patients and in mouse models of DS (Becker *et al*, 1991; Dierssen *et al*, 2003; Chakrabarti *et al*, 2007). Neurons derived from Twin-DS-iPSCs exhibit a reduced density of SYNAPSIN punctae. Considering the key role of the pre-synaptic proteins such as SYNAPSIN, SNAP25 in the regulation of transmitter release, their reduced expression may alter synaptic function in neurons derived from Twin-DS-iPSCs. Moreover, excess inhibitory synapses at the expense of excitatory ones has been proposed to explain the abnormal synaptic plasticity in mouse models of DS (Kleschevnikov *et al*, 2004; Belichenko *et al*, 2007; Chakrabarti *et al*, 2007; Martínez-Cué *et al*, 2013). In the present study, we found a reduced expression of PSD95 in neurons derived from Twin-DS-iPSCs, indicative of a lower proportion of excitatory glutamatergic synapses. In addition, the proportion of inhibitory GABA-ergic synapses stained positive for GAD67 was not substantially altered in neurons derived from Twin-DS-iPSCs. This contrasts with the enhanced immunoreactivity of proteins associated with GABA-ergic synapses reported in several brain regions of mouse model of DS (Belichenko *et al*, 2007; Mazur-Kolecka *et al*, 2012; Martínez-Cué *et al*, 2013). Although a recent study showed no difference in the excitatory post-synaptic properties of neurons derived from normal and DS iPSCs (Shi *et al*, 2012), it will be interesting in the future to investigate the excitatory and inhibitory post-synaptic properties of neurons derived from our iPSCs.

Given that HSA21 contains hundreds of genes, it has been a challenge to identify critical genes responsible for the cognitive impairment in DS patients. Among the candidate genes, *DYRK1A* has received increased interest due to its implication in neurodevelopment in flies, mice and humans (Tejedor *et al*, 1995; Guimera *et al*, 1996; Song *et al*, 1996; Guedj *et al*, 2009; Tejedor & Hämmerle, 2011; Mazur-Kolecka *et al*, 2012). Both loss and gain of function of DYRK1A result in neurodevelopmental defects. *Dyrk1A*^−/−^ null mutant mice show growth delay and die during midgestation whereas mice heterozygous for *Dyrk1A* mutation ( *Dyrk1A*^−/+^) display a brain size 30% smaller than wild type with a region-specific reduction of neurons (Fotaki *et al*, 2002; Benavides-Piccione *et al*, 2005). In humans, DYRK1A haploinsufficiency is associated with microcephaly, growth and mental retardation (Møller *et al*, 2008; Yamamoto *et al*, 2011). Moreover, *Dyrk1A* transgenic mice carrying an extra copy of *Dyrk1A*, exhibit features similar to those observed in DS patients including hippocampal-dependent memory tasks, arrest of neurogenesis and altered synaptic plasticity (Smith *et al*, 1997; Altafaj *et al*, 2001; Ahn *et al*, 2006; Das & Reeves, 2011; Martinez de Lagran *et al*, 2012). The present study provides novel evidence that DYRK1A overexpression is a main contributor to the impaired neurogenesis observed in NPCs and neurons derived from Twin-DS-iPSCs. This was strongly supported by the findings that DYRK1A inhibition by EGCG or shRNA improved proliferation of NPCs derived from Twin-DS-iPSCs. Several additional lines of evidence reinforced the involvement of DYRK1A in the control of cell growth and neurogenesis. DYRK1A has been shown to phosphorylate p53 which leads to the up-regulation of p53 target genes associated with cell cycle regulation and more particularly *p21*^*CIP1*^ in rat and human NPCs as well as in brains from *Dyrk1A* transgenic mice and DS patients. The induction of *p21*^*CIP1*^ impairs G1/G0-S phase transition, inhibiting NPC proliferation (Park *et al*, 2010). In another study, Yabut *et al* demonstrated that *Dyrk1A* overexpression inhibits proliferation and induces premature neuronal differentiation of NPCs (Yabut *et al*, 2010). They showed that *Dyrk1A* overexpression through *in utero* electroporation inhibits neural cell proliferation in the embryonic neocortex. This effect was mediated by the nuclear export and degradation of cyclin D1, a cyclin required for cell proliferation by allowing the entry to the S phase (Yabut *et al*, 2010). More recently, Hämmerle and colleagues showed that *Dyrk1A* promotes cell cycle exit through induction of the expression of the cyclin-dependent kinase inhibitor *p27*^*KIP1*^ in neural precursors, which further binds to and inhibits the cyclin/cyclin-dependent kinase complex that controls G1/S transition (Hämmerle *et al*, 2011). Moreover, Litovchick and colleagues demonstrated that DYRK1A plays an important role in suppression of mammalian cell proliferation. In particular, DYRK1A overexpression promotes quiescence and senescence through DREAM (DP, RB, E2F and MuvB) complex assembly, a complex involved in cell cycle processes (Litovchick *et al*, 2011).

Contrary to the numerous reported roles of *DYRK1A* in the control of cell cycle, very little is known regarding its impact on cell death. For instance, DYRK1A has been shown to prevent the intrinsic apoptotic pathway through the phosphorylation of caspase-9 during retina development (Laguna *et al*, 2008). In contrast, the overexpression of *Dyrk1A* makes rat embryonic hippocampal progenitor cells more susceptible to apoptosis by phosphorylating and activating p53 which leads to the subsequent upregulation of p53 target genes and proteins such as FAS (Park *et al*, 2010). This is of special interest as the protein levels of the pro-apoptotic genes *p53* and *FAS* are increased in the cerebral cortex and the cerebellum of DS patients (de la Monte *et al*, 1998; Seidl *et al*, 1999). In the present study, we provide novel evidence that the increased expression and activity of DYRK1A is responsible for the greater susceptibility to apoptosis of NPCs derived from Twin-DS-iPSCs. Reducing DYRK1A expression and activity to near control values prevented the apoptosis of these cells. It remains to be established whether p53 and FAS are responsible for the increase in apoptosis induced by *DYRK1A* overexpression in NPCs derived from Twin-DS-iPSCs.

Another important observation is that DYRK1A inhibition by EGCG treatment or by shRNA increased the number of neurons derived from Twin-DS-iPSCs. Consistent with a previous study (Canzonetta *et al*, 2008), this effect was associated with an increase of the expression of *REST/NRSF*, a transcription factor that activates genes encoding fundamental functions during neuronal differentiation including neural lineage specification, synapse formation and function (Kuwabara *et al*, 2004; Ballas *et al*, 2005; Abrajano *et al*, 2009). In this regard, the downregulation of *REST/NRSF* in fetal DS NPCs has been shown to lead to the subsequent downregulation of important regulators of cell adhesion and synaptic plasticity (Bahn *et al*, 2002). In agreement with this report, we found a reduced expression of *REST/NRSF* in NPCs and of *SYN1* in neurons derived from Twin-DS-iPSCs. Moreover, recent studies point to an important role of DYRK1A in dendrites tree development, synapse formation and function (Tejedor & Hämmerle, 2011; Martinez de Lagran *et al*, 2012). For instance, single overexpression of *Dyrk1A in vivo* is sufficient to recapitulate the dendritic alterations observed in DS patients (Martinez de Lagran *et al*, 2012). In line with this, we found that DYRK1A inhibition by EGCG or by shRNA promotes the dendritic development of neurons derived from Twin-DS-iPSCs. This effect was associated with a restoration of the expression to near normal levels of *WNT7A*, *WNT7B*, *NOTCH1*, *NOTCH2*, *DLL1* and *HES1*. This is of special interest as these genes are crucial regulators of dendrite morphogenesis and synapse assembly (Ciani & Salinas, 2005; Breunig *et al*, 2007; Ables *et al*, 2011) and could explain at least in part, the defects in dendritic development due to *DYRK1A* overexpression in the present study and recent reports (Guedj *et al*, 2009; Tejedor & Hämmerle, 2011; Martinez de Lagran *et al*, 2012). Of note, when NPCs derived from Twin-DS-iPSCs were treated with EGCG during neuronal differentiation only (and not before, during neural induction), no improvement in the number of neurons was found. However, this delayed targeting of DYRK1A by EGCG promoted dendritic development and the density of the synaptic protein SYNAPSIN in these neurons (supplementary Fig S10). These results further highlight the pleiotropic roles of DYRK1A during the sequential stages of neural induction and neuronal differentiation of iPSCs as well as the potential opportunities for DS therapy through DYRK1A targeting in early and late neurodevelopmental events.

In conclusion, the generation of iPSCs from monozygotic twins discordant for trisomy 21 provides a unique model to study the effects of trisomy 21 of two otherwise genetically identical samples (Dahoun *et al*, 2008). This model recapitulates neurodevelopmental features of DS and provides important clues to the physiopathology of the disease (supplementary Fig S11). Our study further emphasizes DYRK1A as a main contributor of neurogenesis impairment in NPCs and neurons derived from Twin-DS-iPSCs and provides novel insights into the therapeutic potential of DYRK1A targeting in patients with DS. It is important to note, however, that other genes including *OLIG*, *NOTCH* and *WNT* deserve further investigation. Finally, the finding that DYRK1A inhibition by pharmacological and genetic approaches contributes to reverse the abnormal neurogenesis in NPCs and neurons derived from Twin-DS-iPSCs, allows a proof-of-principle for potential screening tests using iPSC technology (Hibaoui & Feki, 2012) and should provide the basis for designing new therapeutic approaches for patients with DS.

## Materials and Methods

### Cell culture and iPSC derivation

Primary fetal skin fibroblasts were isolated from monozygotic twins discordant for trisomy 21 (Twin-N for the normal fibroblasts and Twin-DS for the trisomy 21) and used to establish normal (Twin-N-iPSCs) and Down syndrome (Twin-DS-iPSCs) induced pluripotent stem cells (iPSCs). These samples were obtained post mortem and the study was approved by the ethics committee of the Geneva University Hospital. These iPSCs were generated by transducing the parental fibroblasts (Twin-N and Twin-DS) with polycistronic lentiviral vectors expressing *OCT4*, *SOX2*, *KLF4* and *c-MYC* genes (Takahashi *et al*, 2007; Grad *et al*, 2011). Colonies that appeared were isolated and expanded into lines (see also supplementary Table S1). H1-hESCs (obtained from WiCell Research Institute, Madison, WI, USA) and the generated iPSCs were cultured on irradiated human foreskin fibroblasts (ATCC; CCD 1112Sk, Manassas, VA, USA) that were mitotically inactivated by irradiation at 35 Gy before seeding on a gelatin-coated 6-well plate at 3.5 × 10^5^ cells/plate. IPSC colonies were maintained with daily changes in Knock-out Dulbecco's Minimal Essential Medium (KO DMEM) supplemented with 20% KO serum replacement, 1 mM l-glutamine, 100 μM non-essential amino acids, 100 μM 2-mercaptoethanol, 50 U/ml penicillin and 50 mg/ml streptomycin (all from Gibco, Invitrogen, Basel, Switzerland) and 100 ng/ml human basic fibroblast growth factor (bFGF; Peprotech, London, UK). All iPSC lines were passaged by manual dissection of cell clusters in the presence of 10 μM ROCK-inhibitor Y-27632 (Sigma-Aldrich, Buhs, Switzerland).

### Spontaneous *in vitro* three-germ layer differentiation

Whole iPSC colonies were seeded into ultra low attachment dishes (Costar; Corning Life Sciences, Schiphol-Rijk, The Netherlands) in KO DMEM medium supplemented with 10% new calf serum, 1 mM l-glutamine, 100 μM non-essential amino acids, 100 μM 2-mercaptoethanol, 50 U/ml penicillin and 50 mg/ml streptomycin. Within 24 h, cells aggregated to form embryoid bodies (EBs). After 4 days growing in suspension, these EBs were transferred to gelatin-coated dishes containing the same medium to allow the cells to differentiate for an additional 17 days.

### Neural induction of iPSCs into NPCs and neuronal differentiation into neurons

Differentiation of iPSCs into neural progenitor cells (NPCs) and mature neurons was established as described with minor modifications (Suter *et al*, 2009; Brennand *et al*, 2011). Briefly, the colonies were mechanically detached from the feeder cells, washed and maintained in suspension in ultra-low attachment dishes (Costar, Corning Life Sciences) for 1 week in neural induction medium (NIM) consisting of DMEM-F12, 1% penicillin/streptomycin, N2 supplement (Gibco, Invitrogen) and replaced with NIM supplemented with 10 ng/ml human recombinant bFGF for another 2 weeks. Thereafter, NPCs were further induced to mature into neurons by plating the dissociated aggregates on poly-ornithine/laminin-coated tissue culture plate in neural differentiation medium (NDM) consisting of neurobasal supplemented with B-27 supplement (Gibco, Invitrogen), 10 ng/ml BDNF (R&D Systems, Inc., Minneapolis, MN, USA), 10 ng/ml GDNF (R&D Systems, Inc.), 1 mM dibutyryl-cyclic AMP, 200 nM ascorbic acid (Sigma-Aldrich, Buhs) and 1% penicillin/streptomycin for 4 weeks. Medium was changed every 3 days.

### Teratoma formation from iPSCs

All the procedures involving animals were conducted in accordance with the Swiss Federal Veterinary Office's guidelines, based on the Swiss Federal Law on Animal Welfare. *In vivo* differentiation studies were carried out by teratoma formation. 5 × 10^6^ cells from each iPSCs were harvested and injected intramuscularly into SCID mice. After 8 weeks, the teratomas were excised, fixed in 4% formaldehyde, and embedded in paraffin for hematoxylin and eosin (HE) staining or for immunohistochemistry analysis with horseradish peroxidase system. 4 μm thick teratomas sections from formalin fixed paraffin embedded samples were analyzed by immunohistochemistry using anti-alpha-fetoprotein (NCL-AFPp, Novocastra Laboratories Ltd, Newcastle upon Tyne, UK), anti-alpha-smooth muscle actin (M0851, Dako, Baar, Switzerland) and anti-nestin antibodies (MAB1259, R&D systems, Inc.) with the Ventana Discovery automated staining system (Ventana Medical Systems, Tucson, AZ, USA). Ventana reagents for the entire procedure were used. For both AML and AFP antibodies, no antigen retrieval pretreatment was required. After automatic deparaffinization, slides were incubated 30 min at 37°C with primary antibodies respectively diluted at 1/300 (AML) and 1/750 (AFP) in antibody diluent from Dako (S2022). For nestin antigen retrieval, slides were heated in CC1 cell conditioning solution for 36 min (EDTA antigen retrieval solution pH 8.4) using a standard protocol. Anti-nestin antibodies were used at dilution 1/1000 and also incubated 30 min at 37°C. Detection of primary antibodies was carried out using the secondary universal biotinylated antibodies reagent and the amplified DABMap kit (Ventana Medical Systems), based on conversion of diaminobenzidine to a dye with multimeric horseradish peroxidase (HRP). As negative control, AFP antibodies were replaced by rabbit control immunoglobulins and AML and nestin antibodies were replaced by mouse IgG serotype control.

### Immunocytochemistry

Briefly, iPSCs at different stages of neuronal differentiation were fixed in 4% paraformaldehyde in phosphate buffered saline (PBS) for 30 min, permeabilized with 0.2% Triton X-100 for 30 min, and blocked with 5% bovine serum albumin in PBS for 1 h at room temperature (RT). Cells were incubated with primary antibody overnight at 4°C, washed with PBS and incubated with secondary antibody for 1 h at RT. All secondary antibodies were tested for crossreactivity and nonspecific immunoreactivity. The antibodies used for immunohistochemical staining are listed in supplementary Table S4. The cells were finally mounted in UltraCruz ™ mounting medium containing DAPI for nuclei identification (Santa Cruz Biotechnology, Inc., Santa Cruz, CA, USA). Images of the immunostained cells were captured on a Zeiss Axioskop 2 fluorescence microscope with an Axiocam Color HRc detector (Zeiss, Feldbach, Switzerland). Staining of cells derived from Twin-N-iPSCs and Twin-DS-iPSCs, neurite length and number were analyzed with ImageJ software. *N* values for the immunocytochemical analyses represent at least four independent experiments in triplicate.

### Karyotype analysis

Karyotyping was performed on at least twenty metaphase spreads using the CTG-banding method. IPSCs at 80% confluence were treated with 0.2 μg/ml colcemid (Invitrogen) for 2 h and harvested. Cell pellets were resuspended in pre-warmed hypotonic solution (KCl, 0.075 mol/l) for 10 min at 37°C. Cells were then fixed with freshly prepared, ice-cold methanol-acetic acid solution (3:1 in volume) and mounted by dropping onto slides.

### Bisulfite sequencing analysis

About 2 μg of DNA extracted from the iPSCs and the parental fibroblasts were bisulfite-converted and purified using Epitect Bisulfite Kits (Qiagen, Valencia CA, USA) following the manufacturer's instruction. DNA bisulphite treatment and processing were performed simultaneously for all cell lines. The promoter regions of *OCT4* and *NANOG* were amplified with specific primers previously described (Freberg *et al*, 2007) using JumpStart REDTaq DNA Polymerase (Sigma-Aldrich, Buhs) and KAPA2G Robust DNA Polymerase (Kapabiosystems, Boston, MA, USA). Quantitative methylation percentages were determined by Epitect sequencing (QIAGEN GmbH, Hilden, Germany). The Epitect sequencing service includes sequencing of the PCR products using cycle sequencing (Reactions were cycled in a GeneAmp® PCR System 9700 (Applied Biosystems, Foster City, CA, USA) and purified using DyeEx™ (QIAGEN). Data collection was carried out on a 3730xl DNA Analyzer (Applied Biosystems). It also includes modified analysis of raw data to obtain a percentage of methylation for each CpG dinucleotide. The methylation percentages of these sites were averaged to obtain a single methylation score for each sample.

### Array CGH

Array-Comparative Genomic Hybridization (CGH) was performed using the Human Genome CGH Microarray Kit 44A (Agilent Technologies, Palo Alto, CA, USA) with a resolution of about 150 kb. Briefly, 1 μg of iPSC DNAs were processed according to the manufacturer's protocol. Fluorescence was scanned in a dual-laser scanner (Agilent DNA microarray scanner G2565CA; Agilent Technologies) and the images were extracted and analysed with Agilent Feature Extraction software (v9.5.3.1) and Workbench analysis software (v7.0) respectively. Changes in test DNA copy number at a specific locus are observed as the deviation of the log ratio value from a modal value of 0. The data files have been deposited in the Gene Expression Omnibus (GEO) database under the accession number GSE52251 (http://www.ncbi.nlm.nih.gov/geo/query/acc.cgi?acc=GSE52251).

### RNA extraction, non-quantitative and quantitative real time polymerase chain reaction

Total RNA was extracted from the cell lines, using the QIAGEN RNeasy MiniKit according to the manufacturer's protocol (Invitrogen). RNA integrity and quantity were assessed with an Agilent 2100 bioanalyser (Agilent Technologies, Santa Clara, CA, USA), using RNA 6000 nanochips. 1 μg of total RNAs were reverse transcribed with the Superscript III reverse transcriptase (Invitrogen) according to the manufacturer's protocol. One-twentieth cDNA template was used as template for each PCR reaction. cDNA was real time polymerase chain (PCR) amplified in a 7900HT Sequence Detection Systems (Applied Biosystems) using the Power SYBR Green PCR master mix (Applied Biosystems). Raw threshold-cycle ( *C*_*t*_) values were obtained with the Sequence Detection Systems 2.0 software (Applied Biosystems). Melting curve analysis were automatically performed to monitor production of the appropriate PCR product. Relative quantities (RQs) were calculated with the formula RQ = E− *C*_*t*_, using efficiencies calculated for each run with the Data Analysis for Real-Time PCR (DART-PCR) algorithm, as described (Peirson *et al*, 2003). A mean quantity was calculated from triplicate PCR reactions for each sample, and this quantity was normalized against that of the housekeeping genes ( *GusB* and *eEF1*). Each PCR reaction was performed at least in triplicate with negative controls and the mean quantities were calculated from them. For non-quantitative PCR, reactions were performed in a Biometra thermocycler (Göttingen, Germany), with RedTaq polymerase mix (Sigma-Aldrich, St. Louis, MO, USA), 250 nM primers and 1 μl of cDNA. The primer sequences used for quantitative and non quantitative RT-PCR are listed in supplementary Table S4.

### Gene expression analysis by mRNA sequencing

mRNA-Seq libraries were prepared from 500 ng of total RNA using the Illumina TruSeq™ RNA Sample Preparation kit (S-930-2001, Illumina, Inc., San Diego, CA, USA), following the manufacturer's instructions. Libraries were sequenced on the Illumina HiSeq 2000 instrument (Illumina, Inc.) to generate 100 bp paired-end reads. Those reads were mapped against the genome (hg 19) using the default parameters of the Burrows-Wheeler Aligner (BWA; Li & Durbin, 2009). An expression signal was detected in at least one sample for 20'456 genes (comprising also non-coding RNA). For each gene, the level of expression was determined by calculating the exon coverage (custom pipeline) and normalizing in Reads per Kilobase per Million (RPKM). Twin-DS-iPSC and Twin-N-iPSC samples were sequenced in three and four biological replicates, respectively, starting from different RNA preparations. Principal component analyses (PCA) and clustering analyses were performed using R (version 2.14.0, http://www.R-project.org/). Differential expression between the trisomic and the normal replicates was assessed using the default parameters of EdgeR (version 2.4.6; Robinson *et al*, 2010) and DESeq (version 1.6.1; Anders & Huber, 2010) programs in R (version 2.14.0). Only the genes with more than 1 read per million in at least three replicates were conserved for this analysis. Bonferroni correction was applied to adjust for multiple testing. A gene was considered differentially expressed if the Bonferroni-corrected *P*-value was lower than 0.01 with both methods. Analysis of the functional annotations associated with the differentially expressed genes was performed using Database for Annotation, Visualization and Integrated Discovery (Huang *et al*, 2008, 2009). REVIGO interactive graph of the top biological processes was performed as previously described (Supek *et al*, 2011). The data files have been deposited in the Gene Expression Omnibus (GEO) database under the accession number GSE52251 (http://www.ncbi.nlm.nih.gov/geo/query/acc.cgi?acc=GSE52251).

### Cell death assays

Caspase-3 activity was determined in NPC homogenates as previously described (Hibaoui *et al*, 2009). Caspase–3 activity was measured by monitoring the proteolytic cleavage of the fluorogenic substrate *N*–acetyl-Asp-Glu-Val-Asp-7–amino–4–methylcoumarin (Ac-DEVD–AMC; Biomol, Anawa Trading, Zürich, Switzerland) for 30 min at 30°C using a FLUOstar OPTIMA fluorimeter (BMG Labtech, Champigny sur Marne, France; excitation 380 nm, emission 460 nm). The caspase–3 inhibitor *N*–acetyl-Asp-Glu-Val-Asp-aldehyde (Ac–DEVD–CHO; Biomol) was used at 10 μM as a control. Caspase-3 activity was expressed as AUF per minute 100 μg per protein (AUF for arbitrary units of fluorescence). Protein content was determined using the Bradford method (BioRad, Reinach, Switzerland). Nuclear morphology of NPCs was studied by using the cell permeable DNA-binding dye Hoechst 33342 (Invitrogen). NPCs with homogeneously stained, regularly and roundly shaped nuclei were considered to be normal NPCs whereas NPCs that exhibited reduced nuclear size, chromatin condensation, intense fluorescence, and nuclear fragmentation were considered as apoptotic. NPCs were washed with phosphate-buffered saline (PBS), fixed with 4% paraformaldehyde and stained with 1 μg/ml of Hoechst 33342 for 5 min at room temperature. Nuclear morphological changes were visualized using Zeiss Axioskop 2 fluorescence microscope with an Axiocam Color HRc detector (Zeiss). Nuclei from several randomly chosen areas were counted and the number of apoptotic nuclei was expressed as percentage of the total number of nuclei.

### DYRK1A kinase assay

NPCs were lysed by up-and-down pipetting with a lysis buffer (50 mM Tris-HCl pH7.5, 150 mM NaCl, 0.5% NP-40, 15% glycerol, 1 mM EDTA, 1 mM NaF, 1 mM Na_3_VO_4_, 10 μg/ml aprotinin, 10 μg/ml pepstatin, 10 μg/ml leupeptin, 1 μl PMSF). After centrifugation (21 000 *g*, 4°C, 10 min), the supernatant was stored at −80°C. DYRK1A immunoprecipitation was performed using a monoclonal anti-DYRK1A (clone 7D10; Abnova, Taipei City, Taiwan) as previously described with slight modifications (Lilienthal *et al*, 2010). *In vitro* DYRK1A kinase activity was measured by incubating the immunoprecipated DYRK1A with the substrate peptide DYRKtide (Catalog#62698; Anaspec, Fremont, CA, USA) in a kinase buffer (25 mM Hepes pH 7.0, 5 mM MgCl_2_ and 0.5 mM dithiothreitol) containing 40 μM ATP for 3 h at 37°C. DYRK1A kinase activity was measured by quantifying the amount of remaining ATP in the reaction using kinase-Glo luminescent reagent (Promega, Dübendorf, Switzerland). Luminescence was monitored in a plate reader luminometer (Fluostar Optima; BMG Labtechnologies, Offenburg, Germany).

### DYRK1A knockdown assay and DYRK1A inhibition with epigallocatechine gallate

Knockdown of DYRK1A was achieved by means of lentiviral vector-mediated short hairpin RNA (shRNA), using validated commercial lentiviral particles for the DYRK1A sequence (Sigma-Aldrich, Buhs). For viral transduction, Twin-DS-iPSCs were plated on matrigel-coated dishes and infected at a multiplicity of infection of 30 in the presence of 10 μg/ml polybrene. As controls, Twin-N-iPSCs and Twin-DS-iPSCs were also infected with control shRNA. Knockdown efficiencies of DYRK1A targeted shRNA were evaluated by real-time RT-PCR and by Western blotting analysis. Moreover, to test the effect of pharmacological DYRK1A inhibition on neurogenesis, Twin-DS-iPSCs were treated with the selective DYRK1A inhibitor epigallocatechine gallate EGCG (Sigma-Aldrich, Buhs) at a concentration of 10 μM in NIM and NDM with medium changes every 2 days. All comparative differentiation experiments were carried out with all experimental lines and controls in parallel.

**The paper explained**

**Problem**

Down syndrome (DS) caused by a trisomy of chromosome 21 (HSA21), is the most common genetic developmental disorder, with an incidence of one in 800 live births. DS individuals show intellectual impairment and several other abnormalities for the majority of which the pathogenetic mechanisms remain unknown. A major challenge has been to recapitulate the disease features and to understand the molecular mechanisms by which the extra copy of HSA21 genes leads to the abnormalities observed in DS patients. Recent studies have been successful in generating disease-specific induced pluripotent stem cells (iPSCs) from a variety of neurodevelopmental and neurodegenerative disorders, providing useful *in vitro* models.

**Results**

We report the generation and characterization of iPSCs derived from monozygotic twins discordant for trisomy 21 in order to eliminate the effects of the variability of the genomic background. This provides a unique model to study the effect of the supernumerary HSA21 since the rest of the genome is identical between the two twins. DS iPSCs recapitulate the neurodevelopmental features of DS. First, the alterations observed by genetic analysis at the iPSC level and at first approximation in early development illustrate the developmental disease transcriptional signature of DS. Then, *in vivo* differentiation of DS iPSCs revealed an abnormal teratoma formation in NOD-SCID mice. *In vitro*, we found a reduced neurogenesis together with a greater astroglial and oligodendroglial cell population upon neural induction into neural progenitor cells (NPCs) and upon neuronal differentiation of DS iPSCs. This effect was associated with a proliferation deficit and increased apoptosis of NPCs derived from DS iPSCs. In addition, neurons derived from DS iPSCs showed reduced dendritic development and expression of synaptic proteins. Importantly, we provide novel evidence that *DYRK1A* on HSA21 likely contributes to these defects as its inhibition by pharmacological means or by short hairpin RNA silencing corrected these defects. We found that this correction improved proliferation, decreased apoptosis of NPCs and increased the number of neurons derived from DS iPSCs. Also, *DYRK1A* correction was associated with the restoration of the expression to near normal levels of crucial regulators of neurogenesis and dendritic development including *REST/NRSF*, *WNT* and *NOTCH* signalling.

**Impact**

The generation of iPSCs from monozygotic twins discordant for trisomy 21 is an innovative way to study DS neurodevelopment as it offers an unprecedented opportunity to study early embryonic development and enables the investigation of the detailed pathogenetic mechanisms by which the extra copy of HSA21 leads to DS phenotypes. The finding that DYRK1A inhibition by pharmacological and genetic approaches contributes to reversal of the abnormal neurogenesis in NPCs and neurons derived from DS iPSCs, allows a proof-of-principle for potential screening tests using iPSC technology and should provide the basis for designing new therapeutic approaches for DS patients.

### Western blotting

NPCs were solubilized at 4°C in 100 μl of lysis buffer and centrifuged to remove debris. Protein were resolved in 7.5% SDS-polyacrylamide gels and transferred to nitrocellulose membranes. Incubation with anti-DYRK1A antibody was carried out overnight at 4°C using dilutions of 1:1000 (clone 7D10; Abnova). After incubation with HRP-conjugated secondary antibodies during 1 h, membranes were developed by enhanced chemiluminescence reagents (ECL reagent; Amersham Biosciences) according to the manufacturer's instructions. Signals were quantified and normalized to α-tubulin (clone DM1A T9026; Sigma, Buhs) and expressed as arbitrary units.

### Data analysis

The data were analyzed and the graphs were constructed using the GraphPad Prism software (GraphPad, San Diego, CA, USA). Data are shown as mean ± s.e.m. Statistical analysis among groups were evaluated by one-way analysis of variance (ANOVA) followed by Tukey's post hoc test, and comparisons between two groups by Student's *t*–test. *P* values of <0.05 were considered significant.

## Notes

**Supplementary information** for is this article available online: http://www.embomm.embopress.org
