# Supplementary material for: Modelling and rescuing neurodevelopmental defect of Down syndrome using induced pluripotent stem cells from monozygotic twins discordant for trisomy 21
Source: EMBO Mol Med. 2013 Dec 27;6(2):259–77. doi: 10.1002/emmm.201302848 (PMC3927959; doi:10.1002/emmm.201302848)
Supplement: Supplementary file 1 [file emmm0006-0259-sd1.pdf]

Modelling and rescuing neurodevelopmental defect of Down syndrome using induced pluripotent stem cells derived from monozygotic twins discordant for trisomy 21

Youssef Hibaoui, Iwona Grad, Audrey Letourneau, M. Reza Sailani, Sophie Dahoun, Federico A. Santoni, Stefania Gimelli, Michel Guipponi, Marie Françoise Pelte, Frédérique Béna, Stylianos E. Antonarakis and Anis Feki

SUPPORTING INFORMATION, TABLE OF CONTENT

This section includes 5 Tables and 11 Figures.

Supporting Information Table 1. Summary of iPSC lines generated and used for the study…………………………………...page 3

Supporting Information Table 2. List of the differentially expressed genes between Twin-N-iPSCs and Twin-DS-iPSCs (see the EXCEL FILE and the R OBJECT FILE used to generate Fig 2E)……………………………………………………………………….page 4

Supporting Information Table 3. Complete gene ontology (GO) categories of biological processes, cellular components, molecular functions and pathways in which significant differentially expressed genes were grouped……………………………………………..page 5

Supporting Information Table 4. List of the antibodies used for immunocytochemistry……………………………………………………………......page 10

Supporting Information Table 5. List of primers used in semi-quantitative and quantitative RT-PCR…………………………………………………………………...……page 11

Supporting Information Fig 1. Additional controls validating the successful reprogramming and generation of Twin-N-iPSCs and Twin-DS-iPSCs…………………………………page 12

Supporting Information Fig 2. Heat map and principal component analysis (PCA) plot and heat map of the normalized expression values in Twin-N-iPSCs and Twin-DS-iPSCs when analyzing the HSA21 genes only……………………………………………………………………………...page 13

Supporting Information Fig 3. Heat map of the normalized gene expression values in Twin-DS-iPSCs and Twin-N-iPSCs for the 96 downregulated genes involved in brain-related functions……………………………………………………………………………...page 15

Supporting Information Fig 4. Additional controls validating the absence of ectodermal structures observed in Twin-DS-iPSC-derived teratomas…..…….……………..…….page 17

Supporting Information Fig 5. Proportion of each cell type in neurospheres derived from Twin-N-iPSCs and Twin-DS-iPSCs upon neural induction…………………………………………………………………………...…page 18

Supporting Information Fig 6. Proliferative properties of each cell type in neurospheres derived from Twin-N-iPSCs and Twin-DS-iPSCs upon neural induction…………………………………………………………………………...page 19

Supporting Information Fig 7. Apoptotic properties of each cell type in neurospheres derived from Twin-N-iPSCs and Twin-DS-iPSCs upon neural induction…………………………………………………………………………...page 20

Supporting Information Fig 8. Knockdown efficiencies of DYRK1A shRNA in NPCs derived from Twin-DS-iPSCs…………………………………………………………..…page 21

Supporting Information Fig 9. REVIGO interactive graph of the top biological processes associated with the 580 downregulated genes…………………………………….…….page 22

Supporting Information Fig 10. Additional effects of a delayed targeting of DYRK1A through EGCG treatment on neurons derived from Twin-DS-iPSCs…………………………………………………………………………....page 23

Supporting Infomation Fig 11. Proposed mechanisms responsible for the abnormal neuronal differentiation of Twin-DS-iPSCs…………………………………………………....page 24
