# Supplementary material for: Modelling and rescuing neurodevelopmental defect of Down syndrome using induced pluripotent stem cells from monozygotic twins discordant for trisomy 21
Source: EMBO Mol Med. 2013 Dec 27;6(2):259–77. doi: 10.1002/emmm.201302848 (PMC3927959; doi:10.1002/emmm.201302848)
Supplement: Supplementary file 2 [file emmm0006-0259-sd2.pdf]

	Karyotype	Transgene silencing	Pluripotency markers	*In vitro* differentiation	Teratoma assay
**Twin-N-iPSCs**					
Line 3	46, XX	Passed	Passed	Passed	Passed
Line 7	46, XX	Passed	Passed	Passed	Passed
**Twin-DS-iPSCs**					
Line 4	47, XX +21	Passed	Passed	Passed	Passed
Line 6	47, XX +21	Passed	Passed	Passed	Passed

Supporting Information Table 1. Summary of iPSC lines generated
Normal (Twin-N) and Down syndrome (Twin-DS) fetal fibroblasts were isolated from monozygotic twins discordant for trisomy 21 (Dahoun et al, 2008) and used to establish Twin-N-iPSCs and Twin-DS-iPSCs using lentiviral vectors expressing *OCT4*, *SOX2*, *KLF4* and *c-MYC* genes as previously described (Takahashi et al, 2007; Grad I et al, 2011). Several iPSC lines have been generated from the parental Twin-N and Twin-DS fibroblasts: 8 Twin-N-iPSC and 12 Twin-DS-iPSC lines. IPSC lines that we did not succeed to expand or those that exhibited chromosomal aberrations have been excluded from the study. Two iPSC lines for each twin have been characterized for *in vivo* differentiation in a teratoma formation assay and *in vitro* differentiation into NPCs and neurons.
