# Supplementary material for: Modelling and rescuing neurodevelopmental defect of Down syndrome using induced pluripotent stem cells from monozygotic twins discordant for trisomy 21
Source: EMBO Mol Med. 2013 Dec 27;6(2):259–77. doi: 10.1002/emmm.201302848 (PMC3927959; doi:10.1002/emmm.201302848)
Supplement: Supplementary file 3 [file emmm0006-0259-sd3.pdf]

Supporting Information Table 2. List of the differentially expressed genes between Twin-N-iPSCs and Twin-DS-iPSCs (see the EXCEL FILE and the R OBJECT FILE used to generate Fig 2).

1204 genes were differentially expressed between Twin-N-iPSCs and Twin-DS-iPSCs (Bonferroni-corrected p-value<0.01): 580 genes were found downregulated and 624 genes upregulated in Twin-DS-iPSCs in comparison with Twin-N-iPSCs. The tables show the gene coordinates (chromosome, start, end), the cDNA length, the official gene symbol, the strand, the log2 fold change between Twin-DS-iPSCs and Twin-N-iPSCs (the smallest fold change between DESeq and EdgeR is reported), the nominal p-values and the Bonferroni-corrected p-values given by EdgeR and DESeq.
