# Supplementary material for: Modelling and rescuing neurodevelopmental defect of Down syndrome using induced pluripotent stem cells from monozygotic twins discordant for trisomy 21
Source: EMBO Mol Med. 2013 Dec 27;6(2):259–77. doi: 10.1002/emmm.201302848 (PMC3927959; doi:10.1002/emmm.201302848)
Supplement: Supplementary file 6 [file emmm0006-0259-sd6.pdf]

Category	Term	Count	%	*P*-value	Benjamini
GO categories of biological processes associated with the 580 downregulated genes:					
GO:0007275	multicellular organismal development	189	33,6	1,10E-29	2,40E-26
GO:0048731	system development	165	29,3	1,60E-28	1,70E-25
GO:0032502	developmental process	195	34,6	3,20E-27	2,30E-24
GO:0048856	anatomical structure development	170	30,2	6,20E-27	3,40E-24
GO:0009653	anatomical structure morphogenesis	103	18,3	8,20E-23	3,60E-20
GO:0007399	nervous system development	95	16,9	2,30E-21	8,20E-19
GO:0048513	organ development	125	22,2	3,40E-21	1,00E-18
GO:0032501	multicellular organismal process	222	39,4	3,40E-21	9,30E-19
GO:0030154	cell differentiation	117	20,8	1,70E-19	4,20E-17
GO:0048869	cellular developmental process	118	21	1,50E-18	3,30E-16
GO:0007156	homophilic cell adhesion	30	5,3	1,80E-17	3,60E-15
GO:0022610	biological adhesion	68	12,1	1,90E-17	3,40E-15
GO:0007155	cell adhesion	67	11,9	6,60E-17	1,90E-14
GO:0016337	cell-cell adhesion	41	7,3	9,70E-17	1,70E-14
GO:0009887	organ morphogenesis	54	9,6	1,50E-13	2,20E-11
GO:0007389	pattern specification process	36	6,4	2,20E-13	3,00E-11
GO:0009888	tissue development	59	10,5	2,40E-13	3,10E-11
GO:0001501	skeletal system development	39	6,9	4,20E-13	5,10E-11
GO:0009790	embryonic development	53	9,4	7,50E-13	8,50E-11
GO:0048598	embryonic morphogenesis	37	6,6	2,90E-12	3,10E-10
GO:0022008	neurogenesis	52	9,2	2,20E-11	2,30E-09
GO:0043009	chordate embryonic development	37	6,6	2,60E-11	2,60E-09
GO:0009792	embryonic development ending in birth or egg hatching	37	6,6	3,40E-11	3,20E-09
GO:0048699	generation of neurons	48	8,5	1,90E-10	1,80E-08
GO:0006357	regulation of transcription from RNA polymerase II promoter	56	9,9	2,70E-10	2,40E-08
GO:0050793	regulation of developmental process	52	9,2	1,30E-09	1,10E-07
GO:0051239	regulation of multicellular organismal process	63	11,2	3,80E-09	3,10E-07
GO:0030182	neuron differentiation	39	6,9	5,10E-09	3,90E-07
GO:0045893	positive regulation of transcription, DNA-dependent	41	7,3	5,20E-09	3,90E-07
GO:0060429	epithelium development	27	4,8	5,60E-09	4,00E-07
GO:0003002	regionalization	25	4,4	6,40E-09	4,50E-07
GO:0010628	positive regulation of gene expression	46	8,2	6,50E-09	4,40E-07
GO:0051254	positive regulation of RNA metabolic process	41	7,3	6,60E-09	4,30E-07
GO:0007417	central nervous system development	38	6,7	7,40E-09	4,70E-07
GO:0045944	positive regulation of transcription from RNA polymerase II promoter	35	6,2	8,60E-09	5,30E-07
GO:0048568	embryonic organ development	23	4,1	1,10E-08	6,80E-07
GO:0035295	tube development	26	4,6	1,30E-08	7,60E-07
GO:0048562	embryonic organ morphogenesis	20	3,6	1,80E-08	1,10E-06
GO:0051216	cartilage development	15	2,7	3,80E-08	2,10E-06
GO:0045595	regulation of cell differentiation	40	7,1	3,80E-08	2,10E-06
GO:0048518	positive regulation of biological process	104	18,5	4,30E-08	2,30E-06
GO:0035239	tube morphogenesis	19	3,4	5,00E-08	2,60E-06
GO:0045941	positive regulation of transcription	43	7,6	6,50E-08	3,30E-06
GO:0048468	cell development	46	8,2	1,40E-07	6,90E-06
GO:0000122	negative regulation of transcription from RNA polymerase II promoter	27	4,8	1,50E-07	7,00E-06
GO:0048522	positive regulation of cellular process	94	16,7	3,20E-07	1,50E-05
GO:0010557	positive regulation of macromolecule biosynthetic process	45	8	5,60E-07	2,60E-05
GO:0042471	ear morphogenesis	13	2,3	5,60E-07	2,60E-05
GO:0001944	vasculature development	25	4,4	6,60E-07	2,90E-05
GO:0006355	regulation of transcription, DNA-dependent	90	16	7,20E-07	3,10E-05
GO:0007422	peripheral nervous system development	10	1,8	8,90E-07	3,80E-05

Category	Term	Count	%	*P*-value	Benjamini
GO categories of biological processes associated with the 580 downregulated genes (continued):					
GO:0045165	cell fate commitment	18	3,2	1,00E-06	4,20E-05
GO:0045935	positive regulation of nucleobase, nucleoside, nucleotide and nucleic acid metabolic process	43	7,6	1,00E-06	4,10E-05
GO:0048646	anatomical structure formation involved in morphogenesis	30	5,3	1,00E-06	4,20E-05
GO:0045892	negative regulation of transcription, DNA-dependent	30	5,3	1,30E-06	5,20E-05
GO:0010468	regulation of gene expression	128	22,7	1,40E-06	5,30E-05
GO:0016481	negative regulation of transcription	35	6,2	1,40E-06	5,30E-05
GO:0000904	cell morphogenesis involved in differentiation	24	4,3	1,40E-06	5,40E-05
GO:0001568	blood vessel development	24	4,3	1,60E-06	5,70E-05
GO:0010629	negative regulation of gene expression	37	6,6	1,60E-06	5,80E-05
GO:0007409	axonogenesis	21	3,7	1,60E-06	5,80E-05
GO:0051253	negative regulation of RNA metabolic process	30	5,3	1,80E-06	6,40E-05
GO:0051252	regulation of RNA metabolic process	90	16	1,90E-06	6,40E-05
GO:0001763	morphogenesis of a branching structure	13	2,3	2,00E-06	6,90E-05
GO:0051173	positive regulation of nitrogen compound metabolic process	43	7,6	2,30E-06	7,60E-05
GO:0007420	brain development	26	4,6	2,40E-06	8,00E-05
GO:0045449	regulation of transcription	118	21	2,50E-06	8,00E-05
GO:0010604	positive regulation of macromolecule metabolic process	52	9,2	2,60E-06	8,40E-05
GO:0009891	positive regulation of biosynthetic process	45	8	2,80E-06	8,80E-05
GO:0019219	regulation of nucleobase, nucleoside, nucleotide and nucleic acid metabolic process	125	22,2	3,10E-06	9,70E-05
GO:0031328	positive regulation of cellular biosynthetic process	44	7,8	4,60E-06	1,40E-04
GO:0051171	regulation of nitrogen compound metabolic process	125	22,2	4,90E-06	1,50E-04
GO:0043583	ear development	14	2,5	5,40E-06	1,60E-04
GO:0048667	cell morphogenesis involved in neuron differentiation	21	3,7	5,60E-06	1,60E-04
GO:0051960	regulation of nervous system development	20	3,6	5,90E-06	1,70E-04
GO:0042472	inner ear morphogenesis	11	2	5,90E-06	1,70E-04
GO:0045934	negative regulation of nucleobase, nucleoside, nucleotide and nucleic acid metabolic process	36	6,4	6,00E-06	1,70E-04
GO:0065007	biological regulation	272	48,3	6,10E-06	1,70E-04
GO:0048514	blood vessel morphogenesis	21	3,7	6,40E-06	1,80E-04
GO:0048812	neuron projection morphogenesis	21	3,7	7,40E-06	2,00E-04
GO:0010810	regulation of cell-substrate adhesion	10	1,8	7,90E-06	2,10E-04
GO:0006928	cell motion	34	6	8,00E-06	2,10E-04
GO:0051172	negative regulation of nitrogen compound metabolic process	36	6,4	8,10E-06	2,10E-04
GO:0045785	positive regulation of cell adhesion	11	2	1,10E-05	2,90E-04
GO:0000902	cell morphogenesis	28	5	1,10E-05	2,90E-04
GO:0010811	positive regulation of cell-substrate adhesion	8	1,4	1,50E-05	3,80E-04
GO:0030155	regulation of cell adhesion	16	2,8	1,70E-05	4,20E-04
GO:0030855	epithelial cell differentiation	16	2,8	1,70E-05	4,20E-04
GO:0031327	negative regulation of cellular biosynthetic process	37	6,6	1,80E-05	4,40E-04
GO:0048858	cell projection morphogenesis	22	3,9	1,80E-05	4,40E-04
GO:0048706	embryonic skeletal system development	12	2,1	1,90E-05	4,60E-04
GO:0031326	regulation of cellular biosynthetic process	126	22,4	1,90E-05	4,60E-04
GO:0009893	positive regulation of metabolic process	52	9,2	1,90E-05	4,60E-04
GO:0007411	axon guidance	14	2,5	2,00E-05	4,70E-04
GO:0060348	bone development	15	2,7	2,10E-05	4,80E-04
GO:0050789	regulation of biological process	258	45,8	2,10E-05	4,80E-04
GO:0009952	anterior/posterior pattern formation	16	2,8	2,20E-05	5,00E-04
GO:0048754	branching morphogenesis of a tube	11	2	2,30E-05	5,20E-04
GO:0048839	inner ear development	12	2,1	2,40E-05	5,40E-04
GO:0010558	negative regulation of macromolecule biosynthetic process	36	6,4	2,40E-05	5,40E-04
GO:0031325	positive regulation of cellular metabolic process	50	8,9	2,50E-05	5,40E-04

Category	Term	Count	%	*P*-value	Benjamini
GO categories of biological processes associated with the 580 downregulated genes (continued):					
GO:0060255	regulation of macromolecule metabolic process	136	24,2	2,50E-05	5,50E-04
GO:0009889	regulation of biosynthetic process	126	22,4	2,60E-05	5,60E-04
GO:0019222	regulation of metabolic process	148	26,3	2,60E-05	5,60E-04
GO:0009890	negative regulation of biosynthetic process	37	6,6	2,70E-05	5,70E-04
GO:0007507	heart development	20	3,6	2,90E-05	6,10E-04
GO:0032989	cellular component morphogenesis	29	5,2	3,00E-05	6,10E-04
GO:0048705	skeletal system morphogenesis	14	2,5	3,30E-05	6,70E-04
GO:0048729	tissue morphogenesis	18	3,2	3,30E-05	6,70E-04
GO:0032990	cell part morphogenesis	22	3,9	3,50E-05	6,90E-04
GO:0021915	neural tube development	11	2	3,50E-05	6,90E-04
GO:0048666	neuron development	26	4,6	3,80E-05	7,50E-04
GO:0001503	ossification	14	2,5	4,40E-05	8,50E-04
GO:0050767	regulation of neurogenesis	17	3	4,40E-05	8,40E-04
GO:0048523	negative regulation of cellular process	79	14	4,40E-05	8,40E-04
GO:0014032	neural crest cell differentiation	8	1,4	4,80E-05	9,00E-04
GO:0014033	neural crest cell development	8	1,4	4,80E-05	9,00E-04
GO:0048704	embryonic skeletal system morphogenesis	10	1,8	4,80E-05	9,10E-04
GO:0045664	regulation of neuron differentiation	15	2,7	5,00E-05	9,30E-04
GO:0060284	regulation of cell development	19	3,4	5,20E-05	9,50E-04
GO:0002009	morphogenesis of an epithelium	13	2,3	5,30E-05	9,60E-04
GO:0050794	regulation of cellular process	247	43,9	5,70E-05	1,00E-03
GO:0010556	regulation of macromolecule biosynthetic process	119	21,1	8,00E-05	1,40E-03
GO:0031175	neuron projection development	21	3,7	1,00E-04	1,90E-03
GO:0030278	regulation of ossification	11	2	1,20E-04	2,00E-03
GO:0051093	negative regulation of developmental process	21	3,7	1,20E-04	2,10E-03
GO:0048762	mesenchymal cell development	9	1,6	1,30E-04	2,30E-03
GO:0014031	mesenchymal cell differentiation	9	1,6	1,30E-04	2,30E-03
GO:0060485	mesenchyme development	9	1,6	1,50E-04	2,60E-03
GO:0080090	regulation of primary metabolic process	133	23,6	1,50E-04	2,60E-03
GO:0001525	angiogenesis	15	2,7	1,60E-04	2,70E-03
GO:0031323	regulation of cellular metabolic process	138	24,5	2,10E-04	3,50E-03
GO:0030900	forebrain development	15	2,7	2,10E-04	3,50E-03
GO:0007423	sensory organ development	19	3,4	2,10E-04	3,50E-03
GO:0010605	negative regulation of macromolecule metabolic process	41	7,3	2,30E-04	3,80E-03
GO:0001843	neural tube closure	7	1,2	2,40E-04	3,90E-03
GO:0060606	tube closure	7	1,2	2,40E-04	3,90E-03
GO:0042127	regulation of cell proliferation	43	7,6	2,40E-04	4,00E-03
GO:0016477	cell migration	21	3,7	2,90E-04	4,60E-03
GO:0031324	negative regulation of cellular metabolic process	40	7,1	3,10E-04	4,90E-03
GO:0045596	negative regulation of cell differentiation	18	3,2	3,10E-04	4,90E-03
GO:0051094	positive regulation of developmental process	21	3,7	3,10E-04	4,90E-03
GO:0010646	regulation of cell communication	52	9,2	3,80E-04	6,00E-03
GO:0009892	negative regulation of metabolic process	42	7,5	4,00E-04	6,20E-03
GO:0014020	primary neural tube formation	7	1,2	4,10E-04	6,30E-03
GO:0009953	dorsal/ventral pattern formation	9	1,6	4,20E-04	6,50E-03
GO:0007157	heterophilic cell adhesion	6	1,1	4,30E-04	6,40E-03
GO:0030111	regulation of Wnt receptor signaling pathway	8	1,4	4,30E-04	6,40E-03
GO:0048519	negative regulation of biological process	80	14,2	4,40E-04	6,60E-03
GO:0001755	neural crest cell migration	6	1,1	5,30E-04	7,90E-03

Category	Term	Count	%	*P*-value	Benjamini
GO categories of cellular components associated with the 580 downregulated genes:					
GO:0044421	extracellular region part	60	10,7	5,70E-08	1,70E-05
GO:0005578	proteinaceous extracellular matrix	28	5	9,40E-07	1,40E-04
GO:0031012	extracellular matrix	29	5,2	1,30E-06	1,30E-04
GO:0044420	extracellular matrix part	15	2,7	8,60E-06	6,60E-04
GO:0005576	extracellular region	92	16,3	1,50E-05	9,00E-04
GO:0005886	plasma membrane	149	26,5	4,90E-05	2,50E-03
GO:0005581	collagen	8	1,4	6,00E-05	2,60E-03
GO categories of molecular components associated with the 580 downregulated genes:					
GO:0003700	transcription factor activity	65	11,5	4,10E-09	2,30E-06
GO:0043565	sequence-specific DNA binding	45	8	8,70E-08	2,50E-05
GO:0005509	calcium ion binding	57	10,1	5,10E-07	9,60E-05
GO:0030528	transcription regulator activity	80	14,2	1,10E-06	1,50E-04
GO:0005488	binding	416	73,9	5,60E-06	6,30E-04
GO:0003677	DNA binding	104	18,5	4,10E-05	3,80E-03
GO:0005515	protein binding	290	51,5	6,00E-05	4,80E-03
GO categories of pathways associated with the 580 downregulated genes:					
hsa05200:	Pathways in cancer	25	4,4	3,40E-06	3,70E-04
hsa04310:	Wnt signaling pathway	15	2,7	3,60E-05	2,00E-03
hsa04360:	Axon guidance	11	0,2	2,00E-03	5,20E-02
P00012:	Cadherin signaling pathway	35	6,2	7,10E-17	6,60E-15
P00057:	Wnt signaling pathway	41	7,3	9,50E-11	2,80E-09

Category	Term	Count	%	*P*-value	Benjamini
GO categories of biological processes associated with the 624 upregulated genes:					
GO:0051252	regulation of RNA metabolic process	106	2,1	2,40E-09	5,60E-06
GO:0006355	regulation of transcription, DNA-dependent	104	2,1	3,10E-09	3,60E-06
GO:0006350	transcription	117	2,4	4,60E-09	3,60E-06
GO:0009058	biosynthetic process	169	3,4	4,20E-08	2,40E-05
GO:0044249	cellular biosynthetic process	165	3,3	5,00E-08	2,30E-05
GO:0009059	macromolecule biosynthetic process	140	2,8	1,60E-07	6,30E-05
GO:0045449	regulation of transcription	131	2,6	1,80E-07	5,90E-05
GO:0034645	cellular macromolecule biosynthetic process	139	2,8	1,80E-07	5,30E-05
GO:0051171	regulation of nitrogen compound metabolic process	139	2,8	3,30E-07	8,60E-05
GO:0019219	regulation of nucleobase, nucleoside, nucleotide and nucleic acid metabolic process	138	2,8	3,50E-07	8,00E-05
GO:0010468	regulation of gene expression	138	2,8	8,20E-07	1,70E-04
GO:0010556	regulation of macromolecule biosynthetic process	136	2,7	1,40E-06	2,70E-04
GO:0080090	regulation of primary metabolic process	153	3,1	1,50E-06	2,70E-04
GO:0031326	regulation of cellular biosynthetic process	140	2,8	1,70E-06	2,80E-04
GO:0009889	regulation of biosynthetic process	140	2,8	2,50E-06	3,90E-04
GO:0060255	regulation of macromolecule metabolic process	149	3	6,20E-06	8,90E-04
GO:0031323	regulation of cellular metabolic process	156	3,1	7,80E-06	1,10E-03
GO:0010467	gene expression	138	2,8	1,20E-05	1,60E-03
GO:0019222	regulation of metabolic process	158	3,2	4,00E-05	4,90E-03
GO:0006807	nitrogen compound metabolic process	162	3,3	7,80E-05	9,00E-03
GO categories of molecular components associated with the 624 upregulated genes:					
GO:0008270	zinc ion binding	124	2,5	3,40E-09	2,30E-06
GO:0046914	transition metal ion binding	137	2,8	9,40E-08	3,10E-05
GO:0046872	metal ion binding	184	3,7	3,10E-07	7,00E-05
GO:0043169	cation binding	185	3,7	3,80E-07	6,40E-05
GO:0043167	ion binding	187	3,8	4,10E-07	5,60E-05
GO:0003677	DNA binding	113	2,3	4,30E-06	4,80E-04

Supporting Information Table 3. Complete gene ontology (GO) categories of biological processes, cellular components, molecular functions and pathways in which significant differentially expressed genes were grouped.
The table lists the biological processes that might be affected due to trisomy 21, GO categories are ranked by p-values and Benjamini-corrected p-values given by DAVID. The number of genes differentially expressed in each category is also shown.
