# Supplementary material for: Modelling and rescuing neurodevelopmental defect of Down syndrome using induced pluripotent stem cells from monozygotic twins discordant for trisomy 21
Source: EMBO Mol Med. 2013 Dec 27;6(2):259–77. doi: 10.1002/emmm.201302848 (PMC3927959; doi:10.1002/emmm.201302848)
Supplement: Supplementary file 7 [file emmm0006-0259-sd7.pdf]

Antibodies	Clone	ID	Source
OCT-3/4	C-10 mouse monoclonal	Sc-5279	Santa Cruz
SOX2	Y-17 goat polyclonal	Sc-17320	Santa Cruz
NANOG	H-155 rabbit polyclonal	Sc-33759	Santa Cruz
SSEA-4	813-70 mouse monoclonal	Sc-21704	Santa Cruz
TRA1-60	Mouse monoclonal	Sc-21705	Santa Cruz
TRA1-80	Mouse monoclonal	Sc-21706	Santa Cruz
α-SMA	HHF35 mouse monoclonal	Sc-53014	Santa Cruz
AFP	C3 mouse monoclonal	Sc-8399	Santa Cruz
β3-TUBULIN	TU-20 mouse monoclonal	Sc-51670	Santa Cruz
β3-TUBULIN	Rabbit polyclonal	PRB-435P	Covance
MAP2	Rabbit polyclonal	AB5622	Millipore
NESTIN	Rabbit polyclonal	AB5922	Millipore
GFAP	Rabbit polyclonal	Z0334	Dako
OLIG2	C-17 goat polyclonal	Sc-19969	Santa Cruz
Ki-67-FITC	Rabbit monoclonal	ab27619	Abcam
KI-67	Mouse monoclonal	Sc-23900	Santa Cruz
Cleaved caspase-3	Mouse monoclonal	AB10753	Millipore
SYNAPSIN	Rabbit polyclonal	AB1543	Millipore
GAD67	Mouse monoclonal	MAB5406	Chemicon International
PSD95	Goat plyclonal	Ab12093	Abcam

Supporting Information Table 4. List of the antibodies used for immunocytochemistry.
