# Supplementary material for: Modelling and rescuing neurodevelopmental defect of Down syndrome using induced pluripotent stem cells from monozygotic twins discordant for trisomy 21
Source: EMBO Mol Med. 2013 Dec 27;6(2):259–77. doi: 10.1002/emmm.201302848 (PMC3927959; doi:10.1002/emmm.201302848)
Supplement: Supplementary file 8 [file emmm0006-0259-sd8.pdf]

Semi-quantitative RT-PCR

Gene	Forward	Reverse	Amplicon
GAPDH	AGCCACATCGCTCAGACACC	GTACTCAGCGGCCAGCATCG	174
***OCT4* endo**	GCCTTCCTTCCCCATGGC	CCTCAAAATCCTCTCGTTGT	911
***OCT4* exo**	TCAAGCCTCAGACAGTGGTTC	CCTCAAAATCCTCTCGTTGT	976

Quantitative RT-PCR

Gene	Forward	Reverse
AFP	TTGACTGCAATTGAGAAACCCA	AAGGCAGGTAGCTGGTTTTCTAAA
T	TGCTTCCCTGAGACCCAGTT	GATCACTTCTTTCCTTTGCATCAAG
TUBB3	CGGTGGTGGAACCCTACAAC	AGGTGGTGACTCCGCTCAT
DLL1	ATATGCCCCAACGAATGCTG	GCTCGGTCTGAACTCGGTTTC
DYRK1A	CCTCTGTTCAGTGGTGCC	CCGTTTTCCATCTTTGGTC
eEF1	AGCAAAAATGACCCACCAATG	GGCCTGGATGGTTCAGGATA
FOXA2	GGAGCGGTGAAGATGGAAG	TACGTGTTCATGCCGTTCAT
GATA4	CTGGCCTGTCATCTCACTACG	GGTCCGTGCAGGAATTTGAGG
GFAP	AAGAGATCCGCACGCAGTAT	AGGTCAAGGACTGCAACTGG
GUSB	GAAGTATCAGAAGCCCATTATTCAGA	CAGAGGTGGATCCTGGTGAAA
HES1	AACACTGATTTTGGATGCTCTGAAG	CATTTCCAGAATGTCCGCCTT
LIN28	TGTAAGTGGTTCAACGTGCG	CCTCACCCTCCTTCAAGCTC
MAP2	AAAGCTGATGAGGGCAAGAA	GGCCCCTGAATAAATTCCAT
NANOG	GATTTGTGGGCCTGAAGAAA	TTGGGACTGGTGGAAGAATC
NES	GGAAGAGAACCTGGGAAAGG	CTTGGTCCTTCTCCACCGTA
NOTCH1	CGGGTCCACCAGTTTGAATG	GTTGTATTGGTTCGGCACCAT
NOTCH2	TCTGCCCTTGGACCCATTT	GGTACGCTGTGGTCCACAGAG
OCT4	AGTGCCCGAAACCCACACTG	ACCACACTCGGACCACATCCT
OLIG1	AAGTGACCAGAGCGGATGTTCGAT	TGAGAGCGAGCACTTTCTGCCTAA
OLIG2	GGACAAGCTAGGAGGCAGTG	ATGGCGATGTTGAGGTCGTG
PSD95	ACAAGCGGATCACAGAGGAG	CAGATGTAGGGGCCTGAGAG
REST/NRSF	GAGGAGGAGGGCTGTTTACC	TCACAGCAGCTGCCATTTAC
REX1	GAAGAGGCCTTCACTCTAGTAGTG	TTTCTGGTGTCTTGTCTTTGCCCG
S100B	CATCGACGTTTTCCACCAATA	TCGTGGCAGCGAGTAGTAAC
SMA	AATACTCTGTCTGGATCGGTGGCT	ACGAGTCAGAGCTTTGGCTAGGAA
SNAP25	CGATACACAGAATCGCCAGA	CAGCATCTTTGTTGCACGTT
SOX2	GCGAACCATCTCTGTGGTCT	GGAAAGTTGGGATCGAACAA
SYN1	GCAAAATACTTCAAAGGGAAAAAGA	TCTACTTTAATGTCAATTTCTCCATGGA
VIM	GAACGCCAGATGCGTGAAATG	CCAGAGGGAGTGAATCCAGATTA
WNT7A	AAGGTCTTTGTGGATGCCC	GCACTTACATTCCAGCTTCATG
WNT7B	TATCCCAGAGAGCAAAGTG	TGTGTTAGTGCCGAGAATC

Supporting Information Table 5. List of primers used in semi-quantitative and quantitative RT-PCR.
