# Supplementary material for: Modelling and rescuing neurodevelopmental defect of Down syndrome using induced pluripotent stem cells from monozygotic twins discordant for trisomy 21
Source: EMBO Mol Med. 2013 Dec 27;6(2):259–77. doi: 10.1002/emmm.201302848 (PMC3927959; doi:10.1002/emmm.201302848)
Supplement: Supplementary file 9 [file emmm0006-0259-sd9.pdf]

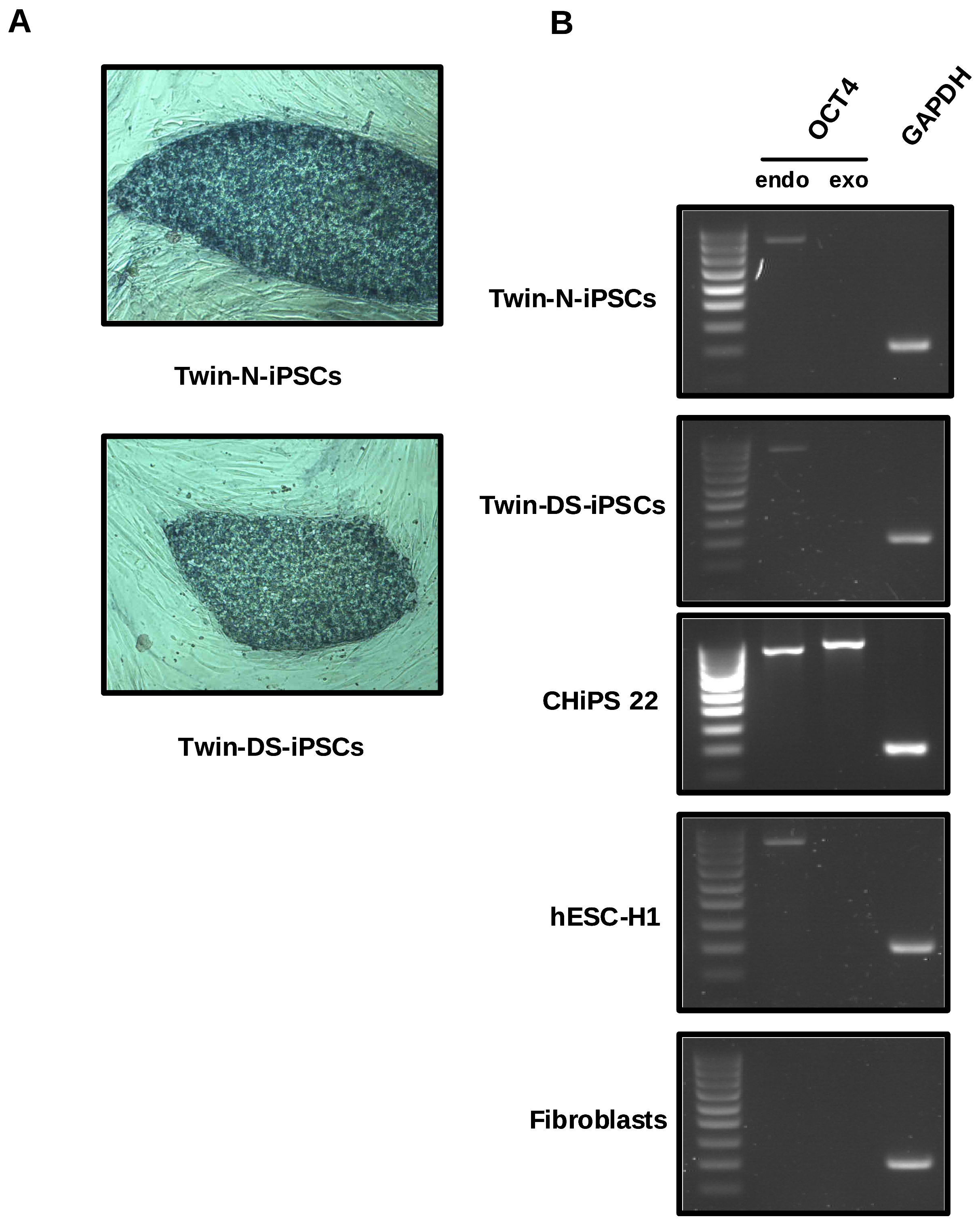

Supporting Information Fig 1. Additional controls validating the successful reprogramming and generation of Twin-N-iPSCs and Twin-DS-iPSCs.

(A) Alkaline phosphatase activity staining of Twin-N-iPSCs and Twin-DS-iPSCs.

(B) Transgene silencing in Twin-N-iPSCs and Twin-DS-iPSCs. As control, the iPSC line ChiPS22 showed the absence of transgene silencing (see Grad I *et al.*, 2011). The human embryonic stem hESC-H1 expressed only endogenous *OCT4* whereas the parental fibroblasts did not expressed *OCT4*.
