# Supplementary material for: Modelling and rescuing neurodevelopmental defect of Down syndrome using induced pluripotent stem cells from monozygotic twins discordant for trisomy 21
Source: EMBO Mol Med. 2013 Dec 27;6(2):259–77. doi: 10.1002/emmm.201302848 (PMC3927959; doi:10.1002/emmm.201302848)
Supplement: Supplementary file 10 [file emmm0006-0259-sd10.pdf]

A

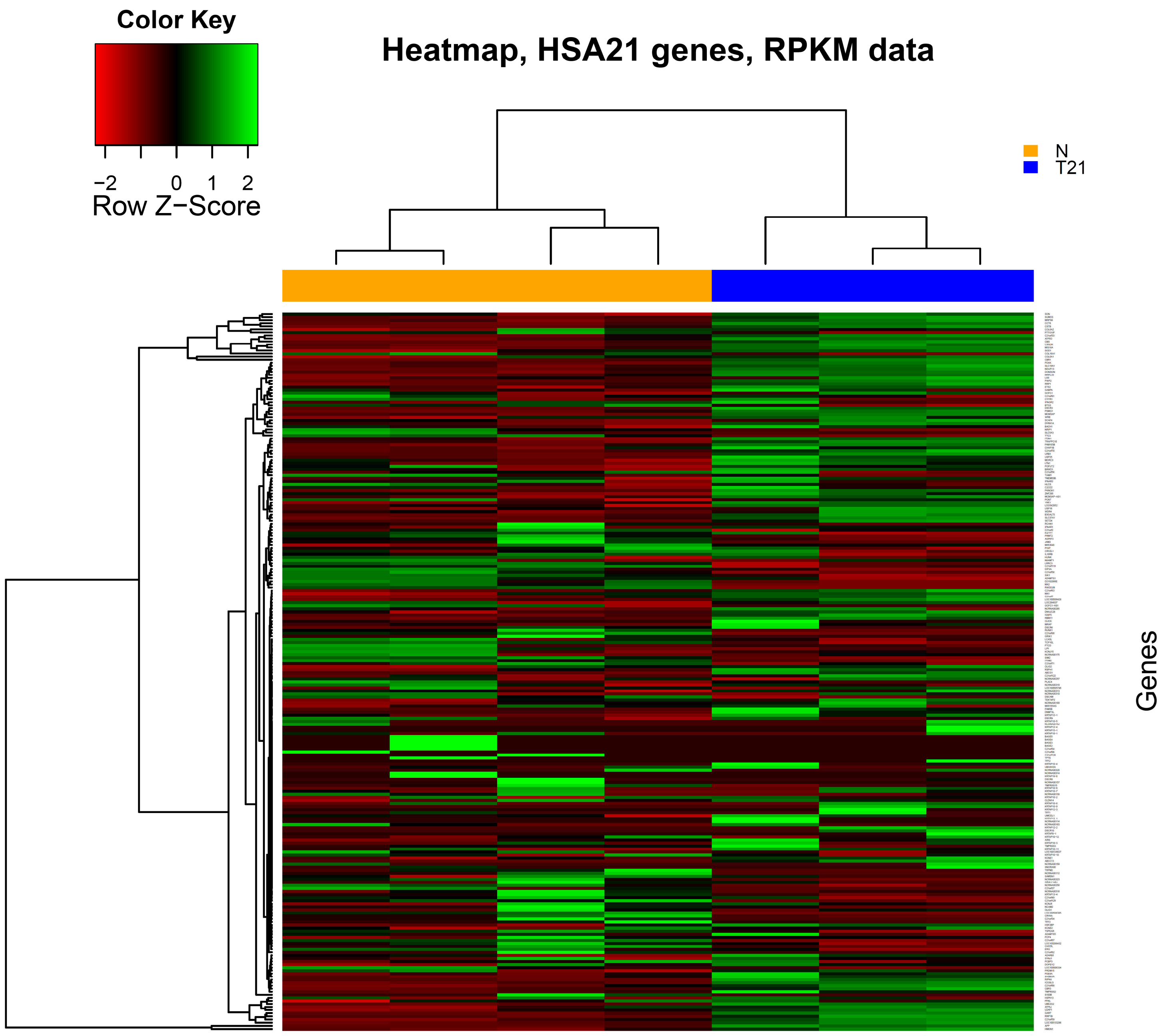

Color Key
−2 0 1 2
Row Z−Score
Heatmap, HSA21 genes, RPKM data
N
T21
Genes

B

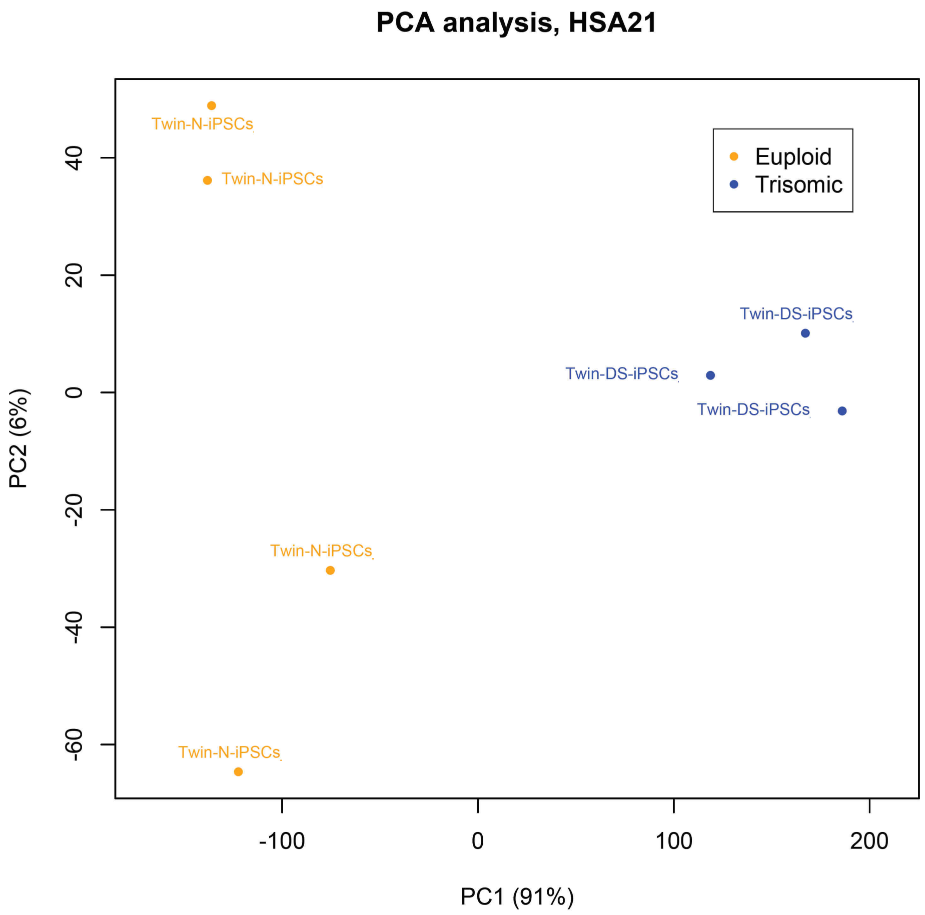

Supporting Information Fig 2. Heat map and principal component analysis (PCA) of the normalized expression values in Twin-N-iPSCs and Twin-DS-iPSCs when analyzing the HSA21 genes only.

(A) Samples (columns) and genes (rows) are sorted according to hierarchical clustering based on normalized and z-transformed gene expression values. A negative z-score (in red) indicates low expression (below the mean) whereas a positive z-score (in green) shows high expression (above the mean). **(B)** The two first principal components (PC1 and PC2) are plotted. The percentages represent the proportion of variance explained by each component. PC1 discriminates the trisomic (in blue) from the euploid (in orange) iPSC lines.
