# Supplementary material for: Modelling and rescuing neurodevelopmental defect of Down syndrome using induced pluripotent stem cells from monozygotic twins discordant for trisomy 21
Source: EMBO Mol Med. 2013 Dec 27;6(2):259–77. doi: 10.1002/emmm.201302848 (PMC3927959; doi:10.1002/emmm.201302848)
Supplement: Supplementary file 11 [file emmm0006-0259-sd11.pdf]

A

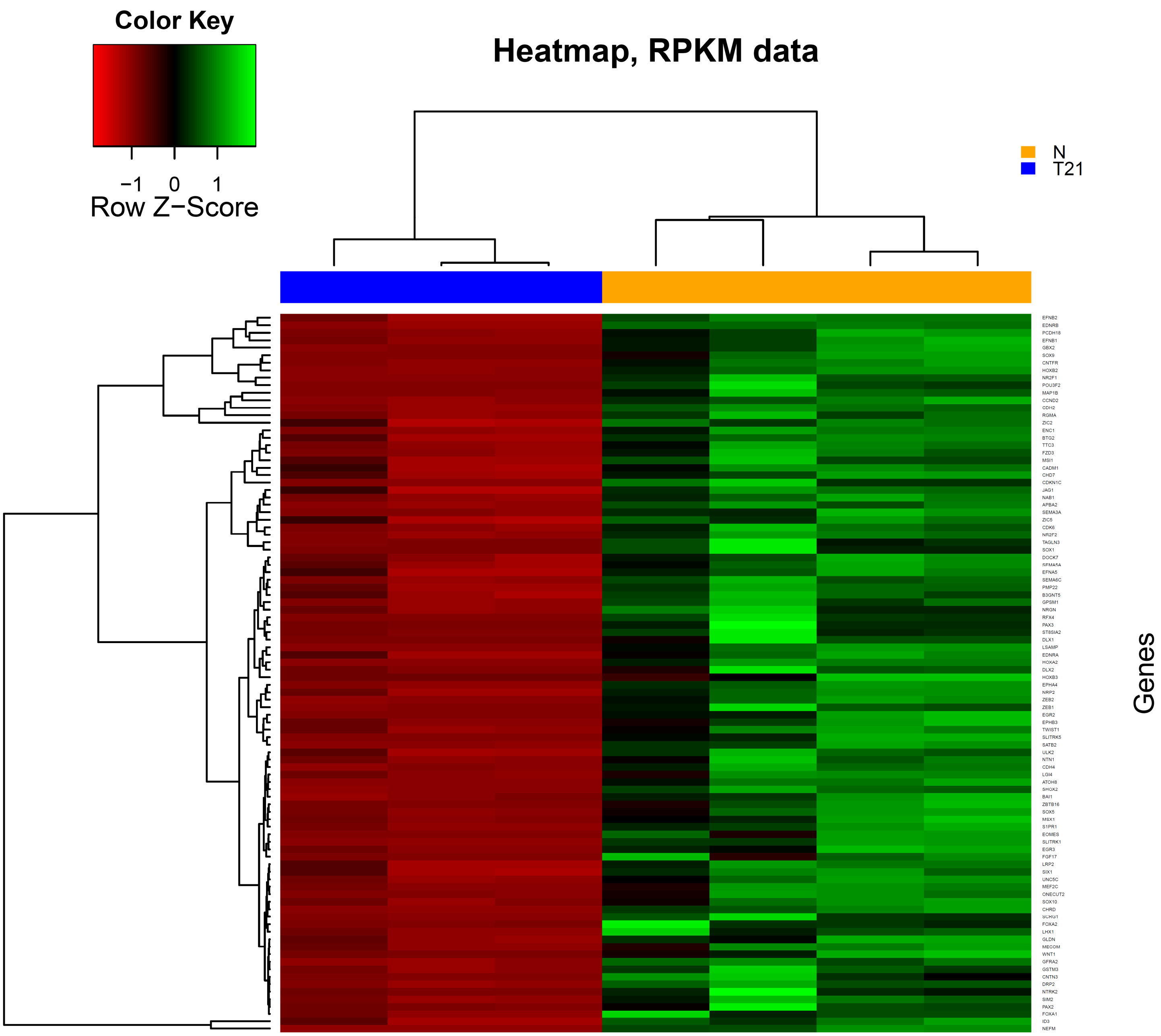
Color Key
−1
0
1
Row Z−Score
Heatmap, RPKM data
N
T21
Genes
EFNB2
EDNRB
PCDH18
EFNB1
GBX2
SOX9
CNTFR
HOXB2
NR2F1
POU3F2
MAP1B
CCND2
CDH2
RGMA
ZIC2
ENC1
BTG2
TTC3
FZD3
MSI1
CADM1
CHD7
CDKN1C
JAG1
NAB1
APBA2
SEMA3A
ZIC5
CDK6
NR2F2
TAGLN3
SOX1
DOCK7
SEMA5A
EFNA5
SEMA6C
PMP22
B3GNT5
GPSM1
NRGN
RFX4
PAX3
ST8SIA2
DLX1
LSAMP
EDNRA
HOXA2
DLX2
HOXB3
EPHA4
NRP2
ZEB2
ZEB1
EGR2
EPHB3
TWIST1
SLITRK5
SATB2
ULK2
NTN1
CDH4
LGI4
ATOH8
SHOX2
BAI1
ZBTB16
SOX5
MSX1
S1PR1
EOMES
SLITRK1
EGR3
FGF17
LRP2
SIX1
UNC5C
MEF2C
ONECUT2
SOX10
CHRD
SCRG1
FOXA2
LHX1
GLDN
MECOM
WNT1
GFRA2
GSTM3
CNTN3
DRP2
NTRK2
SIM2
PAX2
FOXA1
ID3
NEFM

B

Categories	Genes in the network	Count	*P*-value	Benjamini
Nervous system development (GO:0007399)	NRP2, MEF2C, FOXA2, GLDN, CADM1, FGF17, ZEB2, ZEB1, PAX3, JAG1, PAX2, ZIC2, EDNRB, WNT1, GSTM3, S1PR1, SCRG1, LGI4, ATOH8, SEMA3A, UNC5C, NR2F2, NR2F1, TWIST1, SOX10, SATB2, EGR3, EGR2, EFNB1, ENC1, EFNB2, EOMES, CDK6, MECOM, SLITRK1, MSX1, BTG2, CCND2, LSAMP, NAB1, SIX1, EFNA5, CNTN3, NRGN, PMP22, SLITRK5, SOX1, ONECUT2, SOX5, CNTFR, CDH2, ZBTB16, ST8SIA2, EPHB3, TAGLN3, CDH4, TTC3, SEMA5A, RGMA, HOXA2, CHD7, C11ORF9, B3GNT5, LHX1, BAI1, MSI1, GPSM1, GBX2, APBA2, POU3F2, NEFM, SIM2, RFX4, MAP1B, FOXA1, FZD3, DOCK7, NTN1, PCDH18, CDKN1C, SHOX2, HOXB3, EPHA4, DLX2, DLX1, HOXB2, SEMA6C, DRP2, ULK2, ZIC5, NTRK2, ID3, LRP2, CHRD, GFRA2	95	2.30E-21	8.20E-19
Neurogenesis (GO:0022008)	NRP2, FOXA2, JAG1, PAX3, PAX2, WNT1, S1PR1, LGI4, UNC5C, SEMA3A, NR2F2, TWIST1, NR2F1, SOX10, SATB2, EGR2, EFNB1, CDK6, SLITRK1, BTG2, CCND2, NAB1, SIX1, EFNA5, SLITRK5, SOX1, ONECUT2, SOX5, CDH2, EPHB3, CDH4, TTC3, SEMA5A, HOXA2, C11ORF9, LHX1, BAI1, GBX2, POU3F2, NEFM, FOXA1, MAP1B, DOCK7, NTN1, CDKN1C, DLX2, EPHA4, DLX1, SEMA6C, ULK2, NTRK2, ID3	52	2.20E-11	2.30E-09
Generation of neurons (GO:0048699)	NRP2, FOXA2, SOX1, ONECUT2, SOX5, PAX3, JAG1, CDH2, EPHB3, PAX2, CDH4, TTC3, SEMA5A, WNT1, HOXA2, S1PR1, LHX1, BAI1, GBX2, LGI4, POU3F2, UNC5C, SEMA3A, NR2F2, NEFM, TWIST1, NR2F1, SATB2, EGR2, EFNB1, MAP1B, FOXA1, DOCK7, NTN1, CDKN1C, SLITRK1, DLX2, EPHA4, DLX1, SEMA6C, BTG2, CCND2, ULK2, NTRK2, SIX1, EFNA5, ID3, SLITRK5	48	1.90E-10	1.80E-08
Neuron differentiation (GO:0030182)	NRP2, SOX1, FOXA2, ONECUT2, SOX5, PAX3, JAG1, EPHB3, PAX2, CDH4, SEMA5A, WNT1, HOXA2, S1PR1, LHX1, BAI1, GBX2, LGI4, POU3F2, UNC5C, SEMA3A, EGR2, EFNB1, MAP1B, FOXA1, DOCK7, NTN1, CDKN1C, SLITRK1, DLX2, EPHA4, DLX1, SEMA6C, BTG2, ULK2, NTRK2, EFNA5, ID3, SLITRK5	39	5.10E-09	3.90E-07
Central nervous system development (GO:0007417)	SOX1, FOXA2, SOX5, ZEB2, PAX3, ZBTB16, ZEB1, EPHB3, TAGLN3, ZIC2, WNT1, HOXA2, CHD7, S1PR1, B3GNT5, C11ORF9, LHX1, GBX2, POU3F2, UNC5C, NR2F2, NR2F1, SOX10, EGR2, RFX4, MAP1B, EOMES, MECOM, PCDH18, DLX2, DLX1, HOXB2, MSX1, DRP2, ZIC5, EFNA5, LRP2, CHRD	38	7.40E-09	4.70E-07
Peripheral nervous system development (GO:0007422)	SOX10, EDNRB, GSTM3, EGR3, EGR2, BAI1, NAB1, ONECUT2, POU3F2, PMP22	10	7.40E-09	4.70E-07
Axonogenesis (GO:0007409)	NRP2, EGR2, EFNB1, MAP1B, DOCK7, PAX2, EPHB3, CDH4, NTN1, SEMA5A, SLITRK1, EPHA4, HOXA2, SEMA6C, ULK2, BAI1, GBX2, EFNA5, SEMA3A, UNC5C, SLITRK5	21	8.90E-07	3.80E-05
Brain development (GO:0007420)	SOX1, FOXA2, ZEB2, ZIC2, WNT1, HOXA2, S1PR1, LHX1, GBX2, POU3F2, UNC5C, NR2F2, NR2F1, EGR2, RFX4, EOMES, MECOM, PCDH18, DLX2, DLX1, MSX1, HOXB2, ZIC5, EFNA5, LRP2, CHRD	26	2.40E-06	8.00E-05
Cell morphogenesis involved in neuron differentiation (GO:0048667)	NRP2, EGR2, EFNB1, MAP1B, DOCK7, PAX2, EPHB3, CDH4, NTN1, SEMA5A, SLITRK1, EPHA4, HOXA2, SEMA6C, ULK2, BAI1, GBX2, EFNA5, SEMA3A, UNC5C, SLITRK5	21	5.60E-06	1.60E-04
Neuron projection morphogenesis (GO:0048812)	NRP2, EGR2, EFNB1, MAP1B, DOCK7, PAX2, EPHB3, CDH4, NTN1, SEMA5A, SLITRK1, EPHA4, HOXA2, SEMA6C, ULK2, BAI1, GBX2, EFNA5, SEMA3A, UNC5C, SLITRK5	21	7.40E-06	2.00E-04
Axon guidance (GO:0007411)	NRP2, EGR2, EFNB1, EPHB3, NTN1, CDH4, SEMA5A, EPHA4, HOXA2, SEMA6C, GBX2, EFNA5, SEMA3A, UNC5C	14	2.00E-05	4.70E-04
Neural tube development (GO:0021915)	RGMA, WNT1, FOXA2, ZIC5, GBX2, FZD3, ZEB2, PAX3, CHRD, ZIC2, TWIST1	11	3.50E-05	6.90E-04
Neuron development (GO:0048666)	NRP2, SOX1, ONECUT2, EPHB3, PAX2, CDH4, SEMA5A, HOXA2, BAI1, GBX2, LGI4, SEMA3A, UNC5C, EGR2, EFNB1, MAP1B, DOCK7, NTN1, CDKN1C, SLITRK1, EPHA4, SEMA6C, ULK2, NTRK2, EFNA5, SLITRK5	26	3.80E-05	6.90E-04
Neural crest cell development (GO:0014032)	NRP2, EDNRA, EDNRB, EFNB1, GBX2, ZEB2, PAX3, SOX9	8	4.80E-05	9.00E-04
Neural crest cell differentiation (GO:0014033)	NRP2, EDNRA, EDNRB, EFNB1, GBX2, ZEB2, PAX3, SOX9	8	4.80E-05	9.00E-04
Regulation of neuron differentiation (GO:0045664)	FOXA2, FOXA1, MAP1B, SOX5, CDH2, NTN1, CDH4, TTC3, HOXA2, CCND2, SIX1, NTRK2, POU3F2, SEMA3A, NEFM	15	5.00E-05	9.30E-04
Neuron projection development (GO:0031175)	NRP2, EGR2, EFNB1, MAP1B, DOCK7, PAX2, EPHB3, CDH4, NTN1, SEMA5A, SLITRK1, EPHA4, HOXA2, SEMA6C, ULK2, BAI1, GBX2, EFNA5, SEMA3A, UNC5C, SLITRK5	21	1.00E-04	1.90E-04
Forebrain development (GO:0030900)	RFX4, SOX1, ZEB2, MECOM, WNT1, DLX2, DLX1, MSX1, LHX1, ZIC5, POU3F2, LRP2, NR2F2, CHRD, NR2F1	15	2.10E-04	3.50E-03
Neural crest cell migration (GO:0001755)	NRP2, EDNRB, EFNB1, GBX2, ZEB2, PAX3	6	5.30E-04	7.90E-03

Supporting Information Fig 3. Heat map of the normalized gene expression values in Twin-DS-iPSCs and Twin-N-iPSCs for the 96 downregulated genes involved in brain-related functions.

(A) Samples (columns) and genes (rows) are sorted according to hierarchical clustering based on normalized and z-transformed gene expression values. A negative z-score (in red) indicates low expression (below the mean) whereas a positive z-score (in green) shows high expression (above the mean). **(B)** The table lists the brain-related functions associated with these 96 downregulated genes.
