# Supplementary material for: Modelling and rescuing neurodevelopmental defect of Down syndrome using induced pluripotent stem cells from monozygotic twins discordant for trisomy 21
Source: EMBO Mol Med. 2013 Dec 27;6(2):259–77. doi: 10.1002/emmm.201302848 (PMC3927959; doi:10.1002/emmm.201302848)
Supplement: Supplementary file 12 [file emmm0006-0259-sd12.pdf]

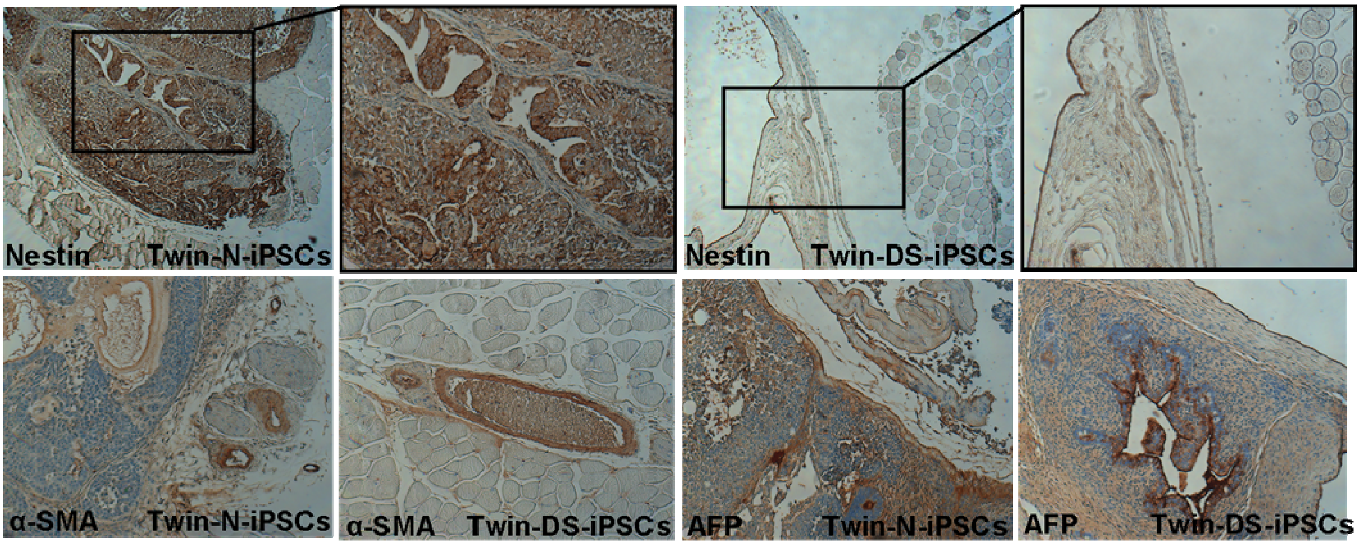

Supporting Information Fig 4. Additional controls validating the absence of ectodermal structures observed in Twin-DS-iPSC-derived teratomas.

Immunohistochemistry analysis revealed the near absence of staining for the neuroepithelial marker NESTIN in teratomas derived from Twin-DS-iPSCs (upper panel, right images) in comparison with those derived from Twin-N-iPSCs (upper panel, left images). Both teratomas expressed the mesodermal marker (α-SMA; lower panel, left images) and the endodermal marker (AFP, lower panel, right images).
