# Supplementary material for: Modelling and rescuing neurodevelopmental defect of Down syndrome using induced pluripotent stem cells from monozygotic twins discordant for trisomy 21
Source: EMBO Mol Med. 2013 Dec 27;6(2):259–77. doi: 10.1002/emmm.201302848 (PMC3927959; doi:10.1002/emmm.201302848)
Supplement: Supplementary file 13 [file emmm0006-0259-sd13.pdf]

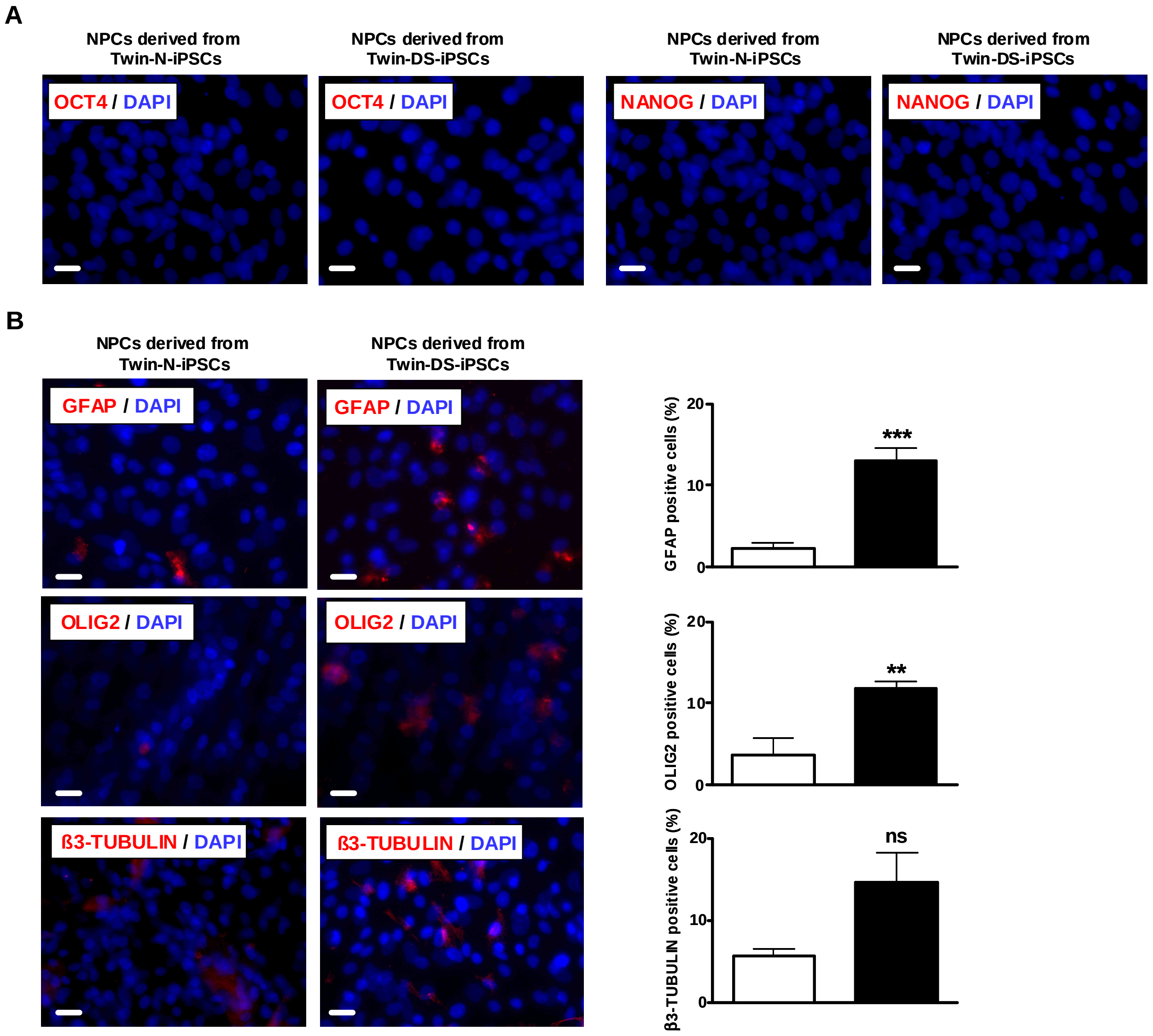

Supporting Information Fig 5. Proportion of each cell type in neurospheres derived from Twin-N-iPSCs and Twin-DS-iPSCs upon neural induction

(**A**) Immunofluorescence staining of cells derived from Twin-N-iPSCs and Twin-DS-iPSCs upon neural induction for the pluripotency markers OCT4 and NANOG. (**B**) Proportion of $GFAP^+$ cells, $OLIG2^+$ cells and β3-$TUBULIN^+$ cells upon neural induction of Twin-N-iPSCs and Twin-DS-iPSCs. Scale bar corresponds to 10 µm. Data are represented as mean ± SEM. Ns non significant, ** $p < 0.01$, *** $p < 0.001$ by Student's *t*-test from n = 4.
