# Supplementary material for: Modelling and rescuing neurodevelopmental defect of Down syndrome using induced pluripotent stem cells from monozygotic twins discordant for trisomy 21
Source: EMBO Mol Med. 2013 Dec 27;6(2):259–77. doi: 10.1002/emmm.201302848 (PMC3927959; doi:10.1002/emmm.201302848)
Supplement: Supplementary file 14 [file emmm0006-0259-sd14.pdf]

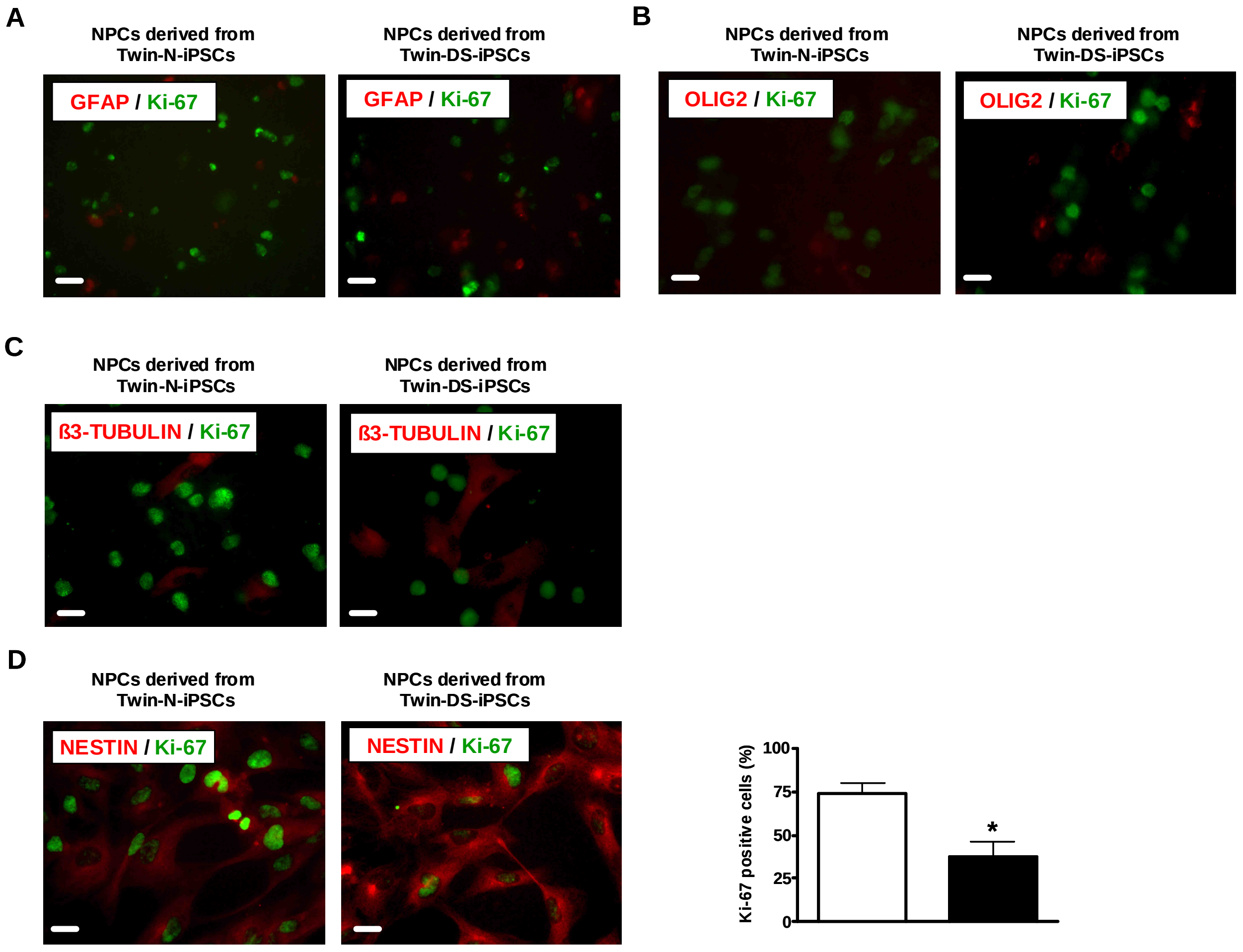

Supporting Information Fig 6. Proliferative properties of each cell type in neurospheres derived from Twin-N-iPSCs and Twin-DS-iPSCs upon neural induction.
(A-D) Representative images of each cell type in neurospheres derived from Twin-N-iPSCs and Twin-DS-iPSCs co-stained with GFAP (in **A**), OLIG2 (in **B**), β3-TUBULIN (in **C**), NESTIN (in **D**) and Ki-67 antibodies. $GFAP^{+}$ cells (in **A**), $OLIG2^{+}$ cells (in **B**), $β3\text{-}TUBULIN^{+}$ cells (in **C**) were negative for Ki-67 staining. Proportion of Ki-67 positive cells among $NESTIN^{+}$ cells in neurospheres derived from Twin-N-iPSCs and Win-DS-iPSCs (in **D**). Scale bar corresponds to 10 µm. Data are represented as mean ± SEM. * $p < 0.05$ by Student's *t*-test from n = 4.
