# Supplementary material for: Modelling and rescuing neurodevelopmental defect of Down syndrome using induced pluripotent stem cells from monozygotic twins discordant for trisomy 21
Source: EMBO Mol Med. 2013 Dec 27;6(2):259–77. doi: 10.1002/emmm.201302848 (PMC3927959; doi:10.1002/emmm.201302848)
Supplement: Supplementary file 15 [file emmm0006-0259-sd15.pdf]

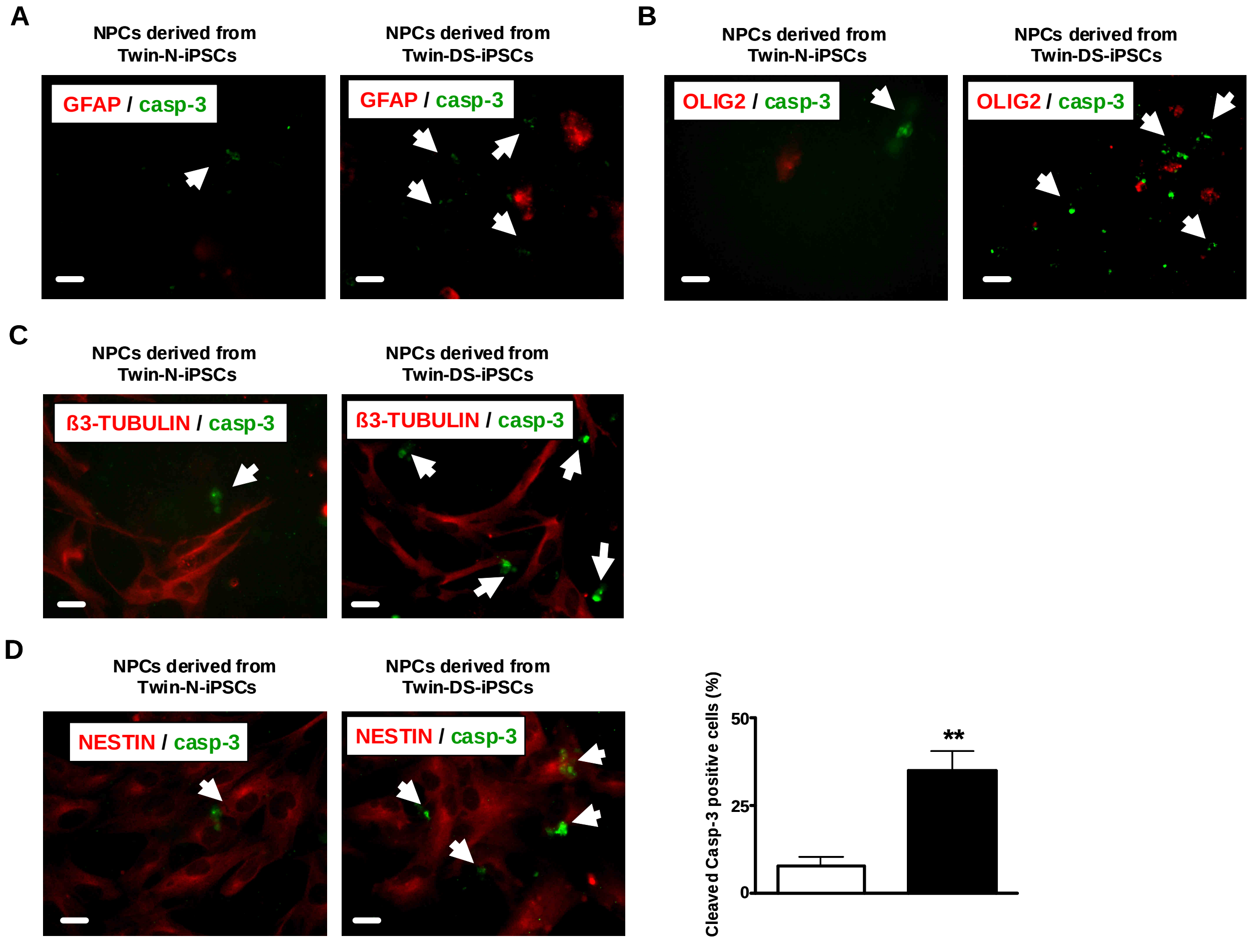

Supporting Information Fig 7. Apoptotic properties of each cell type in neurospheres derived from Twin-N-iPSCs and Twin-DS-iPSCs upon neural induction.

(A-D) Representative images of each cell type of neurospheres derived from Twin-N-iPSCs and Twin-DS-iPSCs co-stained with GFAP (in **A**), OLIG2 (in **B**), β3-TUBULIN (in **C**), NESTIN (in **D**) and cleaved caspase-3 (arrows) antibodies. $GFAP^+$ cells (in **A**), $OLIG2^+$ cells (in **B**), β3-$TUBULIN^+$ cells (in **C**) were negative for cleaved caspase-3 staining. **(D)** Proportion of cleaved caspase-3^+ cells among $NESTIN^+$ cells in neurospheres derived from Twin-N-iPSCs and Twin-DS-iPSCs (in **D**). Scale bar corresponds to 10 µm. Data are represented as mean ± SEM. ** $p < 0.01$ by Student's *t*-test from $n = 4$.
