# Supplementary material for: Modelling and rescuing neurodevelopmental defect of Down syndrome using induced pluripotent stem cells from monozygotic twins discordant for trisomy 21
Source: EMBO Mol Med. 2013 Dec 27;6(2):259–77. doi: 10.1002/emmm.201302848 (PMC3927959; doi:10.1002/emmm.201302848)
Supplement: Supplementary file 16 [file emmm0006-0259-sd16.pdf]

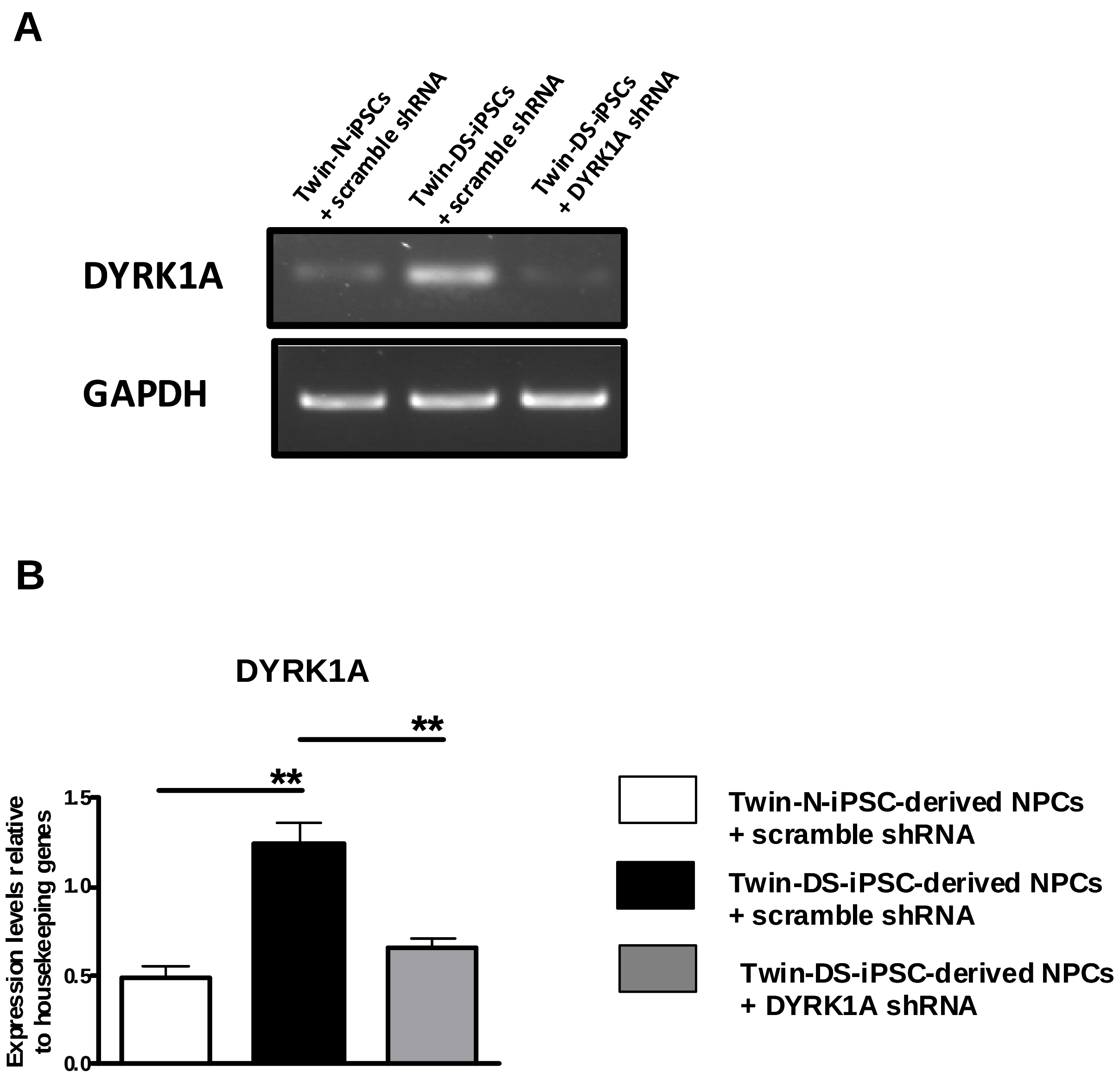

Supporting Information Fig 8. Knockdown efficiencies of DYRK1A shRNA in NPCs derived from Twin-DS-iPSCs.

(A) Non quantitative RT-PCR showing knockdown efficiencies of DYRK1A shRNA in Twin-DS-iPSCs. **(B)** Quantitative RT-PCR showing knockdown efficiencies of DYRK1A shRNA in NPCs derived from Twin-DS-iPSCs. Data are represented as mean ± SEM. ** $p < 0.01$ by one-way ANOVA followed with Tukey's post hoc test from n = 4.
