# Supplementary material for: Modelling and rescuing neurodevelopmental defect of Down syndrome using induced pluripotent stem cells from monozygotic twins discordant for trisomy 21
Source: EMBO Mol Med. 2013 Dec 27;6(2):259–77. doi: 10.1002/emmm.201302848 (PMC3927959; doi:10.1002/emmm.201302848)
Supplement: Supplementary file 17 [file emmm0006-0259-sd17.pdf]

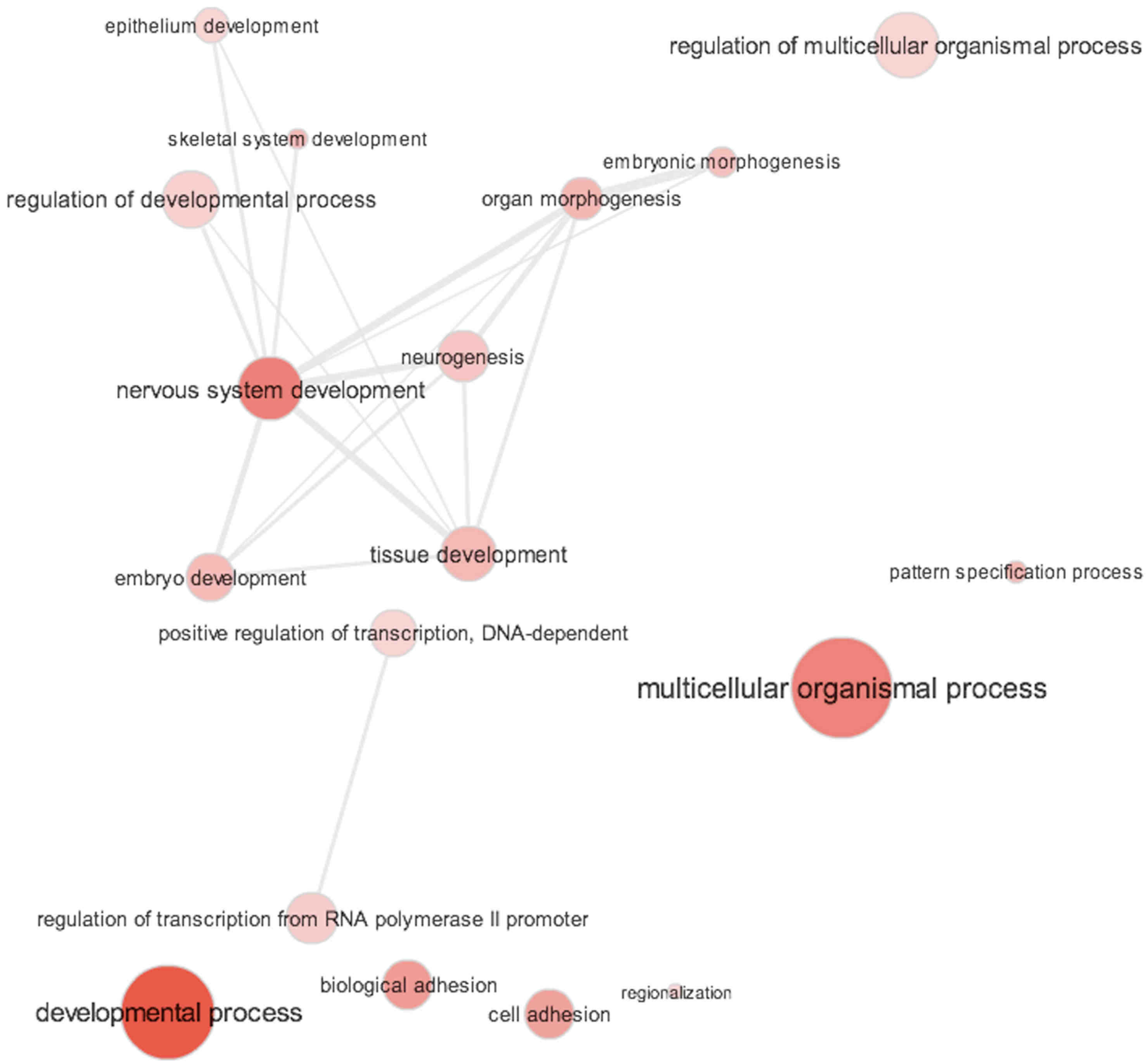


**Supporting Information Fig 9. REVIGO interactive graph of the top biological processes associated with the 580 downregulated genes.**
The node size represents the frequency of the GO term in the human database and the color shows the p-value associated with each term (dark red for small p-values).
