# Supplementary material for: Modelling and rescuing neurodevelopmental defect of Down syndrome using induced pluripotent stem cells from monozygotic twins discordant for trisomy 21
Source: EMBO Mol Med. 2013 Dec 27;6(2):259–77. doi: 10.1002/emmm.201302848 (PMC3927959; doi:10.1002/emmm.201302848)
Supplement: Supplementary file 18 [file emmm0006-0259-sd18.pdf]

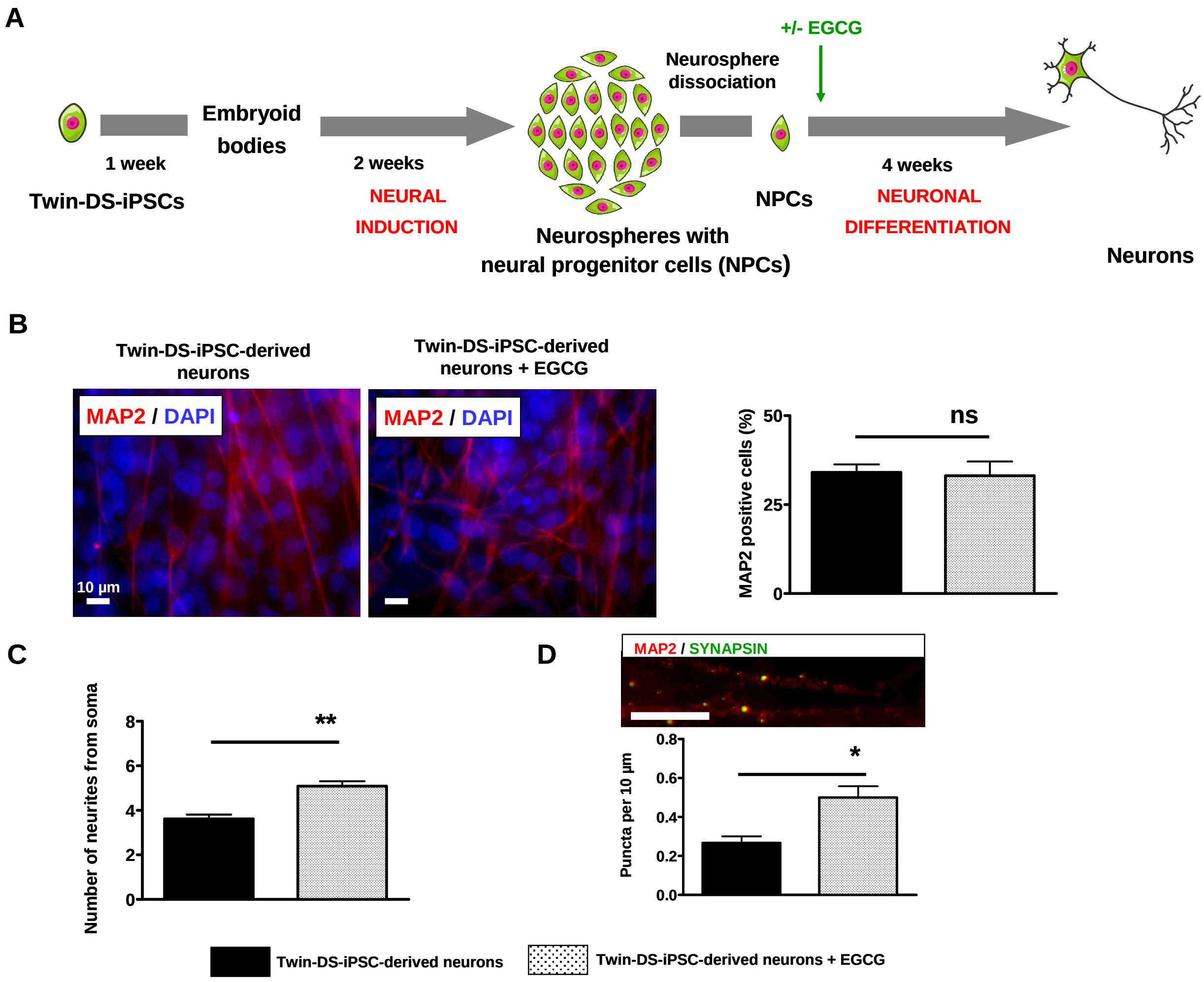

Supporting Information Fig 10. Additional effects of a delayed targeting of DYRK1A through EGCG on neurons derived from Twin-DS-iPSCs

(A) Schematic representation for generation of neurons from Twin-DS-iPSCs after a delayed targeting of DYRK1A through EGCG 10 µM. **(B)** Proportion of MAP2 positive cells after neuronal differentiation of Twin-DS-iPSC-derived NPCs. **(C)** Increased number of neurites from soma of neurons derived from Twin-DS-iPSCs upon EGCG treatment. **(D)** Representative image of SYNAPSIN I punctae in MAP2$^+$ dendrites. Reduced density of SYNAPSIN punctae in neurons derived from Twin-DS-iPSCs. Data are represented as mean ± SEM. Ns non significant, * $p < 0.05$, ** $p < 0.01$ by Student's *t*-test from n = 3-4.
