# Supplementary material for: Modelling and rescuing neurodevelopmental defect of Down syndrome using induced pluripotent stem cells from monozygotic twins discordant for trisomy 21
Source: EMBO Mol Med. 2013 Dec 27;6(2):259–77. doi: 10.1002/emmm.201302848 (PMC3927959; doi:10.1002/emmm.201302848)
Supplement: Supplementary file 19 [file emmm0006-0259-sd19.pdf]

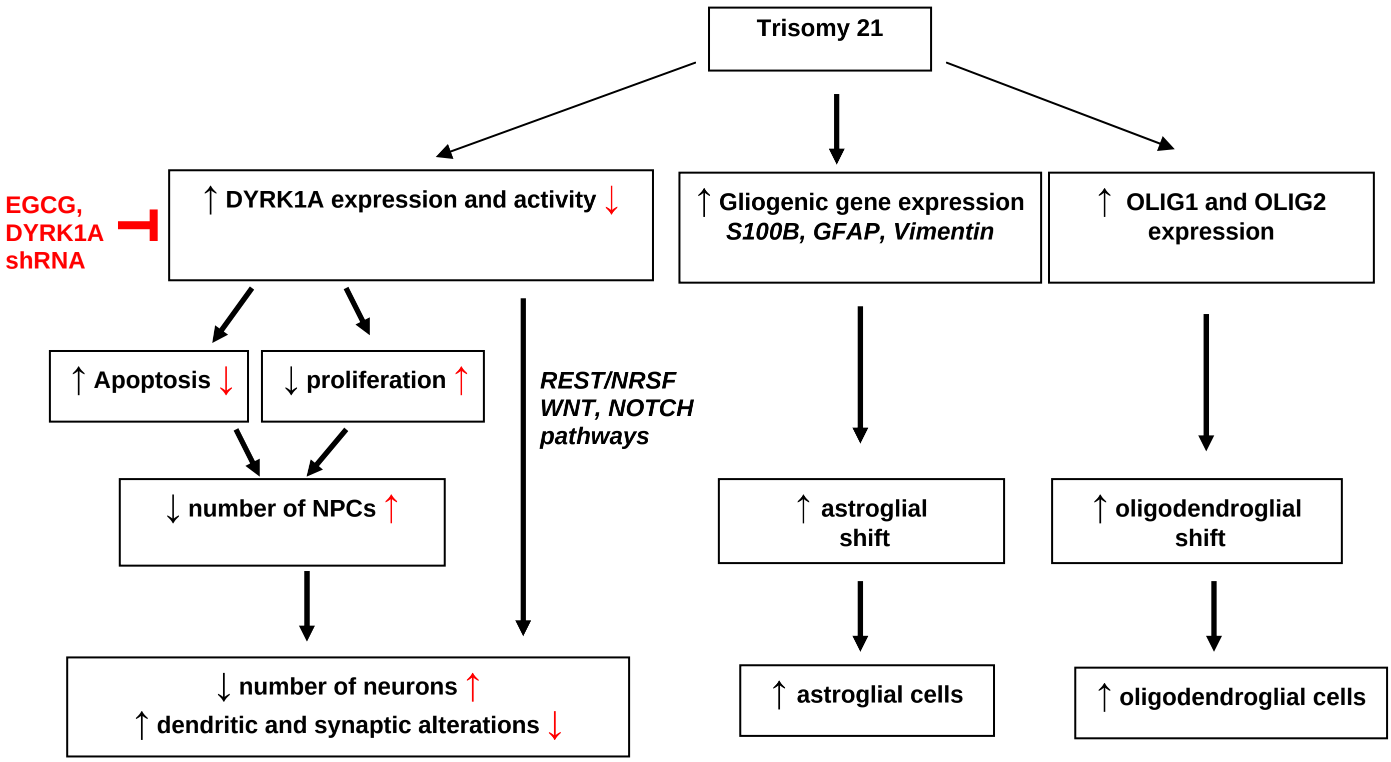

Supporting Information Fig 11. Proposed mechanisms responsible for the abnormal neuronal differentiation of Twin-DS-iPSCs.
In DS, the extra copy of HSA21 leads to the overexpression of HSA21 gene such as *DYRK1A*, *S100B*, *OLIG1* and *OLIG2*. Here, we proposed that a possible mechanism for the abnormal neuronal differentiation of Twin-DS-iPSCs is related to DYRK1A. The increased expression and activity of DYRK1A in NPCs derived from Twin-DS-iPSCs lead to an increase of cell apoptosis and a decrease of cell proliferation contributing to a reduced number of these cells. Moreover, upon neuronal differentiation, Twin-DS-iPSCs exhibited a reduced number of neurons and an increase of astroglial and oligodendroglial cells compared to Twin-N-iPSCs. In line with this, neurons derived from Twin-DS-iPSCs showed dendritic and synaptic alterations. Importantly, DYRK1A inhibition though EGCG treatment and shRNA silencing corrected these defects. Genes and pathways involved in neural fate and differentiation such as *REST/NRSF*, *WNT* and *NOTCH* likely underlie DYRK1A correction (→: stimulation; ├: inhibition).
