# Supplementary material for: Modelling and rescuing neurodevelopmental defect of Down syndrome using induced pluripotent stem cells from monozygotic twins discordant for trisomy 21
Source: EMBO Mol Med. 2013 Dec 27;6(2):259–77. doi: 10.1002/emmm.201302848 (PMC3927959; doi:10.1002/emmm.201302848)
Supplement: Supplementary file 20 [file emmm0006-0259-sd20.pdf]

EMBO Molecular Medicine

Manuscript EMM-2013-02848

Modelling and rescuing neurodevelopmental defect of Down syndrome using induced pluripotent stem cells from monozygotic twins discordant for trisomy 21

Youssef Hibaoui, Iwona Grad, Audrey Letourneau, M. Reza Sailani, Sophie Dahoun, Federico A. Santoni, Stefania Gimelli, Michel Guipponi, Marie Françoise Pelte, Frédérique Béna, Stylianos E. Antonarakis and Anis Feki

Corresponding authors: Anis Feki, Service de gynécologie obstétrique, HFR Fribourg - Hôpital cantonal
Stylianos E. Antonarakis, Department of Genetic Medicine and Development, University of Geneva Medical School and Geneva University Hospitals

Editor: Céline Carret

Review timeline:

Submission date:	04 April 2013
Editorial Decision:	10 May 2013
Revision received:	24 September 2013
Editorial Decision:	28 October 2013
Revision received:	11 November 2013
Accepted:	12 November 2013

Transaction Report:

(Note: With the exception of the correction of typographical or spelling errors that could be a source of ambiguity, letters and reports are not edited. The original formatting of letters and referee reports may not be reflected in this compilation.)

1st Editorial Decision 10 May 2013

Thank you for the submission of your manuscript to EMBO Molecular Medicine. We have now heard back from the three referees whom we asked to evaluate your manuscript. Although they find the study to be of potential interest, they also raise a number of concerns that need to be addressed in a major revision of the manuscript.

As you will see from the reports below, all three Reviewers find the topic interesting. Referees 1 and 2 are concerned about the limited mechanistic insights and require clarifications and answers as well as new experiments to improve this aspect of the study, as indicated. Referee 1 would also like to see additional data to increase the therapeutic/clinical impact of the study while Referees 2 and 3 raise issues on inappropriate/absent quantifications and statistical analyses. Importantly all three Referees agree that changes should be made in the references section.

Given the balance of these evaluations, we feel that we can consider a revision of your manuscript if you can convincingly address all issues raised. Please note that it is EMBO Molecular Medicine policy to allow only a single round of revision and that, as acceptance or rejection of the manuscript

will depend on another round of review, your responses should be as complete as possible.

EMBO Molecular Medicine has a "scooping protection" policy, whereby similar findings that are published by others during review or revision are not a criterion for rejection. Should you decide to submit a revised version, I do ask that you get in touch after three months if you have not completed it, to update us on the status.

Please also contact us as soon as possible if similar work is published elsewhere. If other work is published we may not be able to extend the revision period beyond three months.

I look forward to receiving your revised manuscript.

***** Reviewer's comments *****

Referee #1 (Comments on Novelty/Model System):

In this study, the authors established an induced pluripotent stem cell (iPSC) model to study Down syndrome related neurodevelopmental defects. Pluripotent stem cells were generated from monozygotic twins discordant for trisomy 21 thereby eliminating the influence of variable genetic background. The authors used state of the art technologies for validating and determining the transcriptional signature of iPSC lines. They confirmed previous observations on reduced neurogenesis and deficient neuronal differentiation in Down syndrome (DS) cultures. The data on the role of DYRK1A are novel and interesting. The expression of this enzyme was significantly increased in Twin-DS cultures. The pharmacological inhibition or the knockdown of the expression of this enzyme partially corrected the deficit in neurogenesis and neuronal differentiation. Overall, the actual insight for understanding the pathogenic mechanisms remains weak.

1. The authors demonstrate a reduced number of SOX2/Nestin positive NPCs and Ki67 positive cells in DS derived cultures. Ki67 was not co-localized with cell type-specific antigenic markers. Since these preparations likely to represent a mixed population of cells including pluripotent stem cells, multipotent neural progenitors and committed neuroblasts, the authors should provide additional data on the proliferation rate of these different populations. For this purpose, Ki67 or BRDU should be co-localized with cell type-specific markers.

2. Similarly, it would be important to know which cell type displays increased levels of apoptotic markers.

3. The data on altered differentiation and maturation of neurons are rather sketchy. The possible shift in the proportion of neuronal subtypes in DS versus control cultured has not been explored. It is possible that trisomy 21 favors glial as well as GABA-ergic differentiation at the expense of the glutamatergic neuronal population. Changes in cell type specification could influence the readout of neuronal process length and arborization measures.

4. A key issue is the capacity of neurons to form synaptically interconnected networks. The authors measured some synaptic proteins and emphasized synaptic alterations in Twin-DS cultures. However, they do not demonstrate that iPSC derived neurons could make synaptic contacts, do not explore synaptic functions and do not investigate the possibility that these structures could be different between DS and control cultures.

5. Although, it has been predicted that the pharmacological inhibition of DYRK1A may have a potential role in DS therapy (for example see Mazur-Kolecka et al., 2012) (this reference should be cited), the present study provides the first direct support to this hypothesis in a human cell culture model. DYRK1A have established roles in the sequential steps of neurogenesis and neuronal differentiation. The impact of inhibiting DYRK1A on early events including neuronal proliferation and cell death appears obvious. The authors should provide additional data on effects of this treatment on late events including neuronal process formation and synaptogenesis using a delayed targeting of DYRK1A, during neuronal differentiation after the proliferative phase and neuronal commitment. This information would be essential for evaluating the therapeutic potential of this treatment of DS in adulthood.

6. It is not clear to me whether multiple comparisons or simple t-tests were performed to analyze data in Fig 5 and 6.

Referee #2 (Comments on Novelty/Model System):

The use of iPSCs is what makes this work novel and significant in Down syndrome research. Quality of the experiments is OK, although additional details about the methodology used and the number of biological replicates done are required.

Referee #2 (Remarks):

This manuscript reports the comparison of the transcriptome and differentiation potential of iPSCs derived from fetal fibroblast isolated from monozygotic twins, one with a normal kariotype and the other with and extra chromosome 21. It also shows that neural progenitor cells (NPCs) derived from trisomic iPSCs express aberrant levels of neural and glial markers and they proliferate less and are less neurogenic than NPCs derived from euploid iPSCs. The authors went on and tested whether they could rescue some of the defects observed in trisomic NPCs by knocking down one of the chromosome 21 genes, DYRK1A, using shRNA or by pharmacological inhibition of DYRK1A kinase activity.

The experiments are well designed and the results novel. Characterization of the iPSC lines is rigorous. The reported cell lines represent an excellent model to study the impact of an extra chromosome 21 in different cell types and to identify the pathways and genes involved in Down syndrome. The results presented on DYRK1A are also significant showing for the first time the effect of an extra copy of the gene in the behavior of human trisomic NPCs.

Comments and questions:
1. The kariotype of one Twin-N and one Twin-DS iPSC lines is shown in Fig 2A. Did the authors check the kariotype of the neurosphere-derived NPCs used in the differentiation experiments? How many cell lines of the each genotype were used in the experiments? The authors should provide this information and indicate in the Method section or in the legend to the figure the number of independent cell lines used, the number of independent experiments done and the number of replicates in each experiment.

2. One of the interesting results shown in the manuscript is the increase in apoptosis in DS-NPC cultures. Is apoptosis in these cells also increased when cultured in differentiation media? In other word, are N-NPC and DS-NPC cultures equally dense after the differentiation period? As differentiation and maturation of neurons and glial cells depends on cell density this information may be relevant for the interpretation of the results shown in Fig 4.

3. B3 TUBULIN immunostaining in DS-neurons is very faint compared to the immunostaining in N-neurons (Fig. 4C). New neurite measurements in neurons stained by other means are necessary to confirm the results shown.

4. DYRK1A kinase activity in DS-IPCs treated with 10 M EGCG or infected with shRNA DYRK1A lentiviruses decreases around 30-40%. It will be nice to show that the same treatment/infection produces similar effects in N-IPCs.

5. It has been shown that DYRK1A prevents apoptosis under certain conditions by phosphorylation of one of its substrates. How the authors explain the results shown in Fig. 5F and L?

6. DYRK1A is a dosage-dependent gene. Its role in neurodevelopment has been indicated by the phenotypes of mutant flies and mice and, more recently, by the identification of mutations involving DYRK1A in cases of primary microcephaly. The authors should consider this information to interpret and discuss their data.

7. The bibliography cited in the manuscript needs a revision. Some of the references are incorrect or do not correspond to the first or most relevant work indicated in the text. Examples of these are the

following: Marti et al, 2003 (Introduction and Discussion sections); Wegiel et al, 2011 (Introduction section), Lu et al. 2002 and Altafaj et al. 2001 (Discussion Section).

Minor Issues:

- SOX2 expression is not shown in Fig 1C as it is written in the text (page 4).
- Correct "Fig. 4J and 4K" (page 7, lines 1-2); "Fig S4" (page 8, line 2); "Fig 5A-D" (page 8, line 7).
- Consider change the first sentence of the last paragraph in page 7.
- Sentences in page 5 (lines 25-26) and in page 6 (lines 9-10) are incomplete or not clear.
- Glial markers include markers of the astroglial and oligodendroglial lineages. For consistency "Glial and Oligodendrocyte markers" in Fig 4B and in the Discussion section (page 10) should be changed to "Glial markers".
- Asterisks indicating significant differences are missing (or are too small). See for instance Fig. 3D, F and G.

Referee #3 (Comments on Novelty/Model System):

Major problem with this paper was lack of statistical detail

Referee #3 (Remarks):

This was a very interesting paper using iPS cells derived from identical twins discordant for Ch21 trisomy. The paper was well written and the results of great interest with regard to understanding more about the pathogenesis of Down Syndrome.

The two major problems with the paper were related to reference to previous studies and statistical analysis related to iPS technology.

1. The authors cite the study of Bahn et al as a description of "post mortem fetal tissue". In fact this study was an extensive description of how neural progenitors derived from DS post mortem fetal tissue grow and differentiate and had many similarities to the current study including a full transcriptome analysis. More effort should be made to relate the changes shown in this study (which used three independent DS cases compared to 3 controls and included many of the same gene changes and lack of neurogenesis phenomenon as described in the current study) with the current study.

2. A new study from Bhattacharya et al (not cited) also discovered that many of the neurogenesis changes are not seen at early stages of neural development but only later stages. This is also true for DS brain tissue which develops relatively normally until 20 weeks gestation. As ES cells have been shown to develop along long time lines related to human development (Su Chun Zhang papers) the authors should mention why their model seems to show major changes at such an early stage of development.

3. The major problem though with the current MS was lack of detail over iPS clone production and lack of any statistical clarity. For iPS production the authors are still using cMYC which is now known to cause many problems. It was not clear from their methods section whether they were using integrating or non integrating methods but the reviewer assumes integrating - which brings up further issues with regard to bleed through of cMYC affecting differentiation of individual clones.

4. How many clonal lines did the authors produce from each DS sample? They should have used a minimum of three clones from both the control and trisomy twins. All data should then be presented as mean variation across the three clones. All recent reports suggest significant variation between clones from the same iPS lines (in particular with integrating vectors) and so the authors need to show first how much variation in neuronal differentiation there is between clones before choosing one clone from the DS and control fibroblasts for the study.

5. Non of the many graphs and tables had any "n" associated with them. This is a fairly remarkable omission and makes the data impossible to review in it's current form. The N for each experiment

needs to be clearly delineated and described (was it individual clones, experiments, wells from a single dish???). there is absolutely no detail for this crucial part of the study. Again in the data as described could simply be variations between two clones and unrelated to DS.

1st Revision - authors' response 24 September 2013

Referee #1 (Comments on Novelty/Model System):

In this study, the authors established an induced pluripotent stem cell (iPSC) model to study Down syndrome related neurodevelopmental defects. Pluripotent stem cells were generated from monozygotic twins discordant for trisomy 21 thereby eliminating the influence of variable genetic background. The authors used state of the art technologies for validating and determining the transcriptional signature of iPSC lines. They confirmed previous observations on reduced neurogenesis and deficient neuronal differentiation in Down syndrome (DS) cultures. The data on the role of DYRK1A are novel and interesting. The expression of this enzyme was significantly increased in Twin-DS cultures. The pharmacological inhibition or the knockdown of the expression of this enzyme partially corrected the deficit in neurogenesis and neuronal differentiation. Overall, the actual insight for understanding the pathogenic mechanisms remains weak.

1. The authors demonstrate a reduced number of SOX2/Nestin positive NPCs and Ki67 positive cells in DS derived cultures. Ki67 was not co-localized with cell type-specific antigenic markers. Since these preparations likely to represent a mixed population of cells including pluripotent stem cells, multipotent neural progenitors and committed neuroblasts, the authors should provide additional data on the proliferation rate of these different populations. For this purpose, Ki67 or BRDU should be co-localized with cell type-specific markers.

Immunofluorescence analysis of OCT4 and NANOG expression in NPCs derived from Twin-N-iPSCs and Twin-DS-iPSCs revealed the absence of cells positive for OCT4 and NANOG after 3 weeks of neural induction which excludes the presence of residual undifferentiated iPSCs in the neurospheres (Supporting information Fig S5A). The qRT-PCR analysis of *NANOG* and *OCT4* after 3 weeks of neural induction showing the decline of their expression confirmed these results (Fig 3C). Indeed, as outlined by reviewer 1, immunostaining of the preparation revealed a mix population of cells including multipotent neural progenitors (NESTIN$^+$ cells), committed astroglial progenitor cells (GFAP$^+$ cells), committed oligodendroglial progenitor cells (OLIG2$^+$ cells) and committed neuronal cells (β3-TUBULIN$^+$ cells). Our analysis further revealed a reduced proportion of NESTIN$^+$ cells (Fig 3H) and an increased of GFAP$^+$ and OLIG2$^+$ cells upon neural induction of Twin-DS-iPSCs (Fig S5B of Supporting information). These results are consistent with the greater expression of astroglial and oligodendroglial markers revealed by quantitative RT-PCR analysis (Fig 3G). Moreover, the proportion of committed neuronal cells had a tendency to be higher in neurospheres derived from Twin-DS-iPSCs but failed to reach significance (Fig S6 of Supporting information).

As recommended by reviewer 1, we also investigated the proliferation status of each population using co-immunostaining of NESTIN$^+$ cells, GFAP$^+$ cells, OLIG2$^+$ cells and β3-TUBULIN$^+$ cells with Ki67. Committed neuronal astroglial, oligodendroglial and neuronal cells showed no expression of Ki-67, the only cells positive for Ki-67 were NESTIN$^+$ (Fig S6A, S6B and S6C of Supporting information). Importantly, we found a reduced proportion of NESTIN and Ki-67 double positive cells in neurospheres derived from Twin-DS-iPSCs which indicate a reduced proliferation of these cells (Fig S6D of Supporting information). Collectively, these results suggest that the reduction of the multipotent neural progenitor cells and the premature lineage specification into astroglial and oligodendroglial progenitors (and to a less extent into neuronal cells) for Twin-DS-iPSC-derived cells is likely contributing to the reduced proportion of NPCs and to the proliferation deficit observed in cells derived from Twin-DS-iPSCs upon neural induction (Fig 3I).

2. Similarly, it would be important to know which cell type displays increased levels of apoptotic markers.

Similarly, we performed co-immunolocalization of $NESTIN^+$ cells, $GFAP^+$ cells, $OLIG2^+$ and β3-$TUBULIN^+$ cells with an antibody against cleaved caspase-3. Importantly, our results support that $GFAP^+$ cells, $OLIG2^+$ cells and β3-$TUBULIN^+$ cells were negative for cleaved caspase-3 (Fig S6E, Fig S6F and Fig S6G of Supporting information) which indicates that these cells likely did not contribute to the increased caspase-3 activity found in cells derived from Twin-DS-iPSCs upon neural induction (Fig 3K). In contrast, we found a greater proportion of NESTIN and cleaved capase-3 double positive cells which is consistent with an increase of apoptosis in these cells (Fig S6E and Fig S6H of Supporting information). Collectively, these results suggest that the multipotent neural progenitor cells are likely the population that contributes to the increased apoptosis found in cells derived from Twin-DS-iPSCs upon neural induction (Fig 3J and 3K).

3. The data on altered differentiation and maturation of neurons are rather sketchy. The possible shift in the proportion of neuronal subtypes in DS versus control cultured has not been explored. It is possible that trisomy 21 favours glial as well as GABA-ergic differentiation at the expense of the glutamatergic neuronal population. Changes in cell type specification could influence the readout of neuronal process length and arborization measures.

As recommended by reviewer 1, we investigated the proportion of GABA-ergic and glutamatergic neurons by immunostaining with antibodies against GAD67, an enzyme responsible for the synthesis of GABA in neurons, and PSD95, a post synaptic protein expressed in glutamatergic neurons. Notably, we found a reduced density of PSD95 (Fig 5F) and a lower expression of *PSD95* transcripts (Fig 5G). In contrast we found a greater expression of *GAD67* transcripts in neurons derived from Twin-DS-iPSCs (Fig 5G). In line with this, the expression of GAD67 protein had a tendency to be higher in neurons derived from Twin-DS-iPSCs but failed to reach significance (Fig 5F).

4. A key issue is the capacity of neurons to form synaptically interconnected networks. The authors measured some synaptic proteins and emphasized synaptic alterations in Twin-DS cultures. However, they do not demonstrate that iPSC derived neurons could make synaptic contacts, do not explore synaptic functions and do not investigate the possibility that these structures could be different between DS and control cultures.

As recommended by reviewer 1, we also investigated the expression of SYNAPSIN in neurons derived from iPSCs by immunofluorescence confirming that these neurons make synaptic contacts. Interestingly, MAP2-positive dendrites in neurons derived from Twin-DS-iPSCs exhibited a reduced density of SYNAPSIN punctae (Fig 4E) and a reduced expression of *SYN1* transcripts (Fig 4F).

Moreover, we agree that providing data on synaptic function is interesting, considering i) the results reported in our study regarding the proportion of excitatory glutamatergic synapses and of inhibitory GABA-ergic synapses ii) the imbalance between excitatory and inhibitory synapses that has been proposed to explain the cognitive impairment in DS (Kleschevnikov *et al.* J Neurosci 2004; Belichenko *et al.* J Comp Neurol 2007, Chakrabarti *et al.* J Neurosci 2007; Martinez-Cué *et al.* J Neurosci 2013). However, we believe that it is beyond the scope of current study and should be investigated in a new study (as proposed in the discussion section). Indeed, these specific experiments would be quite challenging and would warrant a delay in publishing our data. Upon completion of these experiments, we plan to draft a separate manuscript.

5. Although, it has been predicted that the pharmacological inhibition of DYRK1A may have a potential role in DS therapy (for example see Mazur-Kolecka et al., 2012) (this reference should be cited), the present study provides the first direct support to this hypothesis in a human cell culture model. DYRK1A have established roles in the sequential steps of neurogenesis and neuronal differentiation. The impact of inhibiting DYRK1A on early events including neuronal proliferation and cell death appears obvious. The authors should provide additional data on effects of this treatment on late events including neuronal process formation and synaptogenesis using a delayed targeting of DYRK1A, during neuronal

differentiation after the proliferative phase and neuronal commitment. This information would be essential for evaluating the therapeutic potential of this treatment of DS in adulthood.

As suggested by Reviewer 1, we have acknowledged the publication of Mazur-Kolecka *et al.* (Mazur-Kolecka *et al.* J Neurosci Res 2012), which documented the effect of DYRK1A activity inhibition through harmine on the development of NPCs isolated from DS mice.

We agree that providing data on the effect of DYRK1A inhibition at late events of the neuronal processes using a delayed targeting of DYRK1A or by pharmacological approaches is interesting for therapeutic strategies of DS in adulthood. For that, NPCs derived from Twin-DS-iPSCs were treated with EGCG 10 µM during neuronal differentiation (Fig S9A of Supporting information). Interestingly, we did not find a significant improvement of the number of MAP2 positive cells with EGCG treatment (Fig S9B of Supporting information). However, we found a slight increase in the number of neurites and of the density of SYNAPSIN punctae in neurons derived from Twin-DS-iPSCs upon EGCG treatment (Fig S9C and S9D of Supporting information). Altogether, this indicates that late targeting of DYRK1A with EGCG treatment did not improve the number of neurons derived from Twin-DS-iPSCs but promoted dendritic development and the density of SYNAPSIN in these neurons. These effects are in accordance with the pleiotropic roles and targets of DYRK1A during the sequential stages of neurodevelopment. These results provide also potential opportunities for therapy through DYRK1A inhibition not only in fetal life but also after in late neurodevelopmental stages in patients with DS (considered in the discussion section).

6. It is not clear to me whether multiple comparisons or simple t-tests were performed to analyze data in Fig 5 and 6.

We thank reviewer 1 for the helpful comments regarding the statistical analysis, which we have considered in the revised version of our manuscript (legend and material and method sections). In Fig 5 and Fig 6, statistical analysis among groups was evaluated by one-way ANOVA followed Tukey's post hoc test, and comparisons between two groups by Student's *t*-test.

Referee #2 (Comments on Novelty/Model System):

The use of iPSCs is what makes this work novel and significant in Down syndrome research. Quality of the experiments is OK, although additional details about the methodology used and the number of biological replicates done are required.

This manuscript reports the comparison of the transcriptome and differentiation potential of iPSCs derived from fetal fibroblast isolated from monozygotic twins, one with a normal kariotype and the other with and extra chromosome 21. It also shows that neural progenitor cells (NPCs) derived from trisomic iPSCs express aberrant levels of neural and glial markers and they proliferate less and are less neurogenic than NPCs derived from euploid iPSCs. The authors went on and tested whether they could rescue some of the defects observed in trisomic NPCs by knocking down one of the chromosome 21 genes, DYRK1A, using shRNA or by pharmacological inhibition of DYRK1A kinase activity.

The experiments are well designed and the results novel. Characterization of the iPSC lines is rigorous. The reported cell lines represent an excellent model to study the impact of an extra chromosome 21 in different cell types and to identify the pathways and genes involved in Down syndrome. The results presented on DYRK1A are also significant showing for the first time the effect of an extra copy of the gene in the behavior of human trisomic NPCs.

Comments and questions:

1. The kariotype of one Twin-N and one Twin-DS iPSC lines is shown in Fig 2A. Did the authors check the kariotype of the neurosphere-derived NPCs used in the differentiation experiments? How many cell lines of the each genotype were used in the experiments? The authors should provide this information and

indicate in the Method section or in the legend to the figure the number of independent cell lines used, the number of independent experiments done and the number of replicates in each experiment.

As recommended by reviewer 2, we have done karyotype analysis of NPCs derived from the iPSCs (Fig R1). The neurospheres derived from Twin-DS-iPSCs showed the characteristic trisomy 21 anomaly while those derived from Twin-N-iPSCs had a normal karyotype. This analysis also showed the absence of chromosomal aberrations upon neural induction.

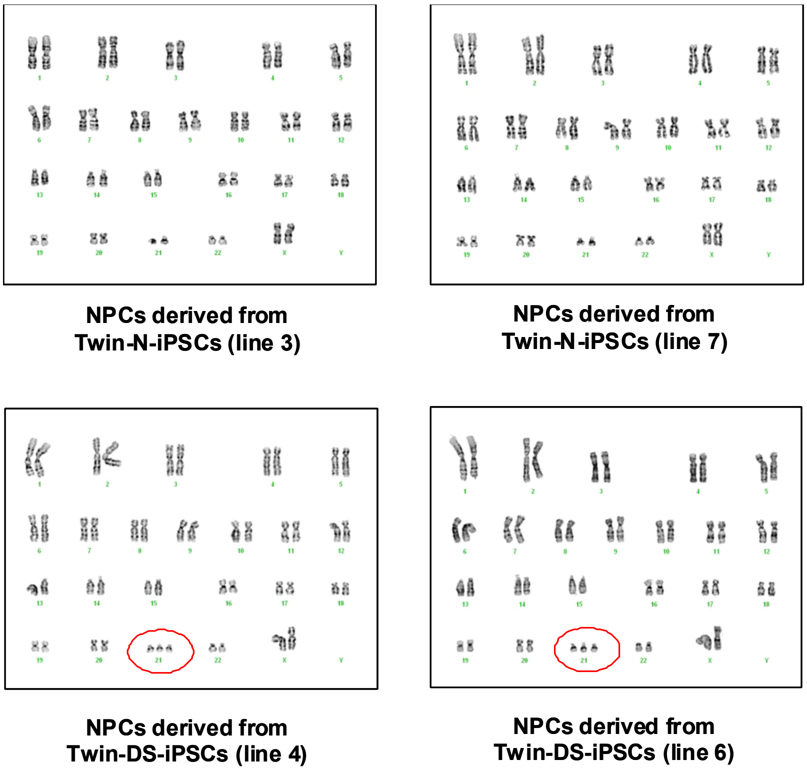

Figure R1. Additional controls showing the karyotypes of NPCs derived from Twin-N-iPSC and Twin-DS-iPSC lines.

Karyotypes of NPCs derived from Twin-N-iPSC and Twin-DS-iPSC lines are 46, XX and 47, XX+ 21, respectively.

We have also added precisions regarding the number of iPSC lines derived from the parental twin fibroblasts and details on the number of replicates and experiments in the figure legend, the material and method and the Supporting information sections. In the present study, several lines were generated from the parental fetal fibroblasts of monozygotic twins discordant for trisomy 21. Among those, lines that we did not succeed to expand or that displayed chromosomal abnormalities (such as trisomy 12 or monosomy X) were excluded. We considered two DS-iPSC and two N-iPSC lines that answered to the following criteria:

- *Karyotyping*
- pluripotency marker expression
- transgene silencing after initial expansion of few passages
- *In vivo* differentiation in a teratoma assay
- *In vitro* potential to generate the three embryonic germ layers when differentiate into embryoid bodies
- *In vitro* potential to generate NPCs and neurons.

Then, one iPSC line for each condition has been used for further analysis.

2. One of the interesting results shown in the manuscript is the increase in apoptosis in DS-NPC cultures. Is apoptosis in these cells also increased when cultured in differentiation media? In other word, are N-NPC and DS-NPC cultures equally dense after the differentiation period? As differentiation and maturation of neurons and glial cells depends on cell density this information may be relevant for the interpretation of the results shown in Fig 4.

As recommended by reviewer 2, we further discuss our results regarding apoptosis during neural induction and neuronal differentiation of Twin-N-iPSCs and Twin-DS-iPSCs in the discussion section.

After neural induction protocol (3 weeks), NPCs were gently dissociated and plated at the same density (~25000 cells/cm^2) after exclusion of dead cells with trypan blue staining and induced to differentiate into neurons for additional 4 weeks of culture. Importantly, after these 4 weeks of neuronal differentiation of these NPCs, we did not see difference in the density of cells between normal and DS cultures (density of nuclei and proportion of abnormal nuclei with Hoechst staining in the picture of Fig 4A) but rather a different proportion of neuronal, astroglial and oligodendroglial cells which is likely the consequence of the generation of less neurogenic but more gliogenic NPCs upon neural induction of Twin-DS-iPSCs (Fig 3F and 3G, Fig S5 of Supporting information). This suggests that NPCs derived from Twin-DS-iPSCs fails to generate the same number of neurons due to defects in the NPCs rather than in survival of the generated neurons after 4 weeks of neuronal differentiation (Fig 4A and 4B). These results are in accordance with morphometric studies of DS brain tissue (Guidi *et al.* Brain Pathol 2008; Griffin *et al.* Neurobiol Aging 1998) and with studies using *in vitro* culture of fetal DS NPCs (Bahn *et al.* Lancet 2002; Esposito *et al.* Hum Mol Genet 2008; Lu *et al.* Plos One 2011; Lu *et al.* Hum Mol Genet 2012).

However, we cannot exclude that neurons derived from Twin-DS-iPSCs could be more susceptible to apoptosis after long term culture (> 4 weeks of neuronal differentiation), reflecting late occurring neurodegeneration observed in DS patients. Indeed, DS individuals show early onset of AD (Antonarakis *et al.* Nat Rev Genet 2004, Lott and Dierssen Lancet Neurol 2010). In this regard, according to recent evidence neurons derived from DS iPSCs cultured more than 100 days exhibited a greater secretion of amyloid peptides, tau protein phosphorylation and cell death (Shi *et al.* Sci Transl Med 2012).

3. B3 TUBULIN immunostaining in DS-neurons is very faint compared to the immunostaining in N-neurons (Fig. 4C). New neurite measurements in neurons stained by other means are necessary to confirm the results shown.

As recommended, we have replaced the image of β3-TUBULIN immunostaining in DS-neurons of Fig 4C. Also, new neurite measurements in neurons stained by MAP2 antibody have been performed and confirmed the reduced number of neurites from soma of neurons derived from Twin-DS-iPSCs in comparison with those derived from Twin-N-iPSCs (Fig R2).

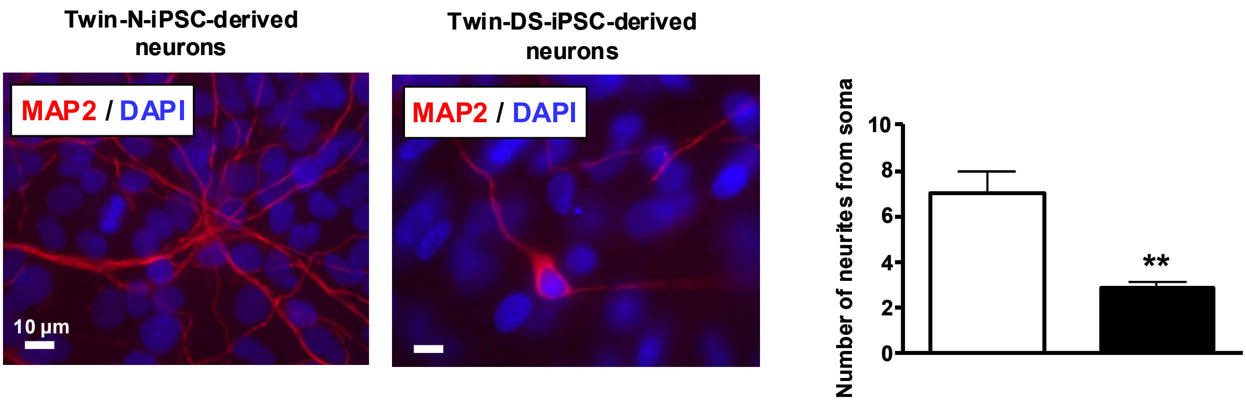

Figure R2. Neurite measurements in neurons derived from Twin-N-iPSCs and Twin-DS-iPSCs

Representative images and quantitative analysis of neurites (either axons or dendrites) from the soma of MAP2 positive neurons derived from Twin-N-iPSCs and Twin-DS-iPSCs. Data are represented as mean ± SEM. ** $p < 0.01$ by Student's *t*-test from n = 3.

4. DYRK1A kinase activity in DS-IPCs treated with 10μM EGCG or infected with shRNA DYRK1A lentiviruses decreases around 30-40%. It will be nice to show that the same treatment/infection produces similar effects in N-IPCs.

As suggested by reviewer 2, knockdown of DYRK1A was achieved using shRNA in Twin-N-iPSCs (Fig R3A). Knockdown efficiencies of DYRK1A shRNA in NPCs derived from Twin-N-iPSCs were analyzed by non quantitative and quantitative RT-PCR (Fig R3B and R3C). As shown in Fig R3D, this lead to a significant reduction of DYRK1A activity. Similarly, Twin-N-iPSCs were treated with 10 μM EGCG during the protocol of neural induction (Fig R3E). This lead to a significant reduction of DYRK1A in NPCs derived from Twin-N-iPSCs (Fig R3F).

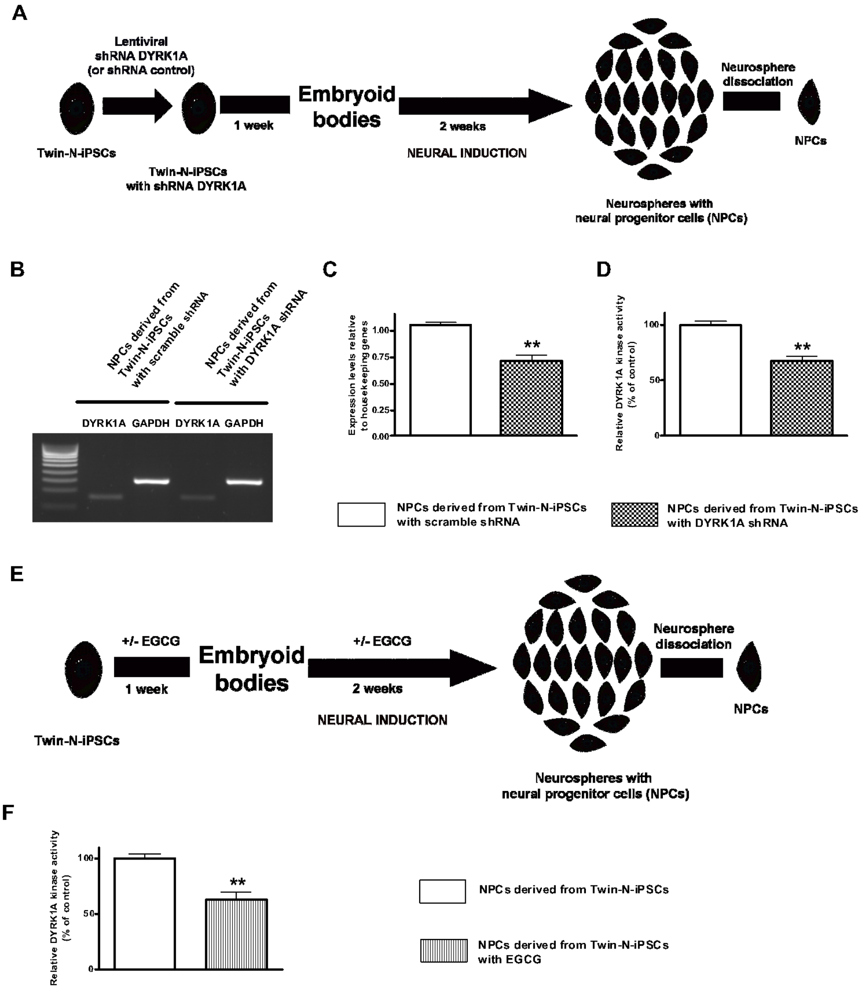

Figure R3. Additional controls showing the efficiency of DYRK1A inhibition through EGCG treatment and through DYRK1A shRNA in NPCs derived from Twin-N-iPSCs.

(A) Schematic representation for generation of NPCs from Twin-N-iPSCs after transduction with shRNAs targeting *DYRK1A*. (B, C) Non quantitative and quantitative RT-PCR showing knockdown efficiencies of DYRK1A shRNA in NPCs derived from Twin-N-iPSCs. (D) Effect of DYRK1A inhibition through DYRK1A shRNA in NPCs derived from Twin-N-iPSCs. (E) Schematic representation for generation of NPCs from Twin-N-iPSCs after incubation with EGCG 10 μM. (F) Effect of DYRK1A inhibition through EGCG treatment in NPCs derived from Twin-N-iPSCs. Data are represented as mean ± SEM. ** $p < 0.01$ by Student's *t*-test from 3-4 independent experiments.

5. It has been shown that DYRK1A prevents apoptosis under certain conditions by phosphorylation of one of its substrates. How the authors explain the results shown in Fig. 5F and L?

We thank reviewer 2 for the helpful comments, which we have considered in the revised version of our manuscript (in the discussion section).

Contrary to the numerous reported roles of *DYRK1A* in the control of cell cycle, very little is known regarding its impact on cell death. For instance, DYRK1A has been shown to prevent the intrinsic apoptotic pathway through the phosphorylation of caspase-9 during retina development (Laguna *et al.* Dev Cell 2008). In contrast, the overexpression of *DYRK1A* makes rat embryonic hippocampal progenitor cells more susceptible to apoptosis by phosphorylating and activating p53 which lead to the subsequent upregulation of p53 target genes and proteins such as FAS (CD95) (Park *et al.* J Biol Chem 2010). This is of special interest as the protein levels of the pro-apoptotic genes *p53* and *FAS* are increased in the cerebral cortex and the cerebellum of DS patients (De la Monte *et al.* Lab invest 1998; Seidl *et al.* Neurosci Lett 1999). It remains to be established whether p53 and FAS underlie the increased apoptosis induced by *DYRK1A* overexpression in NPCs derived from Twin-DS-iPSCs. Collectively, these studies and our results strongly support the idea that the regulation of apoptosis by DYRK1A plays an important role in development and pathogenesis.

6. DYRK1A is a dosage-dependent gene. Its role in neurodevelopment has been indicated by the phenotypes of mutant flies and mice and, more recently, by the identification of mutations involving DYRK1A in cases of primary microcephaly. The authors should consider this information to interpret and discuss their data.

We thank reviewer 2 for the helpful comments, which we have considered in the revised version of our manuscript (in the discussion section).

Indeed, both loss and gain of function of *DYRK1A* result in neurodevelopmental defects. DYRK1A$^{-/-}$ null mutant mice show growth delay and die during midgestation whereas mice heterozygous for DYRK1A mutation (DYRK1A$^{-/+}$) show a decreased neonatal viability and body size reduction from birth to adulthood. Also, DYRK1A$^{-/+}$ mice display a brain size 30% smaller than wild type with a region-specific reduction of neurons: in particular, alteration of neocortiocal pyramidal cells, the most represented neurons in the cortex has been described in these mice (Fotaki V *et al.* Mol Cell Biol 2002; Benavides-Piccion R *et al.* Neuobiol Dis 2005). In humans, *DYRK1A* haploinsufficiency is associated with microcephaly as well as growth and mental retardation (Moller RS *et al.* Am J Hum Genet 2008; Yamamoto T *et al.* Am J Med Genet 2010; Valetto A *et al.* Eur J Med Genet 2012; Van Bokhoven H *et al.* Ann Rev Genet 2011). These studies further support that DYRK1A plays a crucial role in development and pathogenesis. The expression levels of this gene has to be tightly regulated during development as both up and down-regulation of its expression lead to neurodevelopmental defects.

7. The bibliography cited in the manuscript needs a revision. Some of the references are incorrect or do not correspond to the first or most relevant work indicated in the text. Examples of these are the following: Marti et al, 2003 (Introduction and Discussion sections); Wegiel et al, 2011 (Introduction section), Lu et al. 2002 and Altafaj et al. 2001 (Discussion Section).

Minor Issues:
- SOX2 expression is not shown in Fig 1C as it is written in the text (page 4).
- Correct "Fig. 4J and 4K" (page 7, lines 1-2); "Fig S4" (page 8, line 2); "Fig 5A-D" (page 8, line 7).
- Consider change the first sentence of the last paragraph in page 7.
- Sentences in page 5 (lines 25-26) and in page 6 (lines 9-10) are incomplete or not clear.
- Glial markers include markers of the astroglial and oligodendroglial lineages. For consistency "Glial and Oligodendrocyte markers" in Fig 4B and in the Discussion section (page 10) should be changed to "Glial markers".
- Asterisks indicating significant differences are missing (or are too small). See for instance Fig. 3D, F and G.

As suggested by Reviewer 2, we have acknowledged the original publications or the most relevant work. For example, we have deleted the study of Marti *et al.* Brain Res 2003, Wegiel *et al.* FEBS J 2011, Becker

et al. FEBS J 2011, Liu et al. 2002 and added the following studies Tejedor *et al.* Neuron 1995, Smith *et al.* Nature Genet 1997, Guimera *et al.* Hum Mol Genet 1996, Song *et al.* Genomics 1996, Becker *et al.* J Biol Chem 1998 (and other studies as recommended by the other reviewers).

Moreover, we have considered the minor issues outlined by reviewer 2 in the revised version of our manuscript.

Referee #3 (Comments on Novelty/Model System):

Major problem with this paper was lack of statistical detail

Referee #3 (Remarks):
This was a very interesting paper using iPS cells derived from identical twins discordant for Ch21 trisomy. The paper was well written and the results of great interest with regard to understanding more about the pathogenesis of Down syndrome.

The two major problems with the paper were related to reference to previous studies and statistical analysis related to iPS technology.

We thank Reviewer 3 for the helpful comments, which we have considered in the revised version of our manuscript. As recommended by Reviewer 3, we have added comments concerning our findings and precisions regarding the statistical analysis in the discussion, material & method and figure legend sections.

1. The authors cite the study of Bahn et al as a description of "post mortem fetal tissue". In fact this study was an extensive description of how neural progenitors derived from DS post mortem fetal tissue grow and differentiate and had many similarities to the current study including a full transcriptome analysis. More effort should be made to relate the changes shown in this study (which used three independent DS cases compared to 3 controls and included many of the same gene changes and lack of neurogenesis phenomenon as described in the current study) with the current study.

2. A new study from Bhattacharya et al (not cited) also discovered that many of the neurogenesis changes are not seen at early stages of neural development but only later stages. This is also true for DS brain tissue which develops relatively normally until 20 weeks gestation. As ES cells have been shown to develop along long time lines related to human development (Su Chun Zhang papers) the authors should mention why their model seems to show major changes at such an early stage of development.

<u>Answer to points 1 and 2:</u>

As recommended by reviewer 3, we further discuss our results with those from Bahn *et al.* in the discussion section. In this study, Bhan *et al.* showed that NPCs isolated from fetal DS brain (8-18 weeks of gestation) generate fewer neurons in comparison with normal ones (Bahn *et al.* Lancet 2002). These neurons exhibited also reduced neurite from soma which is consistent with our findings using normal and DS iPSCs differentiated into NPCs and neurons. However, in contrast with our study, the differentiation of these NPCs revealed no difference in the proportion of glial cells. We also discuss more the results regarding *REST/NRSF* target genes described in this study.

As recommended, the study of Bhattacharya *et al.* is cited in the revised version of the manuscript (in the introduction and discussion sections). In this study, Bhattacharya *et al.* showed that NPCs isolated from fetal DS brain (13-18 weeks of gestation) expanded in culture for less than 6 weeks, generate the same proportion of neurons as the normal counterpart. However, when these DS-NPCs were expanded in culture more than 10 weeks, they generate fewer neurons in comparison with N-NPCs. The astroglial and oligodendroglial shifts demonstrated in our study were not investigated in the study of Bhattacharya *et al.* however an upregulation of oligodendroglial markers was found by microarray analysis of DS NPCs (*OLIG1*, *OLIG2* and *OMG*).

Regarding the onset of the neurodevelopmental defects in DS patients, conflicting results have been published. While some studies report that DS brain tissue develop normally until 19-23 weeks of gestation

© EMBO

as revealed by both morphometric analysis of brain tissue (Weitzdoerfer R *et al.* Neural Transm Suppl 2001) and by studies using *in vitro* culture of NPCs isolated from brain fetuses (13W-18W of gestation, Bhattacharya *et al.* Dev Neurosci 2009), others have shown opposite results in both DS brain fetuses (17W-21W of gestation, Guidi *et al.* Brain Pathol 2008; 17W-35W of gestation, Griffin *et al.* Neurobiol Aging 1998) and in isolated NPCs from DS brain fetuses (8W-18W of gestation, Bahn *et al.* Lancet 2002; Esposito *et al.* Hum Mol Genet 2008; 14W-18W of gestation, Lu *et al.* Plos One 2011; 14W-21W of gestation Lu *et al.* Hum Mol Genet 2012, 19W-21W of gestation). These discrepancies may be attributable to difference in the brain region origins and the different gestational ages of the fetal tissue. Also, factors such as the methodologies used for isolation, maintenance and differentiation of these NPCs may account for the seemingly discrepancies. Our results are more consistent with the latter studies. For instance, compared to normal counterpart, brain from DS fetuses exhibited a smaller percentage of cells with neuronal phenotype but a higher percentage of cells with astrocytic phenotype in the dendate gyrus, the hippocampus and the parahippocampal gyrus (Guidi *et al.* Brain Pathol 2008). Similarly, *in vitro* culture of fetal DS NPCs showed a reduction of neuron density (Bahn *et al.* Lancet 2002) and an increase of astroglial and oligodendroglial cells (Mito and Becker Exp Neurol 1993; Griffin *et al.* Neurobiol Aging 1998; Esposito *et al.* Hum Mol Genet 2008; Lu *et al.* Plos One 2011; Lu *et al.* Hum Mol Genet 2012).

Moreover, the presence of a broad phenotypic variability among DS individuals is also a contributor of the discrepancies among studies. In this respect, trisomy 21 can have differential pathogenicity on individual genomes even though they share some morphogenetic characteristics. We have previously shown that the variations of expression of HSA21 genes in DS cells are important determinants of the phenotypic variability of DS (Prandini P *et al.* Am J Hum Genet 2007). This could lead to incompletely to more penetrant phenotype. In this respect, in the study of Bhattacharyya *et al.* DS NPCs did not exhibit overexpression of several HSA21 genes including *DYRK1A*, *SOD1*, *APP*, *DSCAM*, *S100B*. This contrasts with our cellular model. Considering the crucial role of *DYRK1A* in neurodevelopment and DS pathogenesis demonstrated in our study and previous reports (reviewed in Tejedor and Hammerle 2011), this could explain at least in part, the discrepancies between the results. In addition, the HSA21 genes *S100B* and *APP* has been reported as main contributors of the glial shift in DS brain tissue (Esposito *et al.* Hum Mol Genet 2008; Lu *et al.* Plos One 2011).

Finally, the possibility also exists that differences between control and DS fetuses are undetectable because of inter-individual variance within groups of fetal tissue. The type and the genetic background of the cells used in the present study and the studies of Bahn *et al.* and Bhattacharya *et al.* are likely to contribute to these differences: NPCs isolated from brains from unrelated healthy and DS fetuses versus iPSCs, NPCs and neurons with the same genetic background. In our study, the generation of iPSCs, NPCs and neurons from monozygotic twins discordant for trisomy 21 offer a unique opportunity to study neurodevelopment in DS given that except for the supernumerary HSA21, the rest of genome is identical between the twins.

3. The major problem though with the current MS was lack of detail over iPS clone production and lack of any statistical clarity. For iPS production the authors are still using cMYC which is now known to cause many problems. It was not clear from their methods section whether they were using integrating or non integrating methods but the reviewer assumes integrating - which brings up further issues with regard to bleed through of cMYC affecting differentiation of individual clones.

4. How many clonal lines did the authors produce from each DS sample? They should have used a minimum of three clones from both the control and trisomy twins. All data should then be presented as mean variation across the three clones. All recent reports suggest significant variation between clones from the same iPS lines (in particular with integrating vectors) and so the authors need to show first how much variation in neuronal differentiation there is between clones before choosing one clone from the DS and control fibroblasts for the study.

Answer to points 3 and 4:

As recommended by Reviewer 3, we have added precisions for the protocol used for the generation of the iPSC lines in the material & methods and the Supporting information sections. In this regard, Twin-N-iPSCs and Twin-DS-iPSCs were generated by transducing the parental fibroblasts (Twin-N and Twin-DS) with polycistronic lentiviral vectors expressing *OCT4*, *SOX2, KLF4* and *c-MYC* genes.

The use of *c-MYC* and integrative vectors for the cellular reprogramming into iPSCs offers a relatively more efficient means of reprogramming but also raise concerns as outlined by reviewer 3, with the risk i) of

overexpression of potentially tumorigenic genes such as *c-MYC*, ii) of genetic aberrations and iii) the incomplete silencing of the reprogramming factors following differentiation. For instance, the presence of *c-MYC* is a major limitation for clinical applications as chimeras derived from iPSCs frequently develop tumours due to the reactivation of *c-Myc* (Markoulaki *et al.* Nat Biotech 2009; Okita *et al.* Nature 2007).

Also, the use of integrative approaches has been associated with genomic instability of the generated iPSCs. However, recent evidence shows that similar frequency of genetic mutations has been found in iPSCs generated by integrating and non-integrative approaches (Gore *et al.* Nature 2011). Many efforts have been taken to make iPSCs more amenable for clinical applications and disease modelling through exclusion of *c-MYC*, reduction in the number of factors used for reprogramming (Kim *et al.* Cell 2009; Nakagawa *et al.* Nat Biotech 2008) and non-integrating gene delivery approaches (Kim *et al.* Cell Stem Cell 2009; Zhou *et al.* Cell Stem Cell 2009). It is important to note however that such approaches are more labor intensive and less efficient. In fact, *c-MYC* is an important inducer of reprogramming (Sridharan *et al.* Cell 2009; Nakagawa *et al.* Nat Biotech 2008; Judson *et al.* Nat Biotech 2009; Araki R *et al.* Stem Cells 2011), activating pluripotent genes and maintaining the pluripotent state of PSCs (Catwright *et al.* Development 2005; Smith *et al.* Cell Stem cell 2010; Meyer and Penn Nat Rev Cancer 2008). It is considered the driver of the first transcriptional wave during the cellular reprogramming into iPSCs (Polo *et al.* Cell 2012). This could explain at least in part why the vast majority of the reported iPSC lines are achieved using *c-Myc*. At present, there is no clear optimal approach for the reprogramming; each method has strengths and disadvantages. Our iPSCs were generated 3 years ago and at this time non-integrating approaches were not easily available (we are currently using Sendai virus for the reprogramming). Regarding the concerns raised about the methodology used for the reprogramming of our iPSCs, we first verified transgene silencing in Twin-N-iPSC and Twin-DS-iPSC lines after initial expansion of few passages by RT-PCR (Fig S1 of Supporting information). Then, karyotype and CGH array analysis were performed at the undifferentiated iPSC level (Fig 2A and 2B); karyotyping was also performed after neural induction of the iPSCs (Comment 1 of reviewer 2). The characteristic trisomy 21 anomaly was conserved after reprogramming of the parental Twin-DS fibroblasts into Twin-DS-iPSCs and upon neural induction of Twin-DS-iPSCs. Thus, this analysis showed the absence of chromosomal aberrations at the undifferentiated iPSC and at the NPC levels.

Moreover, early reports have proposed that residual transgene expression after using integrating viral approaches may affect pluripotency and differentiation states (Park *et al.* Nature 2008; Yu *et al.* Science 2007). More recently, Hu *et al.* reported variable potency of iPSCs to differentiate into neural cells independently of the set of reprogramming transgenes used to derive iPSCs as well as the presence or not of the reprogramming transgenes in the generated iPSCs (Hu *et al.* Proc Natl Acad Sci 2010). In this elegant study comparing the efficiency of 5 human ESC and 12 human iPSC lines (which were generated by either integrating or transgene-free strategies) to differentiate into neural cells, iPSCs showed significantly lower differentiation into neuroepithelial cells (PAX6 positive cells) than ESCs regardless of the reprogramming method. Thus, the only iPSC line that showed similar efficiency as ESC lines to differentiate into PAX6 positive cells was an iPSC line reprogrammed from fetal cells with lentiviruses, in comparison with those derived from neonatal and adult cells using integrating or transgene-free strategies (Hu *et al.* Proc Natl Acad Sci 2010). In line with this, in the study of Major and colleagues comparing the differentiation potential of iPSC lines derived from a single parental fibroblast line via several reprogramming strategies (+/- *c-MYC*, excised or non excised transgene), neither the presence of *c-MYC* nor the presence of the transgene prevented *in vitro* potential of these iPSCs to differentiate into NPCs, neurons, astrocytes or oligodendrocyte (Major *et al.* PLOS one 2011). From the study of Löhle *et al.*, it appears that omission in iPSCs of reprogramming factors and of *c-MYC* in particular, compromises the efficiency of their subsequent differentiation into NPCs and neurons (Löhle *et al.* Stem Cells 2012).

Considering this, we verified the potential of our iPSC lines to differentiate *in vivo* in a teratoma assay (Fig 3A, Fig S4A and Table 1 of Supporting information), *in vitro* upon spontaneous differentiation into the three germ layers as embryoid bodies (Fig 3B) and *in vitro* directed differentiation into NPCs and neurons.

Numerous studies point to variations between iPSC lines from the same parental somatic cells and the need to generate several lines to be sure that the results is reflecting the disease phenotype and to exclude the possibility that the results shown are due to the selection of specific lines. In the present study, several lines were generated from the parental fetal fibroblasts of monozygotic twins discordant for trisomy 21 (Twin-N and Twin-DS). Among those, iPSC lines that we did not succeed to expand or that presented chromosomal abnormalities were excluded from the study. We considered two DS-iPSC and two N-iPSC lines that answered to the following criteria (Table S1 of supporting Information):

- Karyotyping

- pluripotency marker expression
- transgene silencing after initial expansion of few passages
- *In vivo* differentiation in a teratoma assay
- *In vitro* potential to generate the three embryonic germ layers when differentiate as embryoid bodies
- *In vitro* potential to generate NPCs and neurons.

To be sure that the results observed in the present are related to DS, we first investigated how much variation there is between the 2 lines in each condition in term of potential of these iPSC lines to differentiate into NPCs and neurons (as an example see Fig R4). For further analysis, one iPSC line for each condition has been used.

Finally, the impaired neurogenesis observed in NPCs and neurons derived from Twin-DS-iPSCs couldn't have been due to some independent or unexplained effects of the reprogramming method or selection of specific lines. The appearance of alterations related to several developmental processes (including those related to neurogenesis and neuron differentiation) in the undifferentiated Twin-DS-iPSC lines together with the correction of some neurogenesis defects by DYRK1A inhibition through pharmacological means and shRNA silencing clearly rule out this possibility.

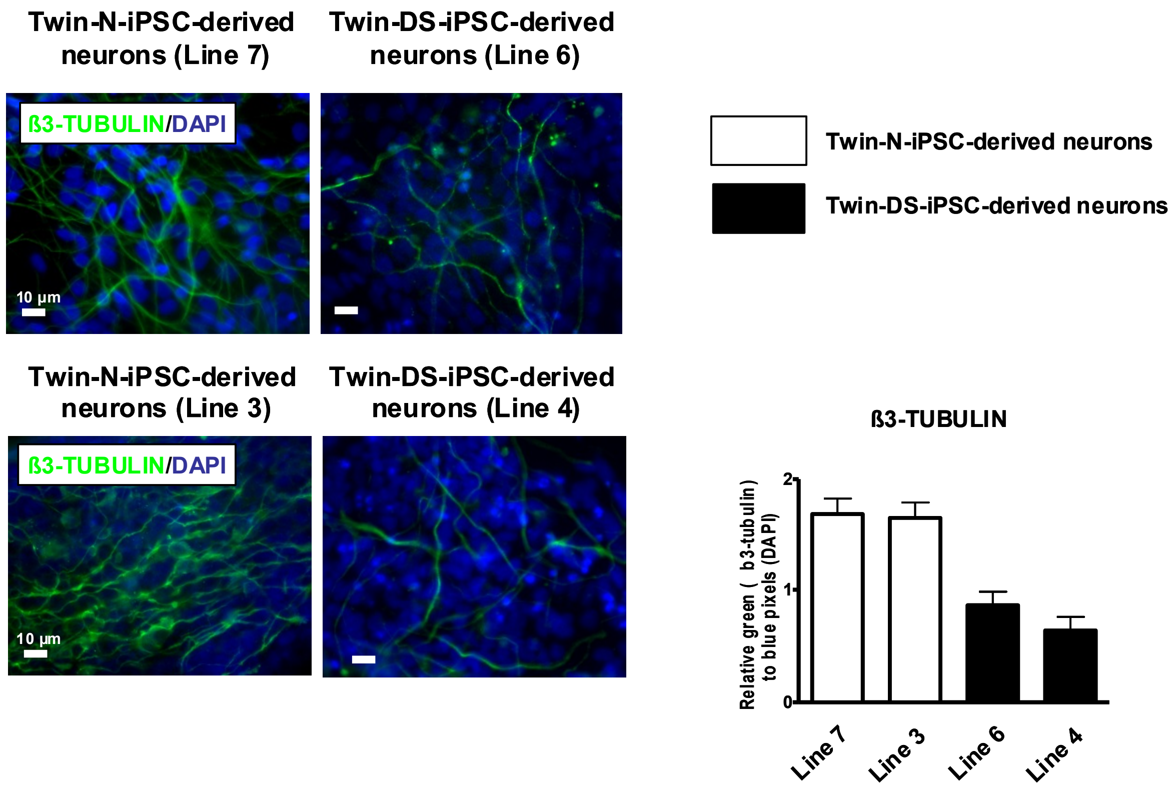

Figure R4. Additional controls showing the potential of Twin-N-iPSC and Twin-DS-iPSC lines to differentiate into neurons.

Quantitative expression of neuronal marker (β3-TUBULIN) after neuronal differentiation of NPCs derived from Twin-N-iPSCs and Twin-DS-iPSCs into neurons, by immunofluorescence analysis. Data are represented as mean ± SEM from n > 4.

5. Non of the many graphs and tables had any "n" associated with them. This is a fairly remarkable

omission and makes the data impossible to review in it's current form. The N for each experiment needs to be clearly delineated and described (was it individual clones, experiments, wells from a single dish???). there is absolutely no detail for this crucial part of the study. Again in the data as described could simply be variations between two clones and unrelated to DS.

We apologize that these important points are not clearly shown in the manuscript. Therefore, we have added precisions regarding the number of experiments done in each result presented in the figures of the revised manuscript. The results presented in the study are from one Twin-N-iPSC and one Twin-DS-iPSC lines but as mentioned in our answer for point 4, the potential to differentiate *in vivo* and *in vitro* of 2 Twin-N-iPSC and 2 Twin-DS-iPSC lines has been investigated.

References:

Antonarakis SE, Lyle R, Dermitzakis ET, Reymond A, Deutsch S (2004) Chromosome 21 and Down syndrome: from genomics to pathophysiology. Nat Rev Genet 5: 725-738

Araki R, Hoki Y, Uda M, Nakamura M, Jincho Y, Tamura C, Sunayama M, Ando S, Sugiura M, Yoshida MA, Kasama Y, Abe M (2011) Crucial role of c-Myc in the generation of induced pluripotent stem cells. Stem Cells 29: 1362-1370

Bhattacharyya A, McMillan E, Chen SI, Wallace K, Svendsen CN (2009) A critical period in cortical interneuron neurogenesis in Down syndrome revealed by human neural progenitor cells. Dev Neurosci 31: 497-510

Cartwright P, McLean C, Sheppard A, Rivett D, Jones K, Dalton S (2005) LIF/STAT3 controls ES cell self-renewal and pluripotency by a Myc-dependent mechanism. Development 132: 885-896

Esposito G, Imitola J, Lu J, De Filippis D, Scuderi C, Ganesh VS, Folkerth R, Hecht J, Shin S, Iuvone T, Chesnut J, Steardo L, Sheen V (2008) Genomic and functional profiling of human Down syndrome neural progenitors implicates S100B and aquaporin 4 in cell injury. Hum Mol Genet 17: 440-457

Gore A, Li Z, Fung H-L, Young JE, Agarwal S, Antosiewicz-Bourget J, Canto I, Giorgetti A, Israel MA, Kiskinis E, Lee J-H, Loh Y-H, Manos PD, Montserrat N, Panopoulos AD, Ruiz S, Wilbert ML, Yu J, Kirkness EF, Belmonte JCI, Rossi DJ, Thomson JA, Eggan K, Daley GQ, Goldstein LSB, Zhang K (2011) Somatic coding mutations in human induced pluripotent stem cells. Nature 471: 63-67

Griffin WST, Sheng JG, McKenzie JE, Royston MC, Gentleman SM, Brumback RA, Cork LC, Del Bigio MR, Roberts GW, Mrak RE (1998) Life-long overexpression of S100β in Down's syndrome: implications for Alzheimer pathogenesis. Neurobiol Aging 19: 401-405

Guidi S, Bonasoni P, Ceccarelli C, Santini D, Gualtieri F, Ciani E, Bartesaghi R (2008) Neurogenesis impairment and increased cell death reduce total neuron number in the hippocampal region of fetuses with Down syndrome. Brain Pathol 18: 180-197

Guidi S, Ciani E, Bonasoni P, Santini D, Bartesaghi R (2011) Widespread proliferation impairment and hypocellularity in the cerebellum of fetuses with Down syndrome. Brain Pathol 21: 361-373

Hu B-Y, Weick JP, Yu J, Ma L-X, Zhang X-Q, Thomson JA, Zhang S-C (2010) Neural differentiation of human induced pluripotent stem cells follows developmental principles but with variable potency. Proc Natl Acad Sci 107: 4335-4340

Judson RL, Babiarz JE, Venere M, Blelloch R (2009) Embryonic stem cell-specific microRNAs promote induced pluripotency. Nat Biotech 27: 459-461

Kim D, Kim C-H, Moon J-I, Chung Y-G, Chang M-Y, Han B-S, Ko S, Yang E, Cha KY, Lanza R, Kim K-S (2009) Generation of human induced pluripotent stem cells by direct delivery of reprogramming proteins. Cell stem cell 4: 472-476

Kim JB, Sebastiano V, Wu G, Araúzo-Bravo MJ, Sasse P, Gentile L, Ko K, Ruau D, Ehrich M, van den Boom D, Meyer J, Hübner K, Bernemann C, Ortmeier C, Zenke M, Fleischmann BK, Zaehres H, Schöler HR (2009) Oct4-induced pluripotency in adult neural stem cells. Cell 136: 411-419

Löhle M, Hermann A, Glaß H, Kempe A, Schwarz SC, Kim JB, Poulet C, Ravens U, Schwarz J, Schöler HR, Storch A (2012) Differentiation efficiency of induced pluripotent stem cells depends on the number of reprogramming factors. Stem cells 30: 570-579

Lott IT, Dierssen M (2010) Cognitive deficits and associated neurological complications in individuals with Down's syndrome. Lancet Neurol 9: 623-633

Lu J, Esposito G, Scuderi C, Steardo L, Delli-Bovi LC, Hecht JL, Dickinson BC, Chang CJ, Mori T, Sheen V (2011) S100B and APP promote a gliocentric shift and impaired neurogenesis in Down syndrome neural progenitors. PLoS ONE 6: e2212

Lu J, Lian G, Zhou H, Esposito G, Steardo L, Delli-Bovi LC, Hecht JL, Lu QR, Sheen V (2012) OLIG2 over-expression impairs proliferation of human Down syndrome neural progenitors. Hum Mol Genet 21: 2330-2340

Major T, Menon J, Auyeung G, Soldner F, Hockemeyer D, Jaenisch R, Tabar V (2011) Transgene excision has no impact on in vivo integration of human iPS derived neural precursors. PLoS ONE 6: e24687

Markoulaki S, Hanna J, Beard C, Carey BW, Cheng AW, Lengner CJ, Dausman JA, Fu D, Gao Q, Wu S, Cassady JP, Jaenisch R (2009) Transgenic mice with defined combinations of drug-inducible reprogramming factors. Nat Biotech 27: 169-171

Meyer N, Penn LZ (2008) Reflecting on 25 years with MYC. Nat Rev Cancer 8: 976-990

Mito T, Becker LE (1993) Developmental changes of S-100 protein and glial fibrillary acidic protein in the brain in Down syndrome. Exp Neurol 120: 170-176

Nakagawa M, Koyanagi M, Tanabe K, Takahashi K, Ichisaka T, Aoi T, Okita K, Mochiduki Y, Takizawa N, Yamanaka S (2008) Generation of induced pluripotent stem cells without Myc from mouse and human fibroblasts. Nat Biotech 26: 101-106

Okita K, Ichisaka T, Yamanaka S (2007) Generation of germline-competent induced pluripotent stem cells. Nature 448: 313-317

Park I-H, Zhao R, West JA, Yabuuchi A, Huo H, Ince TA, Lerou PH, Lensch MW, Daley GQ (2008) Reprogramming of human somatic cells to pluripotency with defined factors. Nature 451: 141-146

Polo Jose M, Anderssen E, Walsh Ryan M, Schwarz Benjamin A, Nefzger Christian M, Lim Sue M, Borkent M, Apostolou E, Alaei S, Cloutier J, Bar-Nur O, Cheloufi S, Stadtfeld M, Figueroa Maria E, Robinton D, Natesan S, Melnick A, Zhu J, Ramaswamy S, Hochedlinger K (2012) A molecular roadmap of reprogramming somatic cells into iPS cells. Cell 151: 1617-1632

Prandini P, Deutsch S, Lyle R, Gagnebin M, Vivier CD, Delorenzi M, Gehrig C, Descombes P, Sherman S, Bricarelli FD, Baldo C, Novelli A, Dallapiccola B, Antonarakis SE (2007) Natural gene-expression variation in Down syndrome modulates the outcome of gene-dosage imbalance. Am J Hum Genet 81: 252-263

Smith KN, Singh AM, Dalton S (2010) Myc represses primitive endoderm differentiation in pluripotent stem cells. Cell stem cell 7: 343-354

Soldner F, Hockemeyer D, Beard C, Gao Q, Bell GW, Cook EG, Hargus G, Blak A, Cooper O, Mitalipova M, Isacson O, Jaenisch R (2009) Parkinson's disease patient-derived induced pluripotent stem cells free of viral reprogramming factors. Cell 136: 964-977

Sridharan R, Tchieu J, Mason MJ, Yachechko R, Kuoy E, Horvath S, Zhou Q, Plath K (2009) Role of the murine reprogramming factors in the induction of pluripotency. Cell 136: 364-377

Tejedor FJ, Hämmerle B (2011) MNB/DYRK1A as a multiple regulator of neuronal development. FEBS J 278: 223-235

Weitzdoerfer R, Dierssen M, Fountoulakis M, G. L (2001) Fetal life in Down syndrome starts with normal neuronal density but impaired dendritic spines and synaptosomal structure. J Neural Transm Suppl: 59-70.

Yu J, Hu K, Smuga-Otto K, Tian S, Stewart R, Slukvin II, Thomson JA (2009) Human induced pluripotent stem cells free of vector and transgene sequences. Science 324: 797-801

Zhou H, Wu S, Joo JY, Zhu S, Han DW, Lin T, Trauger S, Bien G, Yao S, Zhu Y, Siuzdak G, Schöler HR, Duan L, Ding S (2009) Generation of induced pluripotent stem cells using recombinant proteins. Cell stem cell 4: 381-384

© EMBO

2nd Editorial Decision 28 October 2013

Thank you for the submission of your revised manuscript to EMBO Molecular Medicine. We have now received the enclosed reports from the referees that were asked to re-assess it. As you will see the reviewers are now globally supportive and I am pleased to inform you that we will be able to accept your manuscript pending the following final amendments:

-Please address the final (minor) concerns of Referee 2. I would like to particularly stress the importance of providing higher resolution of Figure 5F and increasing the font size of all plots axis labels.

-We would equally need the dataset 2 zipped and provided in a different format than a gz file

-According to our guidelines, we need an ethical statement regarding the use of human derived samples.

-Data of gene expression experiments (RNAseq) and copy number variation (aCGH) described in submitted manuscripts should be deposited in a MIAME-compliant format with one of the public databases. We would therefore ask you to submit your microarray data to the ArrayExpress or GEO databases.

Please submit your revised manuscript within two weeks.

***** Reviewer's comments *****

Referee #1 (Comments on Novelty/Model System):

The authors have performed additional experiments and have adequately answered most of the questions raised in my previous review. The paper is considerably improved.

Referee #2 (Remarks):

The authors have answered to all the points raised in the first revision. They have performed new experiments, expanded the discussion section, and provided information regarding the statistical analysis of the data. They have improved the manuscript significantly and, in my opinion, this should be accepted for publication. Still there are some minor errors and formal aspects of the manuscript that have to be corrected/improved before final acceptance.

Examples of these are:
- The quality of images in Fig. 5F is too poor for publication.
- Font size in most of the histograms [i.e. Y-axis in Fig. 5 B and G] is too small.
- The letters referring to the different panels in the legend for Fig. 4 are not correct.
- Gene symbol for mouse DYRK1A is not correct (page 13).

Referee #3 (Comments on Novelty/Model System):

The authors have addressed my concerns.
